# Axial Ligand Effects on the Mechanism of Ru-CO Bond Photodissociation and Photophysical Properties of Ru(II)-Salen PhotoCORMs/Theranostics: A Density Functional Theory Study

**DOI:** 10.3390/molecules30051147

**Published:** 2025-03-03

**Authors:** Niq Catevas, Athanassios Tsipis

**Affiliations:** Laboratory of Inorganic Chemistry, Department of Chemistry, University of Ioannina, 45110 Ioannina, Greece; n.katevas@uoi.gr

**Keywords:** photoCORMs, theranostics, *trans*-philicity, Ru(II) salen complexes, Ru-CO bond photodissociation

## Abstract

Density functional theory (DFT) calculations were employed to study a series of complexes of general formula [Ru(salen)(X)(CO)]^0/−1^ (X = Cl^−^, F^−^, SCN^−^, DMSO, Phosphabenzene, Phosphole, TPH, CN^−^, N_3_^−^, NO_3_^−^, CNH^−^, NHC, P(OH)_3_, PF_3_, PH_3_). The effect of ligands X on the Ru-CO bond was quantified by the *trans*-philicity, Δ*σ*^13^C NMR parameter. The potential of Δ*σ*^13^C to be used as a probe of the CO photodissociation by Ru(II) transition metal complexes is established upon comparing it with other *trans*-effect parameters. An excellent linear correlation is found between the energy barrier for the Ru-CO photodissociation and the Δ*σ*^13^C parameter, paving the way for studying photoCORMs with the ^13^C NMR method. The strongest *trans*-effect on the Ru-CO bond in the [Ru(salen)(X)(CO)]^0/−1^ complexes are found when X = CNH^−^, NHC, and P(OH)_3_, while the weakest for X = Cl^−^, NO_3_^−^ and DMSO *trans*-axial ligands. The Ru-CO bonding properties were scrutinized using Natural Bond Orbital (NBO), Natural Energy Decomposition Analysis (NEDA) and Natural Orbital of Chemical Valence (NOCV) methods. The nature of the Ru-CO bond is composite, i.e., electrostatic, covalent and charge transfer. Both donation and backdonation between CO ligand and Ru metal centre equally stabilize the Ru(II) complexes. Ru-CO photodissociation proceeds via a ^3^MC triplet excited state, exhibiting a conical intersection with the T_1_ ^3^MLCT excited state. Calculations show that these complexes show bands within visible while they are expected to be red emitters. Therefore, the [Ru(salen)(X)(CO)]^0/−1^ complexes under study could potentially be used for dual action, photoCORMs and theranostics compounds.

## 1. Introduction

Since its discovery in the 18th century, carbon monoxide (CO) was thought for a long time to be a deleterious gas [1,2,3,4,5,6,7,8,9,10]. Due to its odorless, colorless, and tasteless nature, CO has been characterized as a ‘*silent killer*’ [2]. However, CO could be turned from ‘*killer to healer*’, when administrated in a controlled manner and at doses not exceeding 12–14% of CO-bound hemoglobin (COHb) [6]. One way to deliver exogenous CO is by using the so-called CO-releasing molecules (CORMs) [11,12,13]. Up to now, the vast majority of CORMs are transition metal compounds having at least one CO ligand to be released upon administration. A very comprehensive review of metal-based CORMs has been recently published by Merlino et al. [14]. Releasing of CO from CORMs usually proceeds spontaneously upon dissolution in aqueous media, though in an uncontrollable manner [15]. The latter, however, could be alleviated, and controllable CO release could be achieved upon employing a stimulus such as an enzyme trigger [16], electromagnetic heating [17] or light [12,13,18]. The CORMs activated by light are termed photoCORMs and they usually release CO upon UV-Vis irradiation. The photoCORMs are mainly transition metal complexes, although there are a few organic photoCORMs reported in the literature [19,20].

The first examples of photoCORMs were the dimanganese decacarbonyl, Mn_2_(CO)_10,_ and iron pentacarbonyl, Fe(CO)_5_ complexes, which, upon irradiation, release CO [21]. Since then, quite a few efforts have been devoted to developing photoCORMs based on transition metal compounds [13,22,23,24]. Up to now, studies on photoCORMs have been focused mainly on Manganese, Iron, Rhenium, Ruthenium and Molybdenum complexes [13]. Amongst them, the most extensively studied are the Manganese complexes [25]. However, the main drawback of the latter is that they release CO upon UV irradiation, which is not optimal for tissue penetration. Mascharak et al. [15], with the aid of DFT computational methods, explored the possibility to red-shift the absorption of Mn complexes and, with a careful selection of ligands, e.g., replacing the tpm with tpa amine ligand, the absorbance of the [Mn(CO)_3_(tpa)]^+^ complex is red-shifted by up to 20 nm.

Complexes of other metals have attracted much less interest to date, as compared to Mn. Thus, a number of Fe complexes have been studied as photoCORMs, with the most notable being the iron–iron hydrogenase model compounds [26,27]. The main drawbacks of Fe photoCORMs are CO release with UV irradiation as well as low water solubility, which, upon suitable ligand modification, could probably be alleviated [12]. Studies on Re and Mo complexes acting as photoCORMs are even more scarce. Thus, Ford et al. reported the water-soluble photoCORM *fac*-[Re(bpy)(CO)_3_(thp)]^+^, which releases CO upon irradiation at 345 nm [28], while Smith et al. studied the antiproliferative potential of Re(I) tricarbonyl complexes bearing the aminoquinoline scaffold [29]. One Mo tricarbonyl complex was reported acting as photoCORM, namely the (Mo(C≡CCH_2_O-β-D-fructopyranose)(η^5^-C_5_H_5_)(CO)_3_) compound [30]. The latter is water soluble and releases CO upon irradiation at 325 nm. Finally, Ru complexes have attracted considerable interest in being used as photoCORMs. Mascharak et al. [13] studied the CO photo-release by a Ru complex resulting from modification of the [RuCl_2_(CO_3_)]_2_ (known as CORM-2) upon coordination of the qmtpm tridentate amine ligand. The [Ru(qmtpm)(CO)_2_Cl]^+^ complex exhibited an MLCT band at 405 nm, which, upon replacing CO with PPh_3_ ligand, was red-shifted, appearing at 465 nm. DFT computational studies were employed to delineate the σ-donation/π-accepting electronic effects of the ligands affecting the frontier orbitals being involved in the MLCT bands. Ru(II) carbonyl polypyridyl complexes were also studied as photoCORMs by Schatzschneider et al. [31]. These complexes, though thermally stable, absorb mainly in the UV region and exhibit small quantum yields.

In contrast to Ru(II) photoCORMs, there is a significantly higher number of studies on the respective NO photo-releasing Ru(II) complexes, namely photoNORMs, bearing a plethora of ligands, including macrocycles, carboxamides, polypyridines, porphyrins and ammines [32,33,34,35,36,37,38,39,40,41,42,43,44,45,46,47,48,49,50,51,52]. Amongst them, special attention has been devoted to Ru(II) photoNORMs bearing salen-type (N,N′-bis(salicylidene)ethylenediamine dianion) ligands. This is because salen-type ligands possess a versatile transition metal chemistry, are easy to synthesize at low cost and, are stable and robust while exhibiting structural and electronic tunability. Salens can coordinate via an N_2_O_2_ square-planar arrangement of their donor atoms around the metal centre at the equatorial position of octahedral Ru(II) complexes. They are appealing since they could form a synthetic platform where, upon suitable modification, they could easily immobilize NO and probably CO in Ru(II) complexes employed in photodynamic therapy (PDT).

Taking into account that the mechanism of action of photoCORMs is based on CO release upon irradiation, an in-depth study of the photodissociation mechanism of the M-CO bond is of great significance. Quantum chemical and electronic structure calculation methods are valuable tools for this purpose. Accordingly, based upon DFT calculations [53], it has been demonstrated that in d^6^ metal-carbonyl complexes like, for example, the Mn_2_(CO)_10_ complex, the high CO yield, upon Mn-CO photodissociation, is due to the involvement of dissociative high-lying LF states, exhibiting crossing with non-dissociative, lower MLCT states. The role of a dissociative ^3^MC excited state has also been revealed in a number of DFT studies of the ligand photodissociation mechanisms in various Ru(II) complexes [54,55,56,57], with the photo-detached ligands being acetonitrile, pyridine, butylamine, etc. In contrast, DFT studies on CO photodissociation from Ru complexes are scarce. In one such study, Vlĉek et al. [58] performed DFT/TDDFT calculations along with time-resolved IR spectroscopy to study the effect of the axial halide ligands on the photodissociation of the equatorial CO ligands upon irradiation of the *trans-(X,X)*-[Ru(X)_2_(CO)_2_(bpy)] (X = Cl, Br, I). For X = Cl^−^ or Br^−^, the CO photo-release occurs at the fs timescale, involving a ^1^LLCT/MLCT excited state, while for X = I^−^, Intersystem Crossing (ISC) leads to a population of a competing, “trapping” triplet excited state which is unreactive and limiting the CO release quantum yield. In a subsequent study, Vlĉek et al. [59] employed DFT/TDDFT calculations, along with time-resolved IR (TRIR) spectroscopy, to study the effect of the axial ligands, X and X′, on the Ru-CO photodissociation in the [Ru(X)(X′)(CO)_2_(*N*,*N*′-diisopropyl-1,4-diazabutadiene)] Ru(II) octahedral complexes. The CO photo-release occurs via a ^3^MLCT/XLCT excited state, and both DFT and TRIR showed a shift in the excited state’s *v*(CO) frequency with respect to the ground state with a magnitude strongly dependent on the nature of axial ligands.

In contrast to Ru complexes, there are quite a few theoretical studies on the M-CO photodissociation involving other transition metals. The photochemistry of first-row transition metal carbonyls has been the subject of numerous theoretical studies aiming at delineating the mechanism of the M-CO (M = V, Cr, Mn, or Ni) photodissociation [60]. It is now well established that the ultrafast CO photo-release from these metal carbonyl complexes proceeds via a ^1^MLCT excited state, being M-CO dissociative and avoiding crossings with higher-lying ^1^LF excited states, which were empirically thought of as being the M-CO dissociative states. Quite a few theoretical studies were also devoted to studying various metalloproteins, such as globins, including Hemoglobin (Hb) and Myoglobin (Mb). Thus, Head-Gordon et al. [61,62], employing DFT/TDDFT calculations, demonstrated that the ultrafast Fe-CO photodissociation occurs via two quasi-degenerate excited states populated upon crossing with the *Q* band (π → π*) excited states initially populated upon excitation of Heme. These findings were further corroborated by De Angelis et al. [63], who also studied the effect of the trans-axial ligands on the release of CO upon photodisociaiton of the Fe-CO bond.

Along these lines and taking into account all the above, we instigated to study a series of octahedral Ru(II) salen complexes of the general formula [Ru(salen)(X)(CO)]^0/−1^ (X = Cl^−^, F^−^, SCN^−^, DMSO, Phosphabenzene, Phosphole, TPH, CN^−^, N_3_^−^, NO_3_^−^, CNH^−^, NHC, P(OH)_3_, PF_3_, PH_3_) by means of DFT/TD-TDF electronic structure calculations. Our aims are: (i) to examine if the CO photo-release ability of these systems, upon Ru-CO bond photodissociation, could be probed just by using ground state ^13^C NMR spectroscopy measurements, (ii) to scrutinize the ground state bonding and electronic properties to examine how they affect the CO photo-releasing ability of these Ru(II) complexes under study, (iii) to delineate the mechanism of their CO photo-release upon irradiation and (iv) to study their photophysical properties with respect to their possible, simultaneous use as photoCORMs and theranostics. Until now, theoretical studies for Ru(II) complexes have mainly focused on exploring the Ru-CO photodissociation mechanism without examining the effect of the rest of the ligands in the coordination sphere. To the best of our knowledge, there is no systematic and in-depth theoretical study of the effects of *trans*-axial ligand on Ru-CO photodissociation in Ru(II) photoCORMs. Our study aims not only at delineating the Ru-CO photodissociation mechanism in these Ru(II) salen octahedral complexes as well as on how the *trans*-axial ligand would modulate CO release but also to explore the possibility of using ^13^C NMR to probe such mechanisms. Finally, the potential use of Ru(II) complexes simultaneously acting as photoCORMs and theranostics is also being examined based on the calculations of their photophysical properties.

## 2. Results and Discussion

**Scheme 1:**
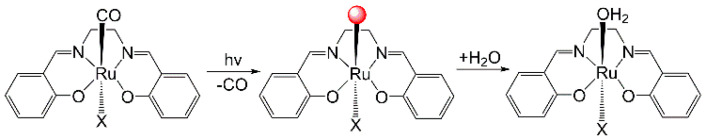
Photo-release of CO and formation of water Ru(II) complexes.

The Ru(II) complexes under study are expected to follow the generalized reactivity of a typical octahedral photoCORM [13] given in Scheme 1.

Upon irradiation, the Ru(II) octahedral complexes loose the CO ligand via photo-release, yielding a very reactive species of coordination number 5, exhbiting one vacant coordination site (marked with a red circle in Scheme 1). The coordinatively unsaturated pentacoordinate species could then react with a water molecule, which will occupy the axial vacant position, yielding an aqua Ru(II) octahedral complex.

### 2.1. Ground State Properties

#### 2.1.1. *S*_0_ Equilibrium Geometries

In the first step, the S_0_ ground state geometries of the carbonyl Ru(II) octahedral salen complexes, [Ru(salen)(X)(CO)]^0/−1^, were optimized employing DFT calculations at the PBE0/LanL2DZ(Ru)U6-31G(d,p)(E) level of theory in water solvent. Selected structural parameters are given in Table S1 of the Supporting Information Section. The calculated Ru-X bond lengths are within the range of 2.015–2.600 Å. On the other hand, the Ru-CO bond lengths are in the range of 1.820–1.908 Å, while the C-O bond lengths are in the range of 1.148–1.167 Å. Obviously, the bond length of the Ru-X bond depends strongly on the nature of the X ligand. The variation in the Ru-CO bond lengths is less pronounced compared to that observed for the Ru-X bonds. Nevertheless, the Ru-CO bond lengths reflect the *trans*-influence of the ligands X *trans* to the CO ligands in [Ru(salen)(X)(CO)]^0/−1^ complexes. Thus, based on the structural data, we could establish a *trans*-influence series for the Ru(II) complexes under study based on ligand X, which should be the following:

Cl^−^ < NO_3_^−^ < DMSO < SCN^−^ < N_3_^−^ < F^−^ < Phosphole < PH_3_ < Phosphabenzene < TPH < PF_3_ < CN^−^ < P(OH)_3_ < NHC < CNH^−^

It should also be noticed that the carbonyl ligand is linearly or almost linearly coordinated with the metal centre, as revealed by the calculated $\hat{\mathrm{Ru}\text{-}C\text{-}O}$ bond angles, lying in the range of 177.7–179.5°. Finally, the nature of the ligand X does not significantly affect the equatorial structural parameters pertaining to the salen ligand at the equatorial plane. Selected equatorial structural parameters such as the Ru-O and Ru-N coordination bond lengths and $\hat{O\text{-}\mathrm{Ru}\text{-}N}$ bond angles are given in Table S1 of the Supporting Information (SI). A perusal of Table S1 reveals that the nature of ligand X has a minor impact on the equatorial parameters. Thus, in all cases, the Ru-O and Ru-N bond lengths are found in the ranges of 2.091–2.123 Å and 2.011–2.029 Å, respectively, while the <O-Ru-N bond angles lie in the range of 91.5–92.4°.

Next, selected structural parameters calculated for the pentacoordinated intermediate, obtained upon CO photo-release (Scheme 1), are given in Table S2 of SI. As expected, due to the different nature of X, the Ru-X bond lengths vary significantly, found in the range of 1.797–2.392 Å. In contrast to the initial carbonyl complexes, the $\hat{\mathrm{Ru}\text{-}X\text{-}O}$ bond angles in the pentacoordinated intermediates deviate significantly from 90°, lying in the range of 87.0–99.2 Å. Finally, the equatorial parameters of the pentacoordinated intermediates do not vary significantly in the nature of ligand X (Table S2).

Table S3 also shows selected structural parameters of the [Ru(salen)(X)(H_2_O)]^0/−1^ aqua complexes, obtained upon coordination of a water ligand to the vacant site of the very reactive pentacoordinated intermediates. Inspection of Table S3 reveals that the Ru-X bond lengths in [Ru(salen)(X)(H_2_O)]^0/−1^ complexes vary significantly, being in the range of 1.830–2.469 Å, as expected since they depend strongly upon the nature of ligand X. In addition, the Ru-OH_2_ bond lengths are calculated to be in the range of 2.185–2.324 Å, strongly depending upon the nature of ligand X. Finally, the <Ru-N-OH_2_ bond angles deviate significantly from 90°, lying in the range of 87.9–93.3° (Table S3).

#### 2.1.2. Electronic and Bonding Properties

Let us now proceed in an in-depth analysis of the electronic and bonding properties of the [Ru(salen)(X)(CO)]^0/−1^ complexes to figure out the effects of the axial ligands X on their CO-releasing ability. The electronic properties of interest are basically the electronic effect of the axial ligands X on the *trans*–Ru-CO bond with respect to them in the Ru(II) complexes. We have already examined the *trans*-influence, also known as the structural *trans*-effect (STE), which is of electronic origin to the axial ligands X, based upon the calculated Ru-C bond lengths (vide supra).

Next, we would employ a method recently introduced by one of us [64] based on the concept of *trans*-philicity. *Trans*-philicity for a ligand L is an NMR-based parameter, defined as the strength of *philicity* of the coordination site in a *trans* position to itself, and it could potentially merge or even replace both the classic *trans*-effect (also known as the kinetic *trans*-effect, KTE) and *trans*-influence (STE) terms. *Trans*-philicity could be quantified upon employing a ^13^C NMR probe, in our case, via the following equation:

Δ*σ*^13^C = *σ*^13^C[Ru(salen)(X)(CO)]^0/−1^ − *σ*^13^C[Ru(salen)(CO)]^0/−1^ (1)

where Δ*σ*^13^C is the *trans*-philicity descriptor, *σ*^13^C[Ru(salen)(X)(CO)]^0/−1^ is the *σ*^13^C chemical shielding tensor of CO ligand in the [Ru(salen)(X)(CO)]^0/−1^ complexes and *σ*^13^C[Ru(salen)(CO)]^0/−1^ is the *σ*^13^C chemical shielding tensor of CO ligand in a reference [Ru(salen)(CO)]^0/−1^ complex (obtained upon discarding ligand X). A large Δ*σ*^13^C parameter for a ligand indicates high *trans*-philicity. Table 1 shows the Δ*σ*^13^C parameter, calculated for the [Ru(salen)(X)(CO)]^0/−1^ complexes at the PBE0/LanL2DZ(Ru)U6-31G(d,p)(E) level of theory in water solvent. In order to validate our results, we set out to calculate the chemical shifts, *δ*^13^C NMR of the CO ligand versus the TMS reference for complexes **1**–**15**, employing the same level of theory used, namely the PBE0/LanL2DZ(Ru)U6-31G(d,p)(E) in water solvent. The chemical shifts *δ*^13^C vs. TMS were calculated based on the following formula:

*δ*^13^C = *σ*^ref^(^13^C) − *σ*(^13^C) (2)

where *σ*^ref^(^13^C) is the ^13^C chemical shielding tensor for the TMS reference compound, which is found to be 196.9 ppm at the PBE0/LanL2DZ(Ru)U6-31G(d,p)(E) level in water solvent. *σ*(^13^C) is the ^13^C chemical shielding tensors calculated for the CO ligand of complexes **1**–**15** at the same level of theory. The *δ*^13^C extracted using Equation (2) is found to lie in the range of 215–238 ppm, being in excellent agreement with experimentally derived *δ*^13^C chemical shifts for the CO ligands of various Ru complexes, found within the range of 178–250 ppm (vs. TMS) [65,66,67,68]. Table S4 shows the *σ*^13^C chemical shielding tensors and the *δ*^13^C NMR of the CO ligand versus the TMS reference.

**Figure 1:**
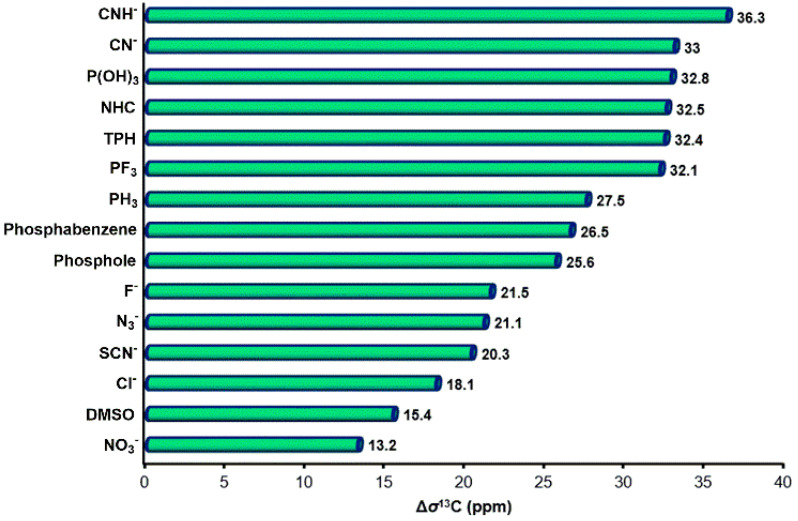
*Trans*-philicity ladder for the [Ru(salen)(X)(CO)]^0/−1^ complexes.

**Figure 2:**
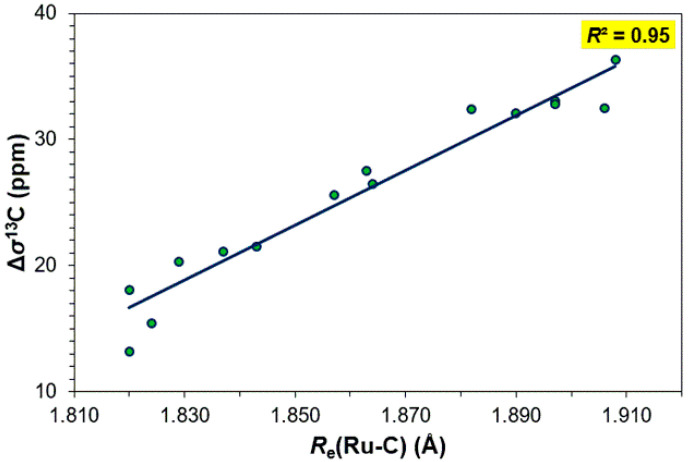
Linear correlation of Δ*σ*^13^C versus *R*_e_(Ru-C) of [Ru(salen)(X)(CO)]^0/−1^ complexes.

Inspection of Table 1 reveals that the highest *trans*-philicity is exhibited by the CNH^−^ ligand of complex **11,** while the lowest one is exhibited by the NO_3_^−^ ligand of complex **10**. Accordingly, we could construct a *trans*-philicity ladder for the [Ru(salen)(X)(CO)]^0/−1^ complexes under investigation, as depicted in Figure 1. Remarkably, an excellent linear correlation could be observed between Ru-C bond length and the *trans*-philicity parameter, as can be seen in Figure 1. The validity of the *trans*-philicity concept as a powerful tool for quantifying the *trans* ligand effects, merging *trans*-effect and *trans*-influence, has been recently demonstrated for octahedral [Cr(CO)_5_L]^−/0/+^ complexes [64] as well for square planar Pt(II) complexes [69,70]. Accordingly, based on the linear correlation given in Figure 2, it is obvious that the *trans*-philicity concept could also be applied to octahedral Ru(II) complexes since it reproduces very well the STE, quantified by the Ru-C bond length. The linear relationship of Δ*σ*^13^C vs. *R*_e_(Ru-C), given in Figure 2, indicates that the ligands exhibiting the strongest STE, aka *trans*-influence, should exhibit the highest *trans*-philicity. The following increasing order for ligands X could be constructed, based on the Δ*σ*^13^C parameter:

$${NO_{3}}^{-}<\mathrm{DMSO}<{\mathrm{Cl}}^{-}<{\mathrm{SCN}}^{-}<{N_{3}}^{-}<F^{-}<\mathrm{Phosphole}<\mathrm{Phosphabenzene}<\;{\mathrm{PH}}_{3}<{\mathrm{PF}}_{3}<\mathrm{TPH}<\mathrm{NHC}<P{\left(OH\right)}_{3}<{\mathrm{CN}}^{-}<{\mathrm{CNH}}^{-}$$

Although the above series presents some differences compared to that obtained based on the *R*_e_(Ru-CO) structural parameter, it basically exhibits the same essence. Thus, ligands like P(OH)_3_, CN^−,^ and CNH^−^ exert strong effects on the Ru-CO bond, while ligands like NO_3_^−^, DMSO, and Cl^−^ exert the weakest effects.

To further quantify the validity of the Δ*σ*^13^C *trans*-philicity parameter to quantify the *trans* ligands effects, we set out to compare it with other, already established, parameters that have been employed for the same purpose.

One such parameter is the so-called metal–ligand electronic parameter (MLEP), introduced by Cremer and Kraka [71], to describe the *trans*-effect of the ligands based upon the local stretching vibrational frequency of the M-L bond being in *trans*-position with respect to them. Table 1 shows the MLEP parameters calculated for the [Ru(salen)(X)(CO)]^0/−1^ complexes, corresponding to the Ru-C bond stretching vibrational frequency *v*_s_(Ru-C). The very good linear correlation observed between the MLEP and the *trans*-philicity parameter (see Figure S1 of SI) signifies the validity of the latter to correctly describe *trans*-effects. On the other hand, the *v*_s_(C-O) stretching vibrational frequency gave a poor correlation with *trans*-philicity. Nevertheless, the computed *v*_s_(C-O), being within the range of 1996–2111 cm^−1^, confirms the validity of our computational method in predicting IR stretching vibrations since the experimentally derived *v*_s_(C-O) usually lies within the range of 1900–2000 cm^−1^ [66,67].

Another parameter proposed as a measure of *trans*-effects is the intrinsic Bond Dissociation Energy, iBDE corresponding to the intrinsic strength of a bond [72]. An excellent linear correlation could be observed between the *trans*-philicity parameter Δ*σ*^13^C and iBDE (see Figure S2 of SI). This further corroborates the validity of the *trans*-philicity, Δ*σ*^13^C, since it is in line with the iBDE energetic *trans*-effect parameter.

Next, we set out to scrutinize the bonding properties of the Ru-CO bond and delineate how it may be involved or modulate the CO photo-release process.

**Figure 3:**
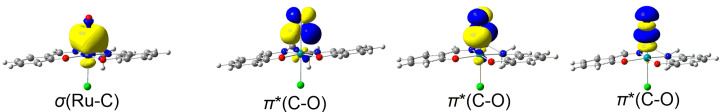
3D isosurfaces of most relevant NBOs calculated for complex **1**.

At first, the bonding properties of the [Ru(salen)(X)(CO)]^0/−1^ complexes were investigated by employing the Natural Bond Orbitals, NBO method [73]. The most relevant NBOs, located on the Ru-CO and C-O bonds, are depicted schematically in Figure 3. In terms of NBOs, the Ru-CO bond could be described by four NBOs, i.e., one *σ* bonding, *σ*(Ru-CO), and three antibonding π*. For representative complex **1**, the *σ*(Ru-CO) NBO is constructed by the linear combination 0.565*h*_Ru_ + 0.825*h*_C_. The *h*_Ru_ and *h*_C_ are hybrid orbitals of spd-type (sp^1.44^d^1.41^) and sp-type (sp^0.56^), respectively. The occupation number of *σ*(Ru-CO) is 1.837. The linear combinations, hybrid orbitals, and occupation numbers calculated for the rest of the complexes under study, **2**–**15**, are given in Table S5 of SI.

Next, the three π* NBOs of the CO ligand are located mainly on the C donor atom. Table 2 also gives selected NBO parameters calculated for the Ru(II) complexes under study.

Examination of Table 2 reveals that in all cases, the Ru(II) metal centre departs from its formal +2 oxidation state, acquiring a negative natural charge, indicating the acceptance of electron density from the surrounding ligands. On the other hand, the positive natural charge on the C atom of the CO ligand increases with respect to that calculated for the ‘free’ CO molecule (0.510) as a result of electron donation to the Ru metal centre.

Nevertheless, the opposite charges calculated for the Ru metal centre and the C atom of the CO ligand are indicative of the electrostatic/charge transfer components of the Ru-CO bond. The third component of the Ru-CO bond, namely the covalent bond, is clearly reflected in the WBI bond order values, being in the range of 1.095–1.363, corresponding roughly to a single covalent bond. In contrast, the WBI bond order values of the C-O bond in the CO ligand are found in the range of 1.999–2.160, which is significantly reduced with respect to that found for the ‘free’ CO molecule being equal to 3. Thus, upon coordination of CO to the Ru(II) metal centre, the triple C≡O bond is transformed into a double-like bond as a result of the electron density donation towards the metal. Finally, there is a change in the natural electron configuration, *nec* of the C atom upon coordination of the CO ligand to the Ru metal centre. Consequently, while the *nec* of the C atom is calculated to be 2s^1.66^2p^1.79^ in the ‘free’ CO molecule upon coordination with the Ru metal centre, we observe a reduction in the ranges of 0.46–0.62 and 0.23–0.33 |e| of the 2s and 2p populations, respectively. In contrast, only minor changes in the *nec* of the O atom could be observed upon coordination of CO to Ru. Based on *nec*, it is obvious that the Ru-CO metal–ligand interactions in the Ru(II) complexes under study affect only the C donor atom and the O oxygen atom of the CO ligand. This could rationalized by the fact that donation from CO to Ru metal centre occurs from the orbital location, almost entirely on the C donor atom (see Figure 3, *σ*(Ru-C)), while in Ru to CO backdonation, the π*(C-O) orbitals are mainly located on the C atom.

Remarkably, the WBI of the Ru-CO bond correlates very well with *trans*-philicity*,* i.e., the higher the Δ*σ*^13^C, the lower the WBI of the Ru-CO bond. Figure S3 depicts the excellent linear correlation of Δ*σ*^13^C versus WBI (*R*^2^ = 0.93), indicating that the ligands X with high Δ*σ*^13^C values weaken the *trans*-Ru-CO bonds, consequently exhibiting lower WBIs.

In addition, within the framework of the NBO method, we performed the Natural Energy Decomposition Analysis, NEDA method [74,75,76,77]. The results of NEDA for the Ru-CO bond in the [Ru(salen)(CO)X]^0/−1^ complexes in the S_0_ state are tabulated in Table 3. Within the framework of NEDA, the total interaction energy, Δ*E*, between two fragments constituting a bond is given by the following equation:

Δ*E* = *EL* + *CT* + *CORE* (3)

where *EL* is the electric term accounting for electrostatic and polarization interactions, *CT* is the charge transfer term, and *CORE* represents the intermolecular exchange and deformation interactions. Upon examining Table 3, we observe that Δ*E* values are in the range of −33 to −61 kcal/mol, in line with the calculated IBDE values (Table 1). The values of the *EL* term tabulated in Table 3 indicate a significant contribution of electrostatic and polarization interactions in the Ru-CO bond formation. Conversely, the *CT* term values are more than twice as high as the respective *EL* term values. Obviously, the charge transfer interactions dominate the total Ru-CO bonding interactions. The destabilizing core repulsion interactions are very high, as reflected by the values of the *CORE* term given in Table 3, though they are fully compensated by the stabilizing total bonding interactions given by the sum of the *EL* and *CT* terms. Notice that Δ*Ε* correlates very well with *trans*-philicity*,* and the same also holds for the three components, *EL*, *CT,* and *CORE*. This is corroborated by the very good linear correlations between Δ*σ*^13^C versus Δ*Ε* (*R*^2^ = 0.96), *EL* (*R*^2^ = 0.81), *CT* (*R*^2^ = 0.93), and *CORE* (*R*^2^ = 0.87) depicted in Figure S4 in the SI. These correlations further demonstrate the power of the *trans*-philicity concept to capture the effect of the *trans*-ligands on the various components of the Ru-CO bond.

Finally, in order to scrutinize the bonding situation of the Ru-CO bond, we also calculated the Natural Orbitals for Chemical Valence, NOCVs, which were proven very powerful in describing metal–ligand interactions, e.g., the *σ* donation/*π* backdonation in the heme-CO complex [78].

**Figure 4:**
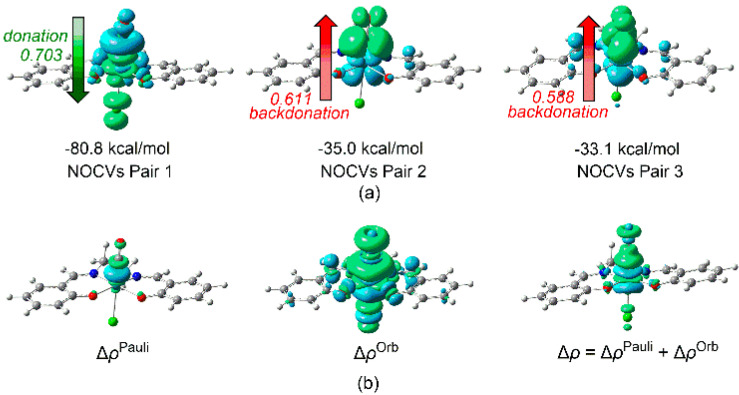
(**a**) 3D isosurfaces of selected NOCV pairs, energies, and eigenvalues (in italics) (**b**) 3D representation of Δ*ρ*^Pauli^, Δ*ρ*^Orb^, and Δ*ρ* for complex **1** (blue and green isosurfaces display decreased and increased electron density regions, respectively).

Figure 4 shows the three NOCV pairs for the representative complex **1** with the highest impact on the density difference due to the orbital interactions. Notice that the sum of the energies of these three NOCV pairs is about 149 kcal/mol, accounting for 95% of the total orbital interactions, Δ*E*^orb^ totaling 157 kcal/mol. Inspection of Figure 4 shows that the three NOCV pairs depicted in Figure 4 are related to the Ru-CO bond in the **1**, while the same is also observed for the rest of the complexes **2**–**15**.

From the shape of NOCVs Pair 1 (Figure 4), as well as its eigenvalue being equal to 0.703, it is clear that there is a donation of electron density from CO towards the Ru(II) metal centre and the Cl^−^ ligand. On the other hand, the shape of NOCVs Pairs 2 and 3, as well as their eigenvalues, being 0.611 and 0.588, respectively, reflect the Ru(II) → CO backdonation, often observed in transition metal complexes bearing π-acid ligands like CO.

The eigenvalue-weighted orbital density of NOCVs Pair 1 is given by the linear combinations Δ*ρ*_pair1_(r) = 0.703[*φ*_1_(r)]^2^ − 0.703[*φ*_418_(r)]^2^. The 3D isosurfaces of *φ*_1_(r) and *φ*_418_(r) (Figure S5) reveal that the former is an antibonding combination of *s* and *d*_z2_ Ru orbitals with *p*_z_-type orbitals of Cl^−^ and CO ligands while the latter is a bonding combination of *s* and *d*_z2_ Ru orbitals with mainly s- and p_z_-type orbitals of CO ligand. Thus, the CO → Ru donation corresponds to electron density transfer from the antibonding *φ*_1_(r) NOCV towards the bonding *φ*_418_(r) NOCV, resulting in stabilization of the system, amounting to −81 kcal/mol (Figure 4).

Conversely, the eigenvalue-weighted orbital densities of NOCVs Pairs 2 and 3 are given by the linear combinations Δ*ρ*_pair2_(r) = 0.611[*φ*_2_(r)]^2^ − 0.611 [*φ*_417_(r)]^2^ and Δ*ρ*_pair3_(r) = 0.588[*φ*_3_(r)]^2^ − 0. 588[*φ*_416_(r)]^2^, respectively. Both *φ*_2_ and *φ*_3_ are bonding combinations of *d* AOs of Ru(II) (*d*_xz_- and *d*_yz_-type) with π orbitals of CO (*p*-type), while *φ*_417_ and *φ*_416_ are antibonding combinations of the same π-type orbitals localized on Ru and CO (Figure S5). Therefore, the Ru → CO backdonation corresponds to electron density transfer from the bonding *φ*_2_(r)/*φ*_3_(r) towards antibonding *φ*_417_(r)/*φ*_416_(r) NOCVs. Even so, the Ru → CO backdonation results in the stabilization of the system totaling −67 kcal/mol (Figure 4, −35 plus −33 kcal/mol).

Table S6 gives the energies, eigenvalues, linear combinations, and % compositions of the dominant NOCV pairs for the rest of the complexes **2**–**15**. In all cases, we can observe the same phenomena as for **1**, i.e., CO → Ru donation due to electron density transfer from antibonding to bonding *φ* NOCVs, as well as Ru → CO backdonation from bonding to antibonding *φ* NOCVs. The stabilization of **2**–**15** due to the CO → Ru donation is found in the range of −79 to −28 kcal/mol, while the Ru → CO backdonation stabilization is within the range of −77–45 kcal/mol.

Figure 4b shows the maps of Pauli deformation density (Δ*ρ*^Pauli^), orbital deformation density (Δ*ρ*^orb^), and total deformation density (Δ*ρ*). Perusal of Figure 4b indicates that Pauli repulsion, Δ*ρ*^Pauli^, causes decrease in electron density in the region between Ru(II) and CO. Contrarily, orbital interaction, Δ*ρ*^orb^ signifies electron density accumulation in the region between Ru(II) and CO. Overall, based upon the shape of Δ*ρ*, we observe that from the inter-fragment interaction emerges an increase in electron density in the region between Ru(II) and CO.

### 2.2. Mechanism of CO Photodissociation

It is generally accepted that bond photodissociation in Ru(II) complexes begins upon excitation to the first singlet excited state of MLCT character, ^1^MLCT, which, via Intersystem Crossing (ISC), yields a triplet excited state of MLCT character, ^3^MLCT [79,80,81,82,83]. The latter could then undergo the transition to another, low-lying triplet excited state, which is of metal-centreed character, ^3^MC. The ^3^MLCT to ^3^MC transition could occur by the population of the latter upon radiative (photon emission) or non-radiative (thermal) deactivation of the former state. The ^3^MC state is dissociative with respect to the bond to be cleaved, i.e., their SOMOs are located on this bond, being antibonding, dσ*-type MOs [53,54,55,56,57,58,59,60,61,62,63,64,65,66,67,68,69,70,71,72,73,74,75,76,77,78,79,80,81].

**Figure 5:**
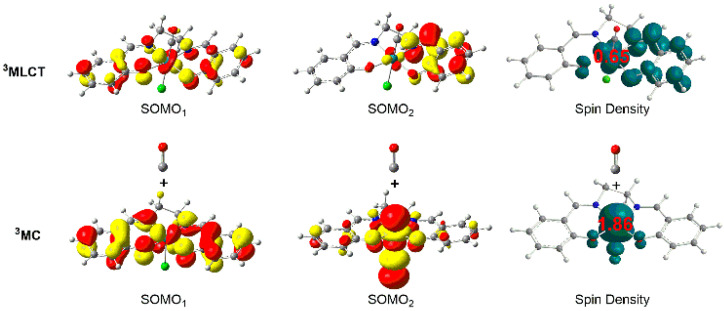
3D isosurfaces of SOMOs of **1** in ^3^MLCT and ^3^MC states, along with Mulliken spin densities (in red font) on Ru metal centre calculated at the PBE0/LanL2DZ(Ru)U6-31G(d,p)(E) level of theory in water solvent.

Accordingly, we set out to explore the potential energy surface (PES) of the triplet excited states of the Ru(II) complexes under study in order to delineate the Ru-CO bond photodissociation in these systems. The Singly Occupied Molecular Orbitals, SOMOs, along with the spin densities on the Ru metal centres calculated for the representative complex **1**, are given in Figure 5. Inspection of Figure 5 reveals that both SOMO_1_ and SOMO_2_ in the ^3^MLCT state are antibonding dπ* MOs, localized mainly on the metal centre as well as on the salen ligand. On the other hand, in the ^3^MC state, although SOMO_1_ is an antibonding dπ* MO, localized on the metal centre as well as on the salen ligand, SOMO_2_ is an antibonding dσ* MO, localized on the metal centre and the ligand X being dissociative with respect to the Ru-CO bond (vide infra).

**Figure 6:**
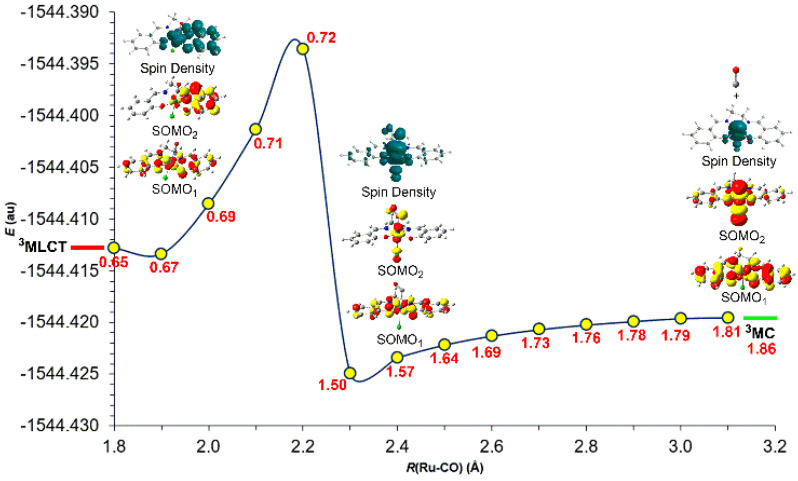
Relaxed PES scan of the triplet states of **1** yielded upon stretching the Ru-CO bond to dissociation along with the respective 3D surfaces of the SOMO_1_, SOMO_2,_ and Spin Density evolution (numbers in red are Mulliken Spin Densities).

The triplet PES scan of **1** upon stretching the Ru-CO bond from the ^3^MLCT state to the final, dissociative ^3^MC state is depicted in Figure 6. Analogous triplet state scan curves were also obtained for the rest of the complexes under study, **2**–**15**. From Figure 6, it is evident that upon elongating the Ru-CO bond, the dπ* SOMO_1_ retains its character from the initial ^3^MLCT state all the way to the final ^3^MC dissociative state. However, there is a minor change in SOMO_1_ character, observed for *R*(Ru-CO) ≥ 2.3 Å, where a sudden drop in energy occurs. Accordingly, for *R*(Ru-CO) ≥ 2.3 Å, the SOMO_1_ is still of dπ* character, though with a d_yz_ orbital in place of d_z2_ AO observed for *R*(Ru-CO) < 2.3 Å. Apparently, the Ru-CO bond dissociation is more closely related to the evolution of the SOMO_2_. Remarkably, SOMO_2_ changes from dπ* to dσ* character for *R*(Ru-CO) ≥ 2.3 Å. Thus, the dσ* antibonding SOMO_2_ promotes the Ru-CO bond dissociation for distances equal to or longer than 2.3 Å where the ^3^MC state starts being populated. This is further corroborated by the evolution of Mulliken Spin Densities during the Ru-CO bond stretching depicted in Figure 6. Inspection of Figure 6 reveals that there is a continuous increase in the Mulliken Spin Density on the metal centre upon stretching the Ru-CO bond. The increase in the Mulliken Spin Density of the metal centre is maximum upon stretching the Ru-CO bond from 2.2 to 2.3 Å, and this reflects the ^3^MLCT to ^3^MC transition. For Ru-CO distances greater than 2.3 Å, the Mulliken Spin Density does not vary significantly, reaching a limit of 1.85 for the products of Ru-CO photodissociation, namely the ^3^MC pentacoordinated complex and CO.

**Figure 7:**
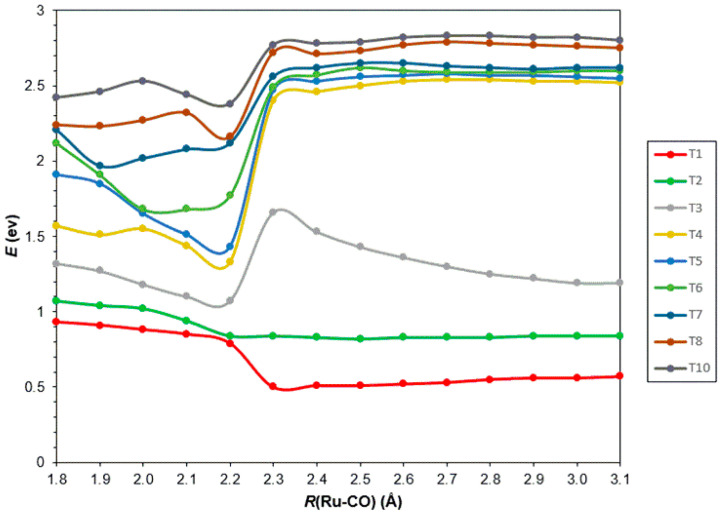
PES scan of the manifold of the 10 lowest triplet excited states of **1** in S_0_ state, along the Ru-CO bond stretch calculated at the TD-DFT/PBE0/LanL2DZ(Ru)U6-31G(d,p)(E) level of theory in water solvent.

It is expected that the ^3^MLCT to ^3^MC transition will occur via a conical intersection between these two triplet excited states. A perusal of Figure 6 (vide supra) reveals that the first triplet excited state T_1_ is of ^3^MLCT character, in line with evidence from previous studies on Ru(II) complexes [56,57]. Therefore, it is of interest to locate the higher-energy ^3^MC triplet excited state leading to the Ru-CO bond dissociation via a conical intersection with the T_1_ ^3^MLCT state. Accordingly, we have calculated the potential energy curves for the manifold of the ten lowest triplet excited states upon stretching the Ru-CO bond in the optimized S_0_ state of the Ru(II) complexes under study by an increment of 0.2 Å in a range from 1.8 to 3.2 Å followed by TDDFT calculations on each stretching step (Figure 7).

From Figure 7, it is obvious that there should be a conical intersection between the T_1_ and T_2_ states at a Ru-CO distance equal to 2.2 Å. This is in accordance with the curve obtained from the Relaxed PES scan of **1** upon stretching the Ru-CO bond, which exhibits a maximum at the same distance, i.e., at 2.2 Å (see Figure 7).

**Figure 8:**
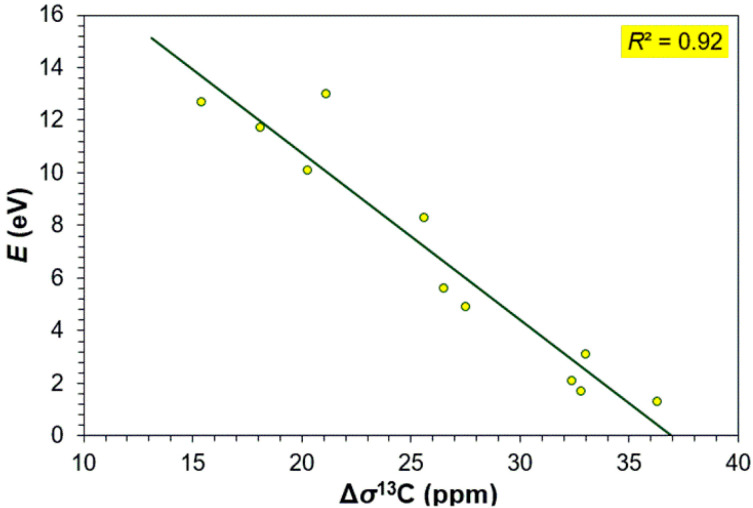
Linear correlation between the energy barrier, *E* for Ru-CO photodissociation versus the Δ*σ*^13^C parameter.

The magnitude of the T_1_–T_2_ conical intersection amounts to only ~0.2 eV (or ~5 kcal/mol). This marginal energetic difference should permit an easy T_1_ to T_2_ ‘jump’, leading to the Ru-CO dissociation. Analogous PES scans for the manifold of the ten lowest triplet excited states were also calculated for the rest of the Ru(II) complexes under study (see Figures S6–S9). The magnitude of the conical intersections between the T_1_ and the dissociative triplet excited states of higher energies in complexes **2**–**15** lies in the range of 0.01–0.05 eV, while the energy barrier for conversion of the ^3^MLCT T_1_ state to the ^3^MC T_2_, Ru-CO dissociative triplet state (see also Figure 6) lies in the range of 1.3–12.7 eV at the PBE0/LanL2DZ(Ru)U6-31G(d,p)(E) level. Notably, there is an excellent linear correlation between this energy barrier and the *trans*-philicity parameter, Δ*σ*^13^C (*R*^2^ = 0.92), given in Figure 8 (excluding complexes **2**, **10**, **12,** and **14** being outliers).

A very good linear correlation (*R*^2^ = 0.84) is observed for the conical intersection energy gap and the *trans*-philicity parameter (Figure S10). Thus, the Ru(II) complexes with ligands of strong *trans*-philicity are expected to release CO more facile. This finding holds promise for probing the CO photo-release from transition metal complexes with the use of ground-state ^13^C NMR techniques.

### 2.3. Photophysical Properties

**Figure 9:**
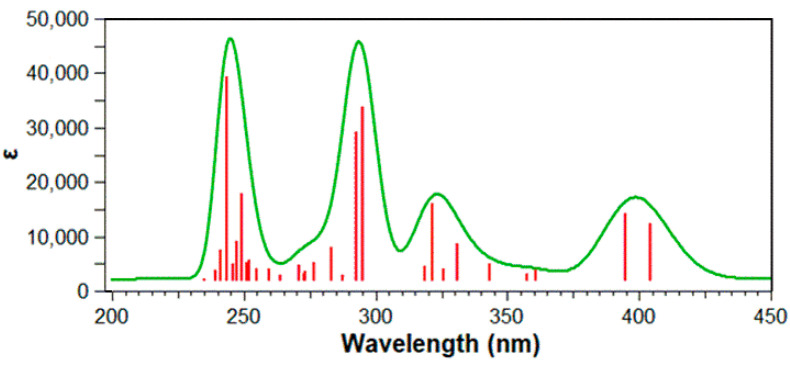
Simulated UV-Vis absorption spectrum of **1** (FWHM = 0.1) calculated at the TDDFT/PBE0/LanL2DZ(Ru)U6-31G(d,p)(E) level of theory in water solvent.

In order to explore the possibility of the Ru(II) complexes under study being used for PDT, we have simulated their absorption spectra. Figure 9 depicts the simulated absorption spectrum for the representative complex **1**. The simulated absorption spectra of the rest of the complexes under study are given in Figures S11–S13 of SI. The simulated absorption spectrum of **1** shows four bands in the region of 225–425 nm. Similar bands are also observed for the rest of the Ru(II) complexes under study (Figures S11–S13). Let us analyze the bands calculated for **1** (analogous should be the analysis for the rest of the complexes). There are two intense, high-energy bands, peaking around 245 and 295 nm, while in addition, there are also two less intense bands of lower energy, peaking around 320 and 400 nm.

The two high-energy bands arise from a multitude of electronic transitions, and the same holds true for one out of the two low-energy bands. In contrast, the lowest-energy band arises only from two electronic transitions.

Table 4 shows the electronic excitations pertaining to the most intense electronic transitions comprising the four bands observed in the simulated absorption spectrum of **1**. The lowest-energy band, around 400 nm, comprises two electronic transitions at 404 and 395 nm. The former is due to a HOMO to LUMO excitation, while the latter is due to a HOMO to LUMO+1 excitation (Table 4). The HOMO is a dπ* ΜO while the LUMO and LUMO+1 are π* ΜOs (see Figure S14 in SI). Thus, the electronic transitions comprising the band around 400 nm could be assigned as MLCT/IL. The next low-energy band, around 320 nm, comprises five electronic transitions, with the most intense ones peaking at 322 and 331 nm. Both of these electronic transitions arise from excitations between HOMO-1, LUMO, and LUMO+1 and could, therefore, be assigned as MLCT/IL. The two high-energy bands around 245 and 295 nm arise both from a multitude of electronic transitions. The two principal electronic transitions, included in the band around 295 nm, appear at 293 and 295 nm (Figure 9). The former arises from a HOMO-3 to LUMO excitation, while the latter is a combination of excitations involving the same MOs as well as the LUMO+1 and LUMO+2. A perusal of Figure S11 in SI reveals that HOMO-3 is mainly located on the metal, while the unoccupied MOs are located on the ligands (vide supra), with the exception of LUMO+2, which is located on the metal.

Thus, the band around 295 nm could be assigned as MLCT/IL/MC. Finally, the two principal electronic transitions, giving rise to the highest-energy band, appear at 243 and 249 nm. These electronic transitions arise from combinations of excitations involving many MOs, i.e., HOMO, HOMO-1,3,5,6, LUMO, and LUMO+1,3,6. From the shapes of these MOs (Figure S11), we could characterize the two electronic transitions and, subsequently, the band around 245 nm as MLCT/IL/MC (IL = intra/inter ligand). Table 4 also shows the transition dipole moments, *TDM*s, for the respective electronic transitions under examination. The relatively high values of *TDM*s calculated for these electronic excitations indicate that they are allowed under the electric dipole interaction. The highest *TDM* values are observed for the excitations of the electronic transitions at 243 and 295 nm, while the lowest one is for 331 nm. Finally, Table 4 shows the % character of charge transfer between the metal and the ligand, %*CT*, which is a quantitative measure of the metal character of an electronic transition. The %*CT* is calculated using the following Equation [84]:

(4) $$\%CT=\;\sum_{i,j}{[C(i\to j)]}^{2}(\%M_{i}-\%M_{j})$$

where *C*(*i* → *j*) are the coefficients of each of the *i* → *j* excitations from state *i* to state *j*. %M_i_ and %M_j_ are the % metal orbital contributions to the orbitals involved in the excitations. Positive %*CT* values specify a net MLCT contribution, while negative %*CT* values are the opposite, i.e., a net LMCT contribution. A perusal of Table 4 reveals that the strongest MLCT character is observed for the electronic transition at 295 nm (11%), while %CT is quite high for the electronic transitions at 293, 395, and 404 nm (10%). For the electronic transition at 249 nm, we obtain a negative %*CT* value equal to −13%, meaning an LMCT character.

**Figure 10:**
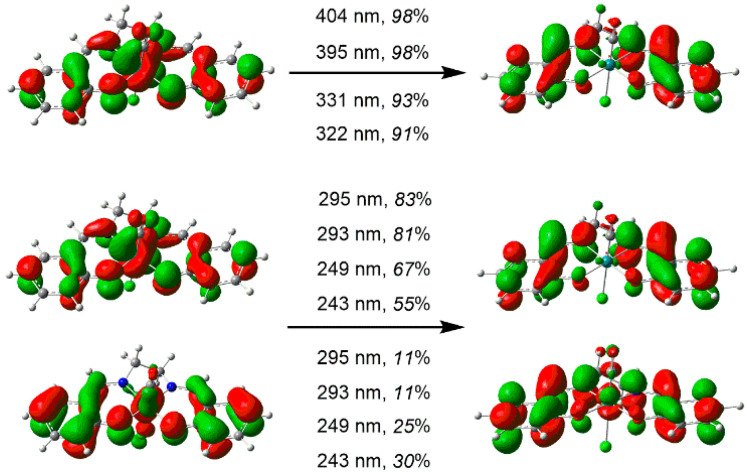
NTO pairs for relevant electronic transitions appearing in the absorption spectrum of **1** (hole, *h*, on the **left**; particle, *e*, on the **right**).

Finally, in order to simplify the description of the electronic transitions (especially those arising from combinations of excitations, making their assignment perplexing), we have calculated the Natural Transition Orbitals, NTOs employing the method of Martin [85]. Figure 10 depicts the NTO pairs that are relevant to the electronic excitations given in Table 4.

Evidently, the electronic transitions are described by a few NTO pairs. Thus, the electronic transitions at 404, 395, 331, and 322 nm are described by an NTO pair where the hole is mainly located on the metal and, to a lesser degree, on the salen ligand, while the particle is solely located on the salen ligand. Therefore, the group of low-energy electronic transitions and, subsequently, the respective bands could be assigned as MLCT/IL in accordance with the MO-based analysis (vide supra). On the other hand, the electronic transitions concerning the group of high-energy bands, i.e., at 295, 293, 249, and 243 nm, are described by two NTO pairs. Among them, the major contribution comes from the pair, which is the same as that found for the low-energy electronic transitions. The other NTO pair, describing the high-energy electronic transition, has a minor contribution to the excitation, and in this case, both the hole and the particle are delocalized on the metal and salen ligand as well. Thus, based on the NTO pairs, the group of high-energy electronic transitions/bands is assigned as MLCT/IL. Accordingly, the vertical emission energy, *λ*_ver_, corresponds to the transition from point **b** (optimized geometry of T_1_ state) towards point **c** (the Franck–Condon state). Next, the vibrational reorganization energy, *λ*_vib_, is calculated for the transition from point c back to the ground S_0_ state minimum. Finally, we estimated both the zero–zero transition, *E*_0–0_, and the energy difference between the ground state S_0_ and the emissive first triplet excited state T_1_. Table 5 gives the calculated values of all the above-mentioned parameters for the Ru(II) complexes under investigation. An inspection of Table 5 reveals that most of the Ru(II) complexes under study emit in the NIR.

**Scheme 2:**
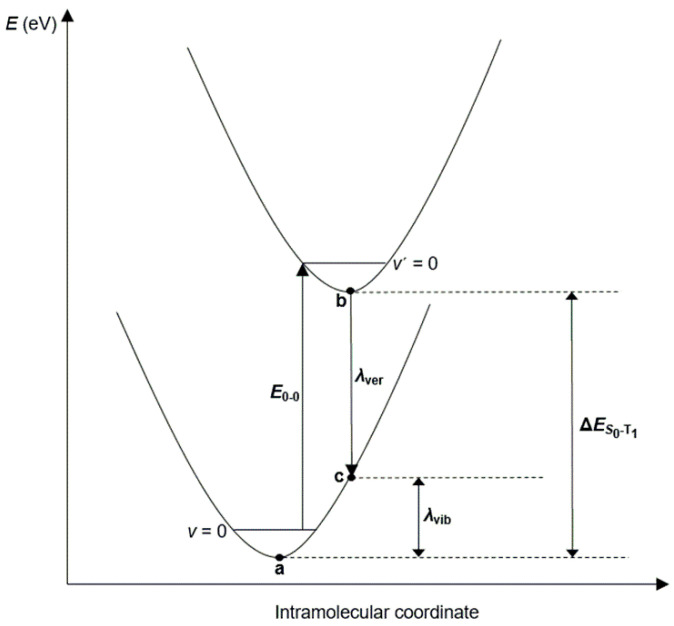
Photophysical parameters relevant to the emission spectra.

Next, we set out to examine the potential use of the Ru(II) complexes under study as theranostics coupled with their action as photoCORMs. Therefore, we have calculated their emission spectra, which could give us a hint at their potential as theranostics based on their luminescence. The photophysical parameters relevant to the emission properties were calculated based on the diagram depicted in Scheme 2.

In general, the calculated emission maxima, *λ*_max_ of **1**–**15**, are predicted to be within or near the so-called ‘body window’ (700–900 nm), rendering them advantageous since, in these wavelengths, the penetration of the tissues is easier. All the complexes are expected to be red emitters. Exceptions are complexes **5** and **15**, which are infrared emitters that are even more efficient for tissue penetration. The emission maxima, *λ*_max_ exhibit a red-shift trend with respect to the nature of the only variable in these Ru(II) complexes under study being the axial ligands X. Thus, qualitatively, the red-shifting of *λ*_max_ tends to be larger for the ligands X exhibiting weaker *trans*-effects. Therefore, within this respect, the Ru(II) complexes under study are expected to be the most suitable as theranostics since red/NIR emissions penetrate human tissues more easily. On the other hand, the zero–zero transition, *E*_0–0_, is estimated to be within the range of 35–56 kcal/mol. Thus, the peak due to zero–zero transition will be located around green and up to red/NIR, which would further enhance the suitability of these complexes as theranostics. Values of *E*_0–0_ in this range are usually indicative of a triplet MLCT state. Their relatively large magnitude indicates complexes with rigid structures undergoing minimal structural changes upon S_0_–T_1_ excitation, as well as a good overlap between those two states. Thus, based on these arguments, the Ru(II) systems under study are expected to exhibit intense phosphorescence emissions. In this respect, complexes **7**, **8,** and **11** should give the most intense emissions. The same holds true for the quantum yields for these complexes, which should be large due to the fact that high *E*_0–0_ values imply small Stokes shifts and minimal non-radiative deactivation.

Next, *λ*_vib_ values are found in the range of 8–16 kcal/mol, with the exceptions of complexes **5** and **15** being outside this range with values equal to 26 and 31 kcal/mol, respectively (Table 5). The relatively small *λ*_vib_ exhibited by the majority of the Ru(II) complexes under study indicates that the relaxation to the S_0_ ground state from the Franck–Condon state undergoes small structural changes corresponding to small Stokes shifts. In addition, small *λ*_vib_ implies higher quantum yields, minimizing non-radiative deactivation (vibrational relaxation). Based upon *λ*_vib,_ complexes **2**, **7**, **8**, **11**, and **13** are the most favorable. Finally, the energy difference between S_0_ and T_1_, Δ*E*_S0–T1_, is relatively high, being in the range of 47–57 kcal/mol (exceptions are again **5** and **15**), signifying that these Ru(II) complexes should be efficient phosphorescence emitters based on the energy gap law [86,87].

## 3. Computational Methods

All calculations were performed with the Gaussian16W, Rev. C.01 software [88]. Full geometry optimization of the Ru(II) complexes under study was performed in their S_0_ ground, and first triplet excited T_1_ states without symmetry constraints, employing the PBE0 functional of Perdew, Burke, and Ernzerhof [89,90,91,92,93,94]. The effective core potential, LanL2DZ basis set [95,96,97,98], was used for the Ru metal centre, while for all non-metal atoms, we employed the 6-31G(d,p) basis set as implemented in Gaussian16W, Rev. C.01 software. Accordingly, the computational protocol used for our calculation is denoted as PBE0/LanL2DZ(Ru)U6-31G(d,p)(E). Water solvent effects were taken into account employing the Polarizable Continuum Model (PCM) using the integral equation formalism variant (IEF-PCM), as implemented in the Gaussian16W, Rev. C.01 software, being the default method (self-consistent reaction field (SCRF)) [99]. The Natural Bond Orbital (NBO) method was performed according to the Weinhold et al. methodology [73]. Time-dependent density functional theory (TD-DFT) calculations [100,101,102,103] were performed, taking into account the 30 lowest excited states. Natural Transition Orbitals (NTOs) were employed [85] to account for a simpler description of the electronic transitions appearing in the simulated UV-Vis spectra. Parts of the results obtained from our quantum chemical calculations were also analyzed with the aid of the Multiwfn 3.8 [104] and GaussSum 2.2.6.1 [105] wave function analyzers.

## 4. Conclusions

In the present study, the effect of the axial ligand X of the [Ru(salen)(X)(CO)]^0/−1^ on the Ru-CO photodissociation was studied by means of DFT/TDFT calculations. The effects of the axial ligands X, trans to the CO ligand, on the Ru-CO bond photo-dissociation could be well described employing the, ^13^C NMR based, *trans*-philicity parameter. This is substantiated by the fact that the *trans*-philicity parameter, Δ*σ*^13^C correlates very well with other parameters proposed as descriptors of the X ligand effect, i.e., the STE/*trans*-influence quantified by the Ru-CO bond length, the MLEP parameter quantified by the *v*_s_(Ru-CO) stretching frequency and the *IBDE* parameter. It should be stressed here that Δ*σ*^13^C has the potential to be used as an NMR probe for the Ru-CO bond dissociation. All the above parameters converge to the conclusion that ligands like CNH^−^, NHC, and P(OH)_3_ exert the strongest effects on the Ru-CO bond in the Ru(II) systems under study. In contrast, ligands like Cl^−^, NO_3_^−,^ and DMSO exert the weakest effects on the Ru-CO bond. In order to delineate the nature of the Ru-CO bond, we employed a multitude of theoretical methods, such as NBO, NEDA, and NOCV. These methods revealed that the Ru-CO bond exhibits a composite nature, as expected for a metal–ligand interaction. Thus, the covalent nature of the Ru-CO bond arises from *σ* bonding, *σ*(Ru-CO) orbitals and is reflected in the respective WBI values equivalent to a single covalent bond. The opposite natural charges found for the Ru and C atoms signify the electrostatic nature of the Ru-CO bond, which is further corroborated by the high values of the electric term *EL* obtained from NEDA. The latter also gave very high *CT* values, meaning that charge transfer should also be important in the Ru-CO metal–ligand interaction. The charge transfer between the Ru metal centre and the CO ligand is clearly illustrated by the NOCVs method, which reveals that CO to Ru donation and back–donation contribute equally to the stability of the complexes.

Next, based on triplet PES scans, we unfolded the mechanism of the Ru-CO photodissociation of the Ru(II) complexes under study. Thus, CO photo-release by the latter proceeds upon the population of a dissociative ^3^MC state, which exhibits a conical intersection with the ^3^MLCT state initially obtained upon excitation from the S_0_ ground state. The ^3^MLCT to ^3^MC should be facile since the magnitude of their conical intersection is marginal.

Finally, we have calculated the photophysical properties of the Ru(II) salen system under study. Accordingly, these complexes exhibit absorptions spanning the UV-Vis region. Those appearing in the visible region are the most interesting since UV irradiation is considered to be not very suitable for tissue penetration. On the other hand, these Ru(II) salen complexes could exert a dual action, i.e., as photoCORMs and theranostics concurrently, since they are predicted to be luminescent red emitters. Overall, the present theoretical study has demonstrated that with a proper selection of the axial ligands in Ru(II) octahedral complexes, we could synthesize novel photoCORMs, which could also act as theranostics as well.

## Figures and Tables

**Table 1:** Δ*σ*^13^C, MLEP, and iBDE parameters of the [Ru(salen)(CO)X]^0/−1^ complexes in their ground S_0_ state, at the PBE0/LanL2DZ(Ru)U6-31G(d,p)(E) level of theory in water solvent.

Complex	Ligand X	Δ*σ*^13^C (ppm)	MLEP (cm^−1^)	iBDE (kcal/mol)
**1**	Cl^−^	18.1	547	58.7
**2**	F^−^	21.5	538	57.8
**3**	SCN^−^	20.3	535	55.3
**4**	DMSO	15.4	548	60.2
**5**	Phosphabenzene	26.5	471	44.5
**6**	Phosphole	25.6	501	46.4
**7**	TPH	32.4	458	40.9
**8**	CN^−^	33.0	466	40.7
**9**	N_3_^−^	21.1	533	55.2
**10**	NO_3_^−^	13.2	548	59.7
**11**	CNH^−^	36.3	455	36.0
**12**	NHC	32.5	455	39.6
**13**	P(OH)_3_	32.8	465	38.2
**14**	PF_3_	32.1	425	38.0
**15**	PH_3_	27.5	494	45.1

**Table 2:** Selected natural charges, *Q*, Wiberg Bond Indices, *WBI*, and natural electron configuration, *nec* of the [Ru(salen)(CO)X]^0/−1^ complexes in S_0_ state, at the PBE0/LanL2DZ(Ru)U6-31G(d,p)(E) level of theory in water solvent.

Complex	*Q*		*WBI*		*nec*	
	Ru	C	Ru-C	C-O	C	O
**1**	−0.131	0.788	1.361	2.031	2s^1.05^2p^2.11^	2s^1.70^2p^4.78^
**2**	−0.013	0.731	1.347	1.999	2s^1.10^2p^2.12^	2s^1.70^2p^4.81^
**3**	−0.189	0.793	1.323	2.048	2s^1.06^2p^2.09^	2s^1.70^2p^4.77^
**4**	−0.029	0.780	1.356	2.034	2s^1.06^2p^2.11^	2s^1.70^2p^4.78^
**5**	−0.368	0.811	1.204	2.104	2s^1.09^2p^2.05^	2s^1.70^2p^4.75^
**6**	−0.325	0.779	1.258	2.056	2s^1.10^2p^2.07^	2s^1.70^2p^4.78^
**7**	−0.370	0.790	1.171	2.101	2s^1.12^2p^2.04^	2s^1.70^2p^4.75^
**8**	−0.332	0.772	1.171	2.084	2s^1.14^2p^2.04^	2s^1.70^2p^4.77^
**9**	−0.109	0.775	1.320	2.032	2s^1.08^2p^2.10^	2s^1.70^2p^4.79^
**10**	−0.026	0.787	1.363	2.035	2s^1.05^2p^2.11^	2s^1.70^2p^4.78^
**11**	−0.326	0.805	1.095	2.144	2s^1.13^2p^2.01^	2s^1.70^2p^4.73^
**12**	−0.251	0.761	1.146	2.090	2s^1.15^2p^2.03^	2s^1.70^2p^4.77^
**13**	−0.501	0.799	1.127	2.131	2s^1.12^2p^2.02^	2s^1.70^2p^4.74^
**14**	−0.548	0.830	1.110	2.160	2s^1.10^2p^2.02^	2s^1.70^2p^4.72^
**15**	−0.343	0.805	1.217	2.098	2s^1.09^2p^2.05^	2s^1.70^2p^4.75^

**Table 3:** Total interaction energy, Δ*Ε*, electrical term, EL, charge transfer term, CT, and CORE term for the Ru-CO bond of [Ru(salen)(CO)X]^0/−1^ complexes in their ground S_0_ state, obtained from NEDA, at the PBE0/LanL2DZ(Ru)U6-31G(d,p)(E) level of theory in water solvent.

Complex	Δ*E*	*EL*	*CT*	*CORE*
**1**	−56.6	−151.8	−326.0	421.2
**2**	−55.7	−144.2	−313.0	401.5
**3**	−53.2	−151.0	−318.9	416.6
**4**	−60.5	−151.1	−320.8	411.5
**5**	−44.7	−144.6	−285.6	385.4
**6**	−43.8	−149.0	−300.3	405.5
**7**	−40.3	−142.6	−269.2	371.5
**8**	−39.6	−140.7	−266.6	367.7
**9**	−53.7	−151.9	−322.4	420.6
**10**	−59.6	−151.5	−325.4	417.3
**11**	−36.6	−138.1	−252.6	354.2
**12**	−38.5	−139.5	−258.1	359.0
**13**	−37.8	−135.9	−257.1	355.1
**14**	−39.0	−137.3	−261.9	360.1
**15**	−45.7	−141.1	−287.1	382.6

**Table 4:** Selected principal singlet–singlet optical transitions and corresponding wavelengths, *λ*, oscillator strengths, *f* transition dipole moments, *TDM* and % metal charge transfer, %*CT* in the absorption spectrum of **1** at the TD-DFT-PBEO/Def2-TZVP level in water solvent.

Excitation (%Composition)	*λ* (nm)	*f*	*TDM*	%*CT*
H → L (94%)	404	0.056	0.745	10
H → L+1 (91%)	395	0.066	0.858	10
H-1 → L (89%)	331	0.036	0.393	3
H-1 → L+1 (85%)	322	0.077	0.813	3
H-3 → L (66%)	295	0.175	1.697	11
H-3 → L (28%), H-3 → L+1 (24%), H-3 → L+2 (21%)	293	0.149	1.434	10
H-5 → L (13%), H-3 → L+3 (18%), H-1 → L+3 (35%)	249	0.087	0.711	−13
H-6 → L+1 (17%), H-5 → L+1 (24%), H → L+6 (14%)	243	0.206	1.647	1

**Table 5:** Vertical emission maxima, *λ*_max_ and 0–0 transition, *E*_0–0_, vibrational reorganization energy, *λ*_vib_, and T_1_–S_0_ energy gap, Δ*E*_T1–S0_ calculated for [Ru(salen)(CO)X]^0/−1^ complexes, at the PBE0/LanL2DZ(Ru)U6-31G(d,p)(E) level of theory in water solvent. ^[a]^.

Complex	*λ*_max_	*E*_0–0_	λ_vib_	Δ*E*_T1–S0_
**1**	843	47.5	15.6	49.5
**2**	667	51.9	9.8	52.7
**3**	813	47.6	14.6	49.8
**4**	813	47.7	14.5	49.7
**5**	2598	35.4	25.9	37.0
**6**	835	44.6	12.5	46.7
**7**	639	54.0	10.1	54.8
**8**	665	52.4	10.1	53.1
**9**	811	46.1	12.8	48.1
**10**	792	48.0	14.1	50.2
**11**	607	56.0	9.9	57.0
**12**	683	51.5	10.2	52.1
**13**	642	49.9	7.6	52.2
**14**	793	50.1	16.0	52.1
**15**	3030	38.6	31.2	40.7

[a] All values are in kcal/mol except for *λ*_ver_, which are in nm.

## Data Availability

Data are contained within the article and Supplementary Materials.

## Supplementary Materials

The following supporting information can be downloaded at: https://www.mdpi.com/article/10.3390/molecules30051147/s1, Figure S1. Linear correlation of Δ*σ*^13^C versus *v*(Ru-C) (MLEP). Figure S2. (a) Linear correlation of Δ*σ*^13^C versus iBDE. Figure S3. Linear correlation of Δ*σ*^13^C versus WBI. Figure S4. Linear correlations of Δ*σ*^13^C versus NEDA parameters. Figure S5. 3D isosurfaces of NOCV constituting Pairs 1, 2 and 3. Figure S6. PES scan of the manifold of the 10 lowest triplet excited states of 2–5 in S_0_ state, along the Ru-CO bond stretch calculated at the TD-DFT/PBE0/LanL2DZ(Ru)U6-31G(d,p)(E) level of theory, in water solvent. Figure S7. PES scan of the manifold of the 10 lowest triplet excited states of 6–9 in S_0_ state, along the Ru-CO bond stretch calculated at the TD-DFT/PBE0/LanL2DZ(Ru)U6-31G(d,p)(E) level of theory, in water solvent. Figure S8. PES scan of the manifold of the 10 lowest triplet excited states of 10–13 in S_0_ state, along the Ru-CO bond stretch calculated at the TD-DFT/PBE0/LanL2DZ(Ru)U6-31G(d,p)(E) level of theory, in water solvent. Figure S9. PES scan of the manifold of the 10 lowest triplet excited states of 14 and 15 in S_0_ state, along the Ru-CO bond stretch calculated at the TD-DFT/PBE0/LanL2DZ(Ru)U6-31G(d,p)(E) level of theory, in water solvent. Figure S10. Linear correlation between the conical intersection energy gap, Δ*E* and the transphilicity parameter, Δ*σ*^13^C. Figure S11. Simulated UV-Vis absorption spectrum of 2–5 (FWHM = 0.1) calculated at the TDDFT/PBE0/LanL2DZ(Ru)U6-31G(d,p)(E) level of theory, in water solvent. Figure S12. Simulated UV-Vis absorption spectrum of 6–9 (FWHM = 0.1) calculated at the TDDFT/PBE0/LanL2DZ(Ru)U6-31G(d,p)(E) level of theory, in water solvent. Figure S13. Simulated UV-Vis absorption spectrum of 10–15 (FWHM = 0.1) calculated at the TDDFT/PBE0/LanL2DZ(Ru)U6-31G(d,p)(E) level of theory, in water solvent. Figure S14. 3D isosurfaces of the MOs relevant to the electronic transitions. Table S1. Selected equatorial structural parameters (bond lengths in Å, bond angles in º) of the [Ru(salen)(CO)X]^0/−1^ complexes in their ground S_0_ state, calculated at the PBE0/Lanl2dz(Ru)U6-31G(d,p)(E) level of theory, in water solvent. Table S2. Selected structural parameters (bond lengths in Å, bond angles in º) of the [Ru(salen)X]^0/−1^ complexes in their ground S_0_ state, at the PBE0/LanL2DZ(Ru)U6-31G(d,p)(E) level of theory in water solvent. Table S3 Selected structural parameters (bond lengths in Å, bond angles in deg) of the [Ru(salen)(H2O)X]^0/−1^ complexes in their ground S_0_ state, at the PBE0/Lanl2dz(Ru)U6-31G(d,p)(E) level of theory, in water solvent. Table S4. *σ*^13^C chemical shielding tensors and *δ*^13^C NMR of the CO ligand of complexes 1–15 versus the TMS reference at the PBE0/LanL2DZ(Ru)U6-31G(d,p)(E) level of theory in water solvent. Table S5. Bonding *σ*(Ru-CO) and antibonding *σ**(Ru-CO) ΝΒOs of the [Ru(salen)(CO)X]^0/−1^ complexes in their ground S_0_ state, calculated at the PBE0/LanL2DZ(Ru)U6-31G(d,p)(E) level of theory in water solvent. Table S6. Energies, eigenvalues, linear combinations and % compositions of the dominant NOCV pairs of complexes 1–15. Table S7. Cartesian coordinates and energetic data.
