# Supplementary material for: Axial Ligand Effects on the Mechanism of Ru-CO Bond Photodissociation and Photophysical Properties of Ru(II)-Salen PhotoCORMs/Theranostics: A Density Functional Theory Study
Source: Molecules. 2025 Mar 3;30(5):1147. doi: 10.3390/molecules30051147 (PMC11901629; doi:10.3390/molecules30051147)
Supplement: Supplementary file 1 [file molecules-30-01147-s001.zip › molecules-3499928-supplementary.pdf]

Supplementary Material

Axial Ligand Effects on the Mechanism of Ru-CO bond Photodissociation and Photophysical Properties of Ru(II)-Salen PhotoCORMs/Theranostics: A DFT study

Niq Catevas and Athanassios Tsipis*

Axial Ligand Effects on the Mechanism of Ru-CO bond Photodissociation and Photophysical Properties of Ru(II)-Salen PhotoCORMs/Theranostics: A DFT study

Niq Catevas[a] and Athanassios Tsipis*[a]

[a] Prof. Dr. A. Tsipis, PhD student N. Catevas
Department of Chemistry
University of Ioannina
T.Th.1186, Campus Ioannina University, Ioannina, Greece
E-mail: attsipis@uoi.gr

Supplementary Information

Contents

Figure S1. Linear correlation of $\Delta\sigma^{13}$C versus v(Ru-C) (MLEP).
Figure S2. (a) Linear correlation of $\Delta\sigma^{13}$C versus iBDE.
Figure S3. Linear correlation of $\Delta\sigma^{13}$C versus WBI.
Figure S4. Linear correlations of $\Delta\sigma^{13}$C versus NEDA parameters.
Figure S5. 3D isosurfaces of NOCV constituting Pairs 1, 2 and 3.
Figure S6. PES scan of the manifold of the 10 lowest triplet excited states of **2** - **5** in S_0 state, along the Ru-CO bond stretch calculated at the TD-DFT/ PBE0/LanL2DZ(Ru)U6-31G(d,p)(E) level of theory, in water solvent.
Figure S7. PES scan of the manifold of the 10 lowest triplet excited states of **6** - **9** in S_0 state, along the Ru-CO bond stretch calculated at the TD-DFT/ PBE0/LanL2DZ(Ru)U6-31G(d,p)(E) level of theory, in water solvent.
Figure S8. PES scan of the manifold of the 10 lowest triplet excited states of **10** - **13** in S_0 state, along the Ru-CO bond stretch calculated at the TD-DFT/ PBE0/LanL2DZ(Ru)U6-31G(d,p)(E) level of theory, in water solvent.
Figure S9. PES scan of the manifold of the 10 lowest triplet excited states of **14** and **15** in S_0 state, along the Ru-CO bond stretch calculated at the TD-DFT/ PBE0/LanL2DZ(Ru)U6-31G(d,p)(E) level of theory, in water solvent.
Figure S10. Linear correlation between the conical intersection energy gap, ΔE and the transphilicity parameter, $\Delta\sigma^{13}$C.
Figure S11. Simulated UV-Vis absorption spectrum of **2** – **5** (FWHM = 0.1) calculated at the TDDFT/PBE0/LanL2DZ(Ru)U6-31G(d,p)(E) level of theory, in water solvent.
Figure S12. Simulated UV-Vis absorption spectrum of **6** – **9** (FWHM = 0.1) calculated at the TDDFT/PBE0/LanL2DZ(Ru)U6-31G(d,p)(E) level of theory, in water solvent.
Figure S13. Simulated UV-Vis absorption spectrum of **10** – **15** (FWHM = 0.1) calculated at the TDDFT/PBE0/LanL2DZ(Ru)U6-31G(d,p)(E) level of theory, in water solvent.
Figure S14. 3D isosurfaces of the MOs relevant to the electronic transitions.
Table S1. Selected equatorial structural parameters (bond lengths in Å, bond angles in °) of the $[Ru(salen)(CO)X]^{0/-1}$ complexes in their ground S_0 state, calculated at the PBE0/Lanl2dz(Ru)U6-31G(d,p)(E) level of theory, in water solvent.
Table S2. Selected structural parameters (bond lengths in Å, bond angles in °) of the $[Ru(salen)X]^{0/-1}$ complexes in their ground S_0 state, at the PBE0/LanL2DZ(Ru)U6-31G(d,p)(E) level of theory in water solvent.
Table S3 Selected structural parameters (bond lengths in Å, bond angles in °) of the $[Ru(salen)(H_2O)X]^{0/-1}$ complexes in their ground S_0 state, at the PBE0/Lanl2dz(Ru)U6-31G(d,p)(E) level of theory, in water solvent.
Table S4. σ^{13}C chemical shielding tensors and δ^{13}C NMR of the CO ligand of complexes 1 - 15 versus the TMS reference at the PBE0/LanL2DZ(Ru)U6-31G(d,p)(E) level of theory in water solvent.
Table S5. Bonding σ(Ru-CO) and antibonding σ^*(Ru-CO) NBOs of the $[Ru(salen)(CO)X]^{0/-1}$ complexes in their ground S_0 state, calculated at the PBE0/LanL2DZ(Ru)U6-31G(d,p)(E) level of theory in water solvent.
Table S6. Energies, eigenvalues, linear combinations and % compositions of the dominant NOCV pairs of complexes **1** – **15**.
Table S7. Cartesian coordinates and energetic data.

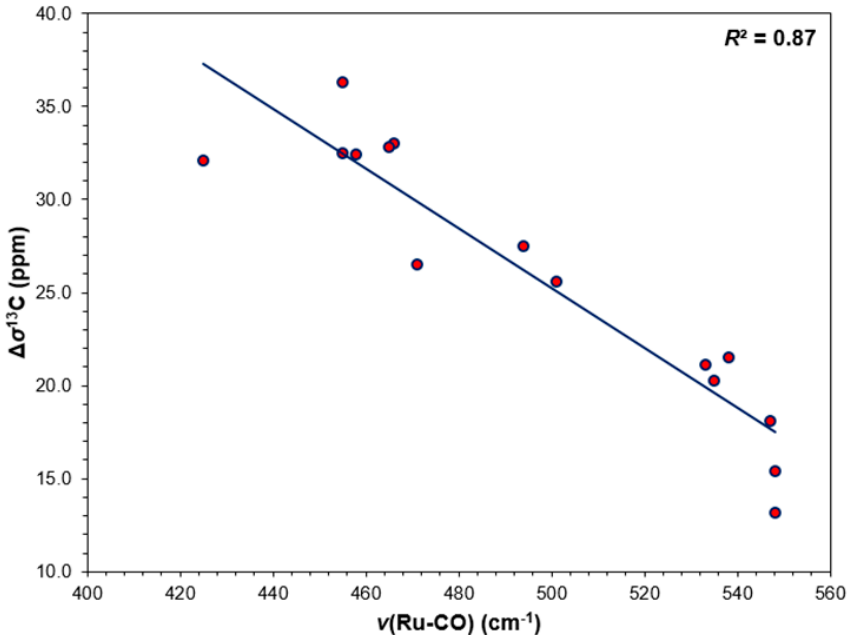

Figure S1. Linear correlation of $\Delta\sigma^{13}C$ versus v(Ru-C) (MLEP).

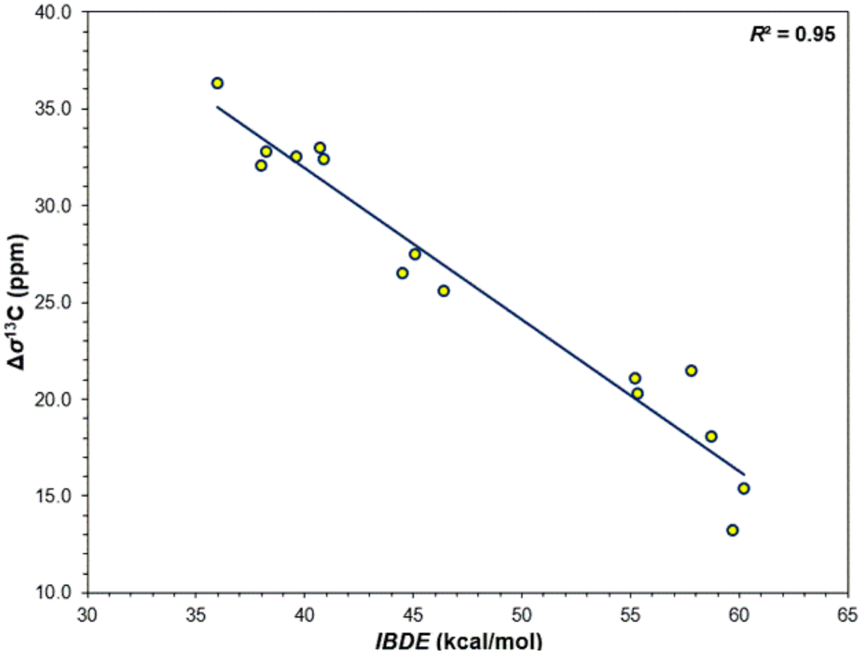

Figure S2. Linear correlation of $\Delta\sigma^{13}C$ versus iBDE.

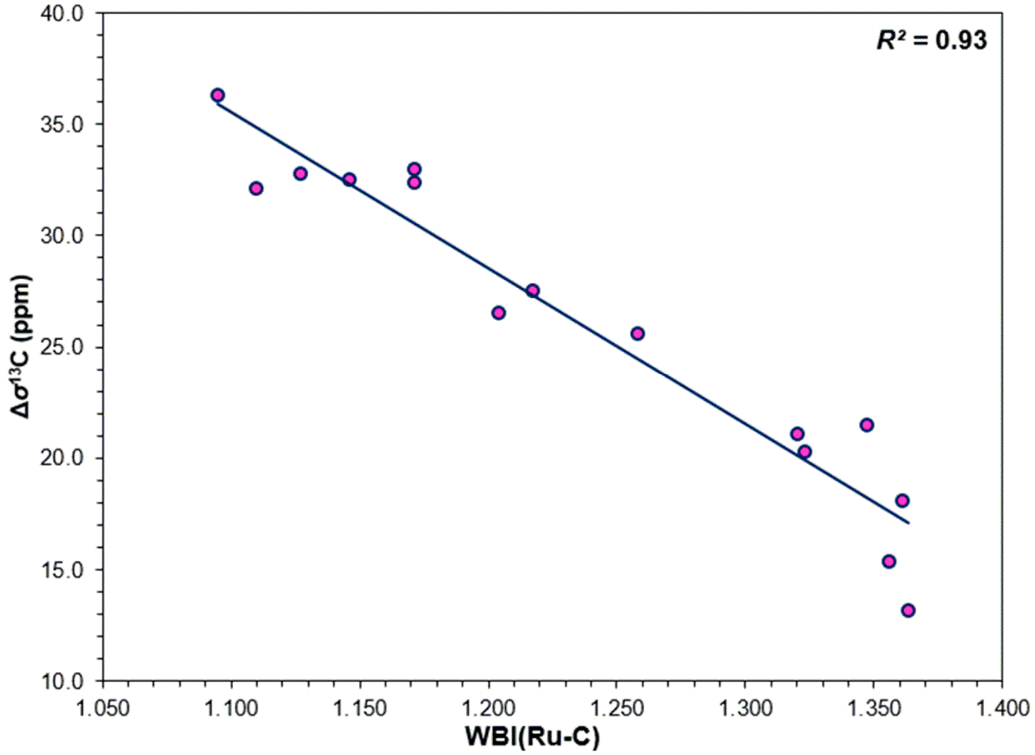

Figure S3. Linear correlation of $\Delta\sigma^{13}C$ versus WBI.

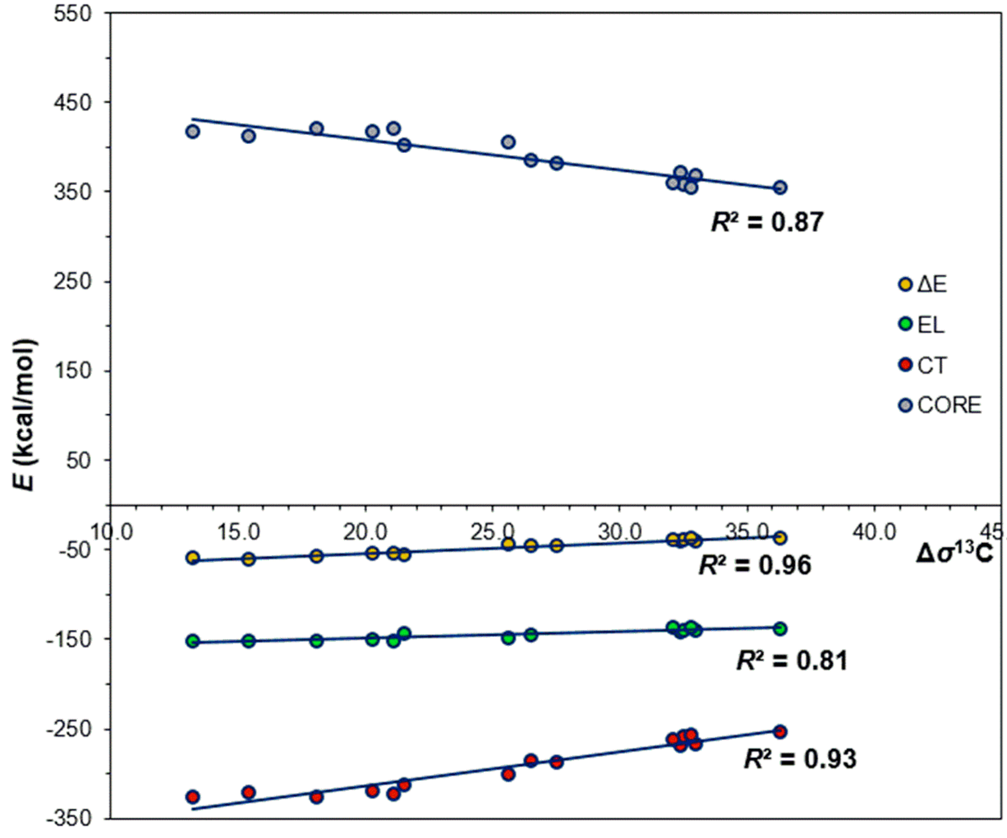

Figure S4. Linear correlations of $\Delta\sigma^{13}C$ versus NEDA parameters.

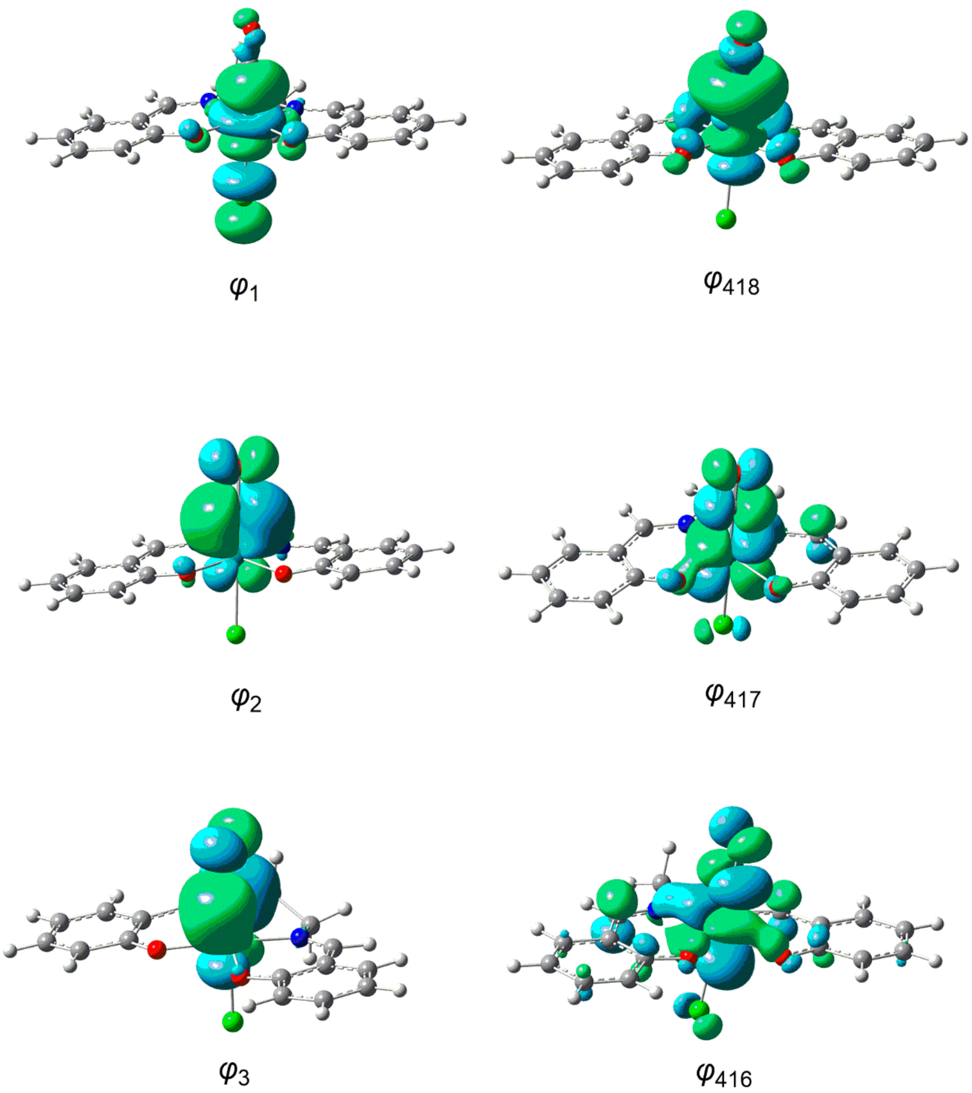

Figure S5. 3D isosurfaces of NOCV constituting Pairs 1, 2 and 3.

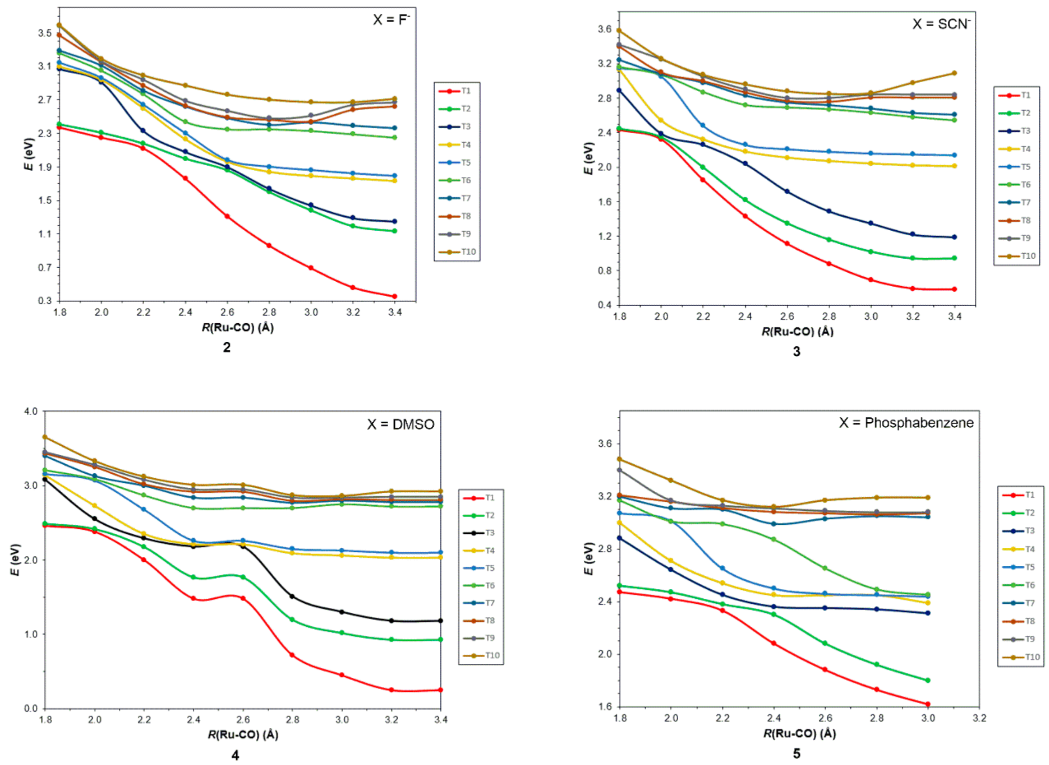

Figure S6. PES scan of the manifold of the 10 lowest triplet excited states of **2** - **5** in S_0 state, along the Ru-CO bond stretch calculated at the TD-DFT/ PBE0/LanL2DZ(Ru)U6-31G(d,p)(E) level of theory, in water solvent.

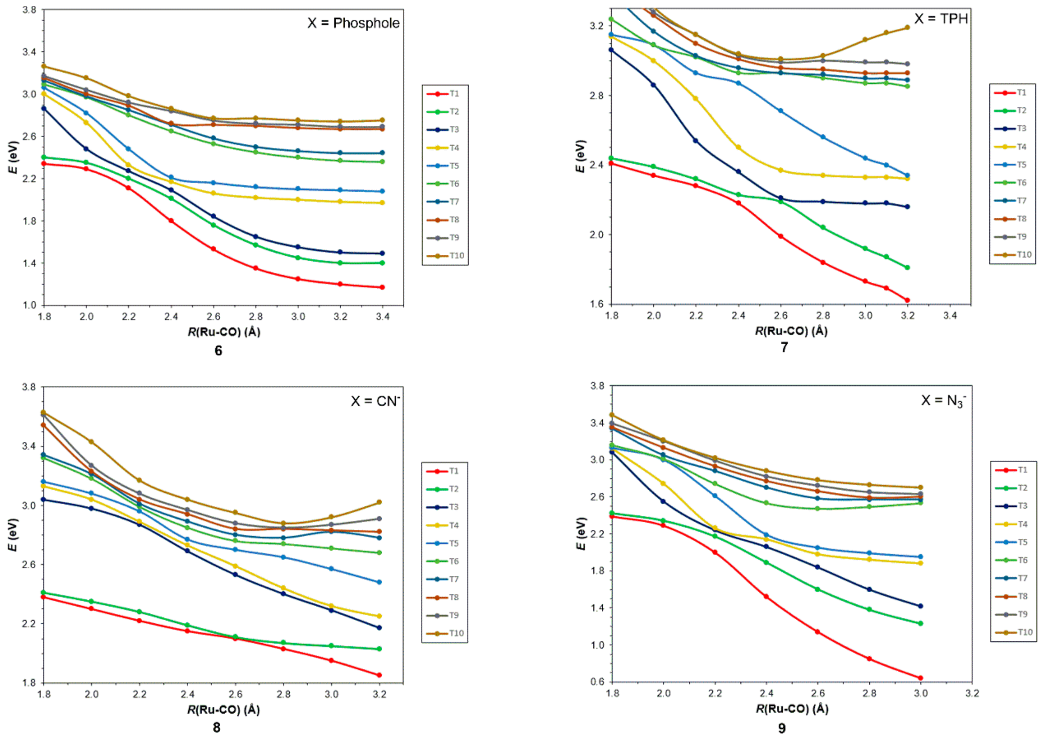

Figure S7. PES scan of the manifold of the 10 lowest triplet excited states of **6** - **9** in S_0 state, along the Ru-CO bond stretch calculated at the TD-DFT/ PBE0/LanL2DZ(Ru)U6-31G(d,p)(E) level of theory, in water solvent.

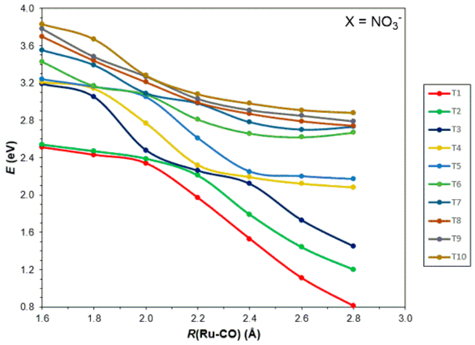

10

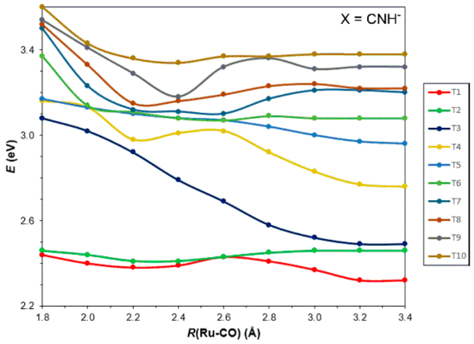

11

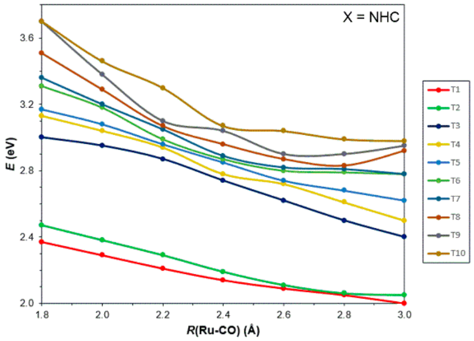

12

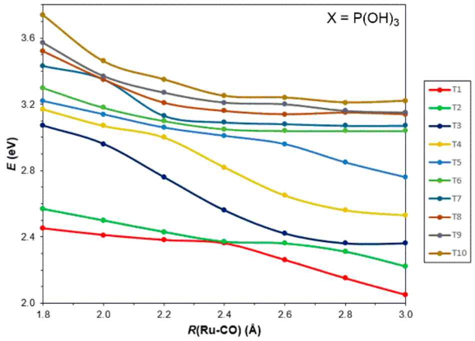

13

Figure S8. PES scan of the manifold of the 10 lowest triplet excited states of **10** - **13** in S_0 state, along the Ru-CO bond stretch calculated at the TD-DFT/ PBE0/LanL2DZ(Ru)U6-31G(d,p)(E) level of theory, in water solvent.

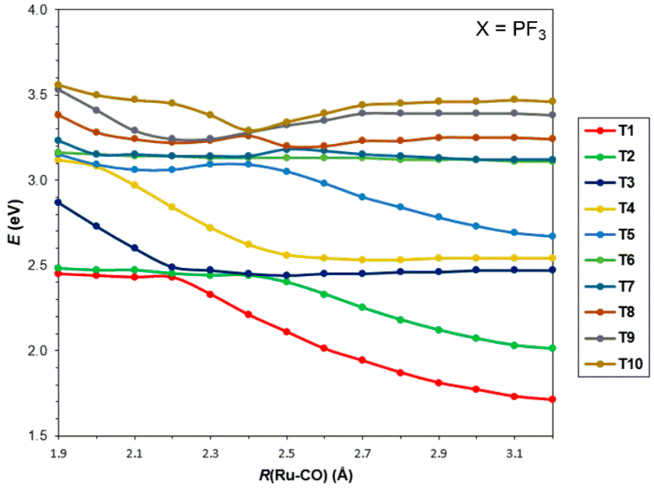

14

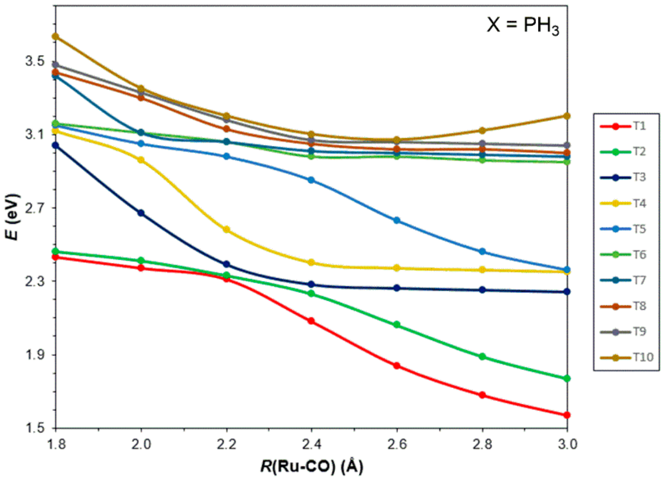

15

Figure S9. PES scan of the manifold of the 10 lowest triplet excited states of **14** and **15** in S_0 state, along the Ru-CO bond stretch calculated at the TD-DFT/ PBE0/LanL2DZ(Ru)U6-31G(d,p)(E) level of theory, in water solvent.

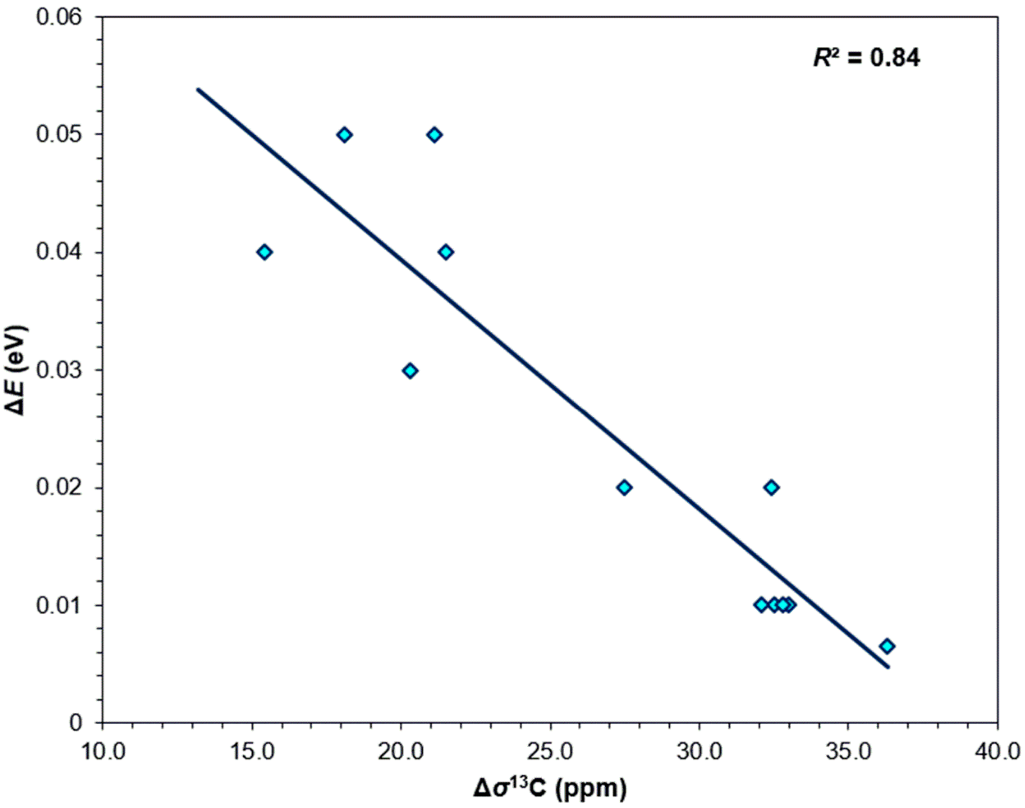

Figure S10. Linear correlation between the conical intersection energy gap, Δ*E* and the transphilicity parameter, $\Delta\sigma^{13}$C.

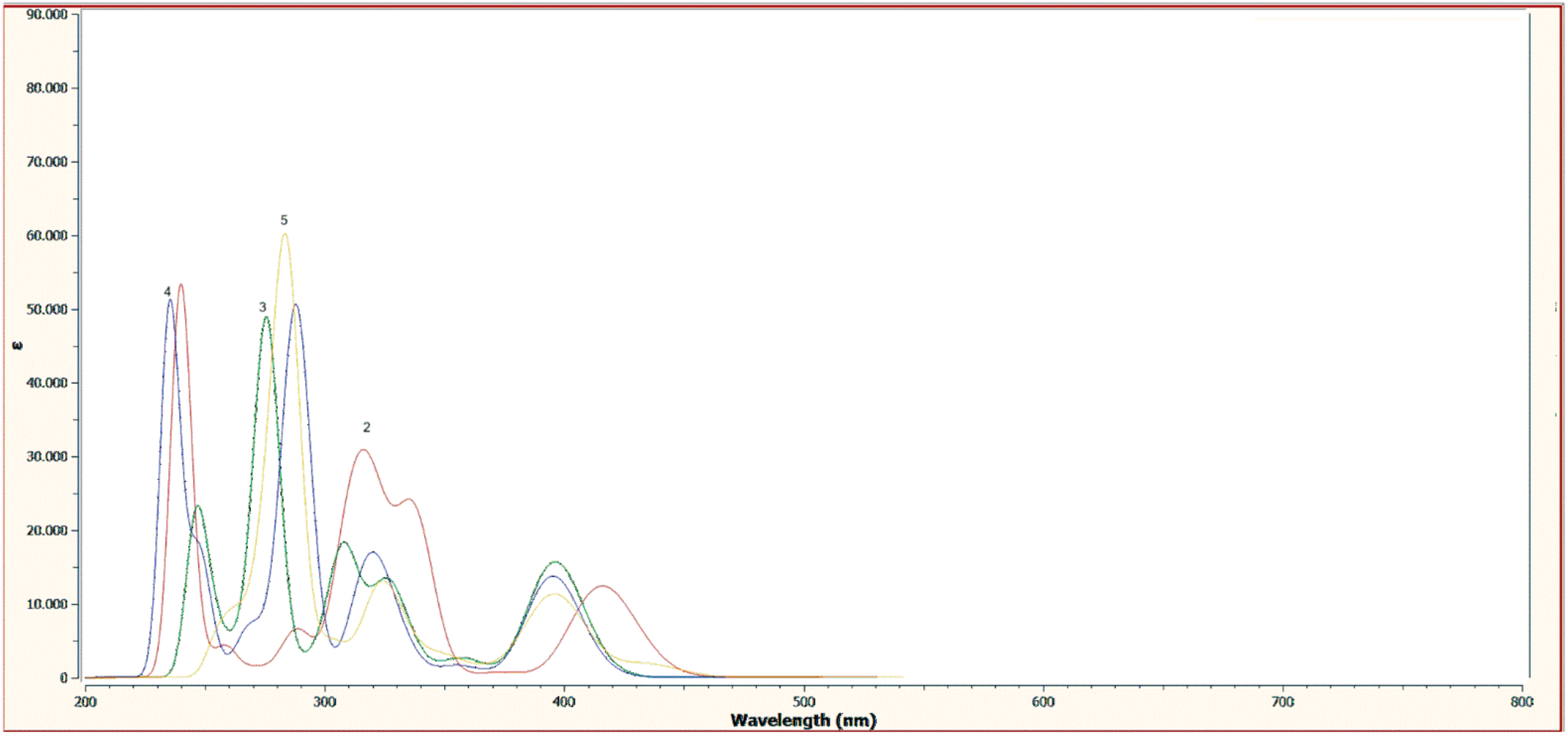

Figure S11. Simulated UV-Vis absorption spectrum of **2** – **5** (FWHM = 0.1) calculated at the TDDFT/PBE0/LanL2DZ(Ru)U6-31G(d,p)(E) level of theory, in water solvent.

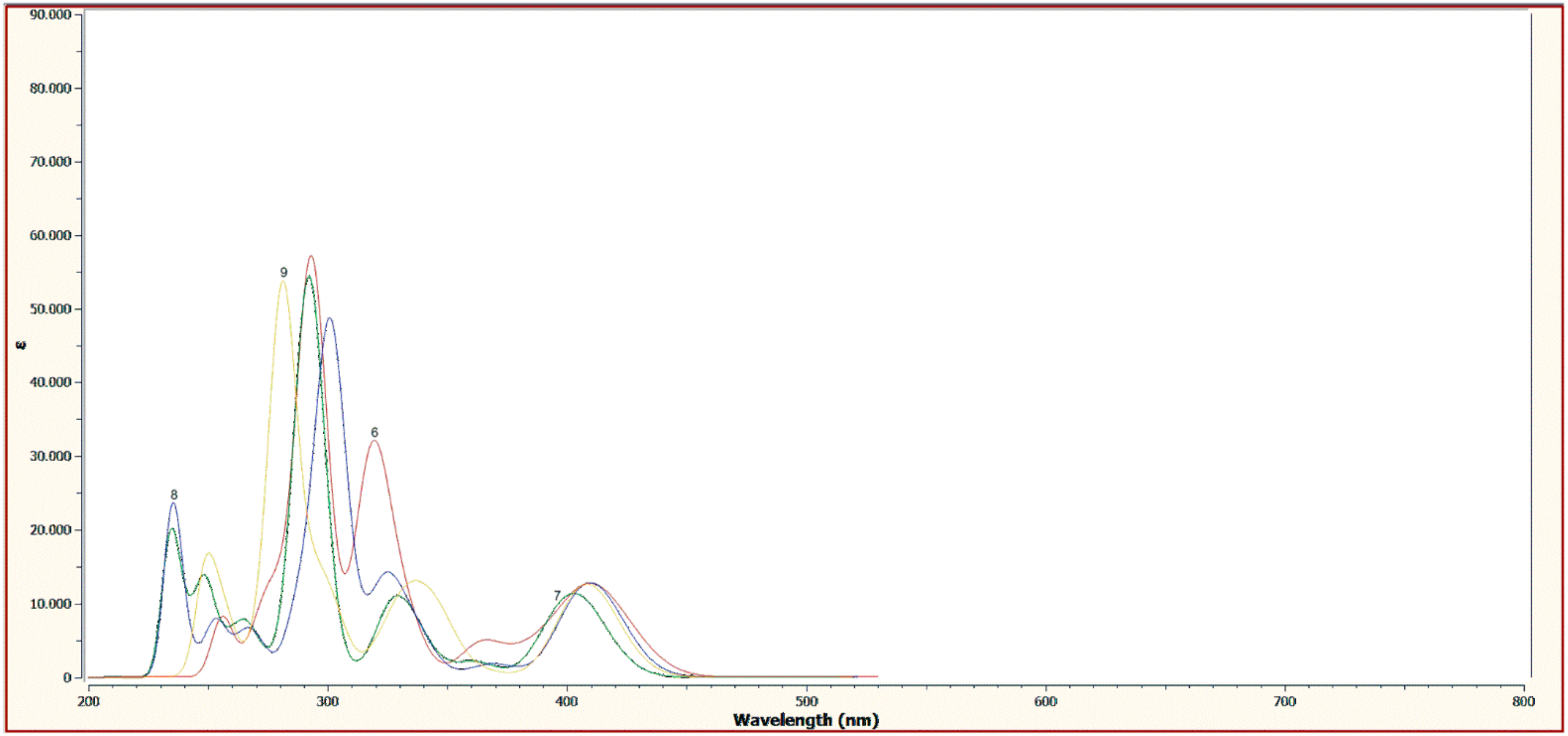

Figure S12. Simulated UV-Vis absorption spectrum of **6** – **9** (FWHM = 0.1) calculated at the TDDFT/PBE0/LanL2DZ(Ru)U6-31G(d,p)(E) level of theory, in water solvent.

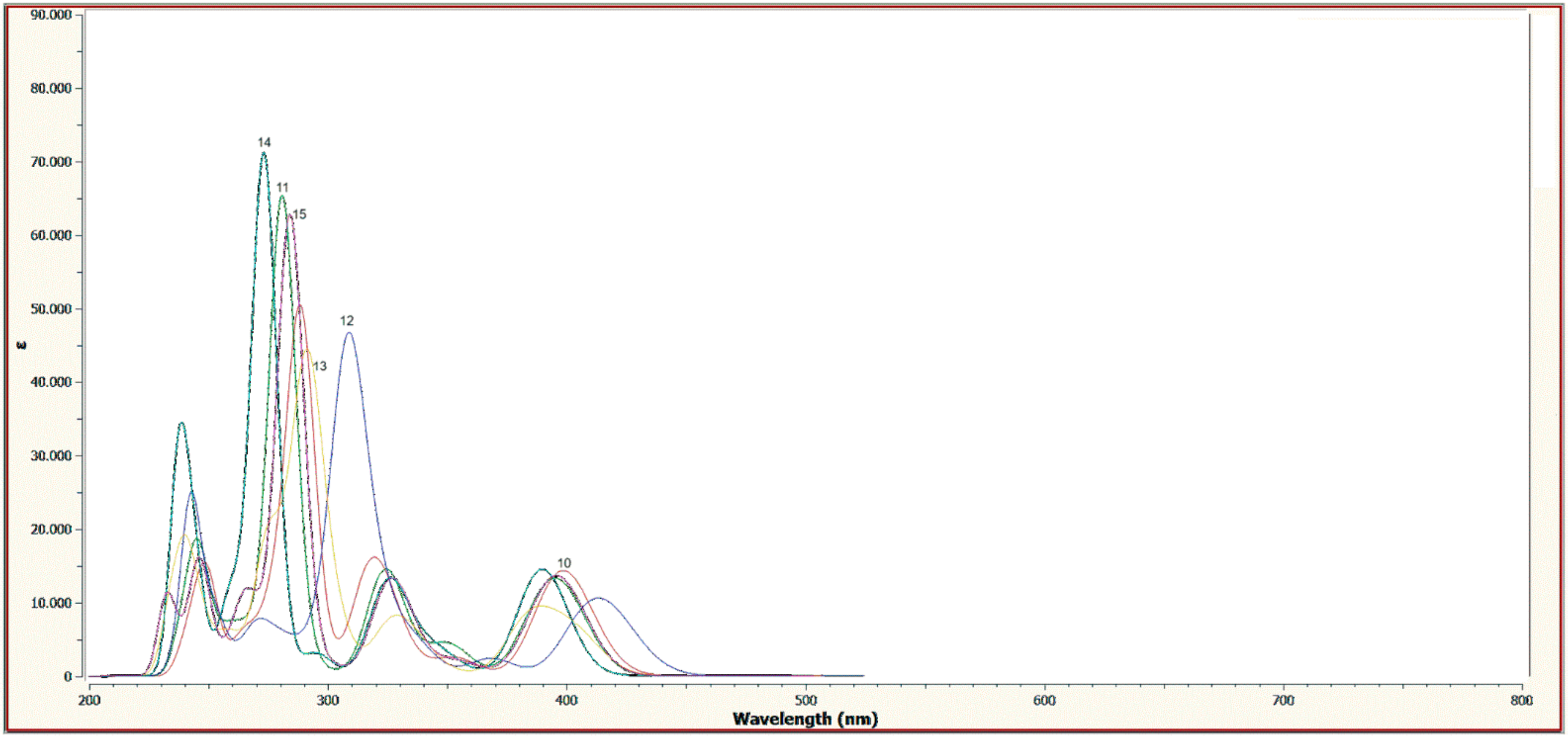

Figure S13. Simulated UV-Vis absorption spectrum of **10** – **15** (FWHM = 0.1) calculated at the TDDFT/PBE0/LanL2DZ(Ru)U6-31G(d,p)(E) level of theory, in water solvent.

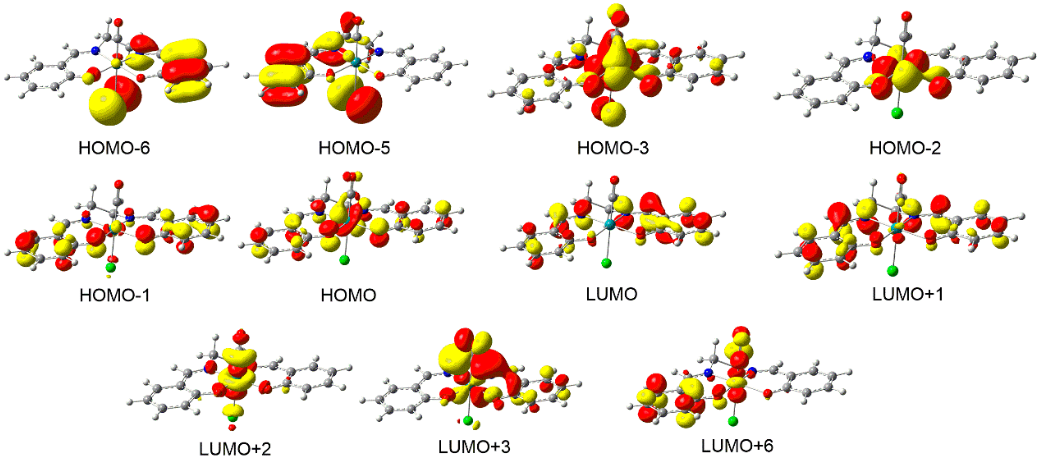

Figure S14. 3D isosurfaces of the MOs relevant to the electronic transitions appearing in the simulated absorption spectrum of **1**.

Table S1. Selected structural parameters (bond lengths in Å, bond angles in º) of the $[Ru(salen)(CO)X]^{0/-1}$ complexes in their ground S_0 state, at the PBE0/LanL2DZ(Ru)U6-31G(d,p)(E) level of theory, in water solvent.

Complex	Ligand X	R_e(Ru-X)	R_e(Ru-CO)	R_e(C-O)	R_e(Ru-O)[a]	R_e(Ru-N)[a]	$\widehat{Ru\text{-}C\text{-}O}$[a]	$\widehat{O\text{-}Ru\text{-}N}$[a]
**1**	Cl^-	2.577	1.820	1.163	2.099	2.020	179.4	92.1
**2**	F^-	2.015	1.843	1.167	2.103	2.011	179.5	92.0
**3**	SCN^-	2.600	1.829	1.161	2.101	2.021	179.1	92.0
**4**	DMSO	2.189	1.824	1.163	2.091	2.018	178.7	92.2
**5**	Phosphabenzene	2.462	1.864	1.155	2.105	2.027	178.5	92.1
**6**	Phosphole	2.558	1.857	1.161	2.112	2.022	179.2	91.9
**7**	TPH	2.447	1.882	1.155	2.113	2.025	177.9	91.7
**8**	CN^-	2.090	1.897	1.158	2.120	2.022	178.7	91.7
**9**	N_3^-	2.195	1.837	1.163	2.104	2.020	179.4	92.0
**10**	NO_3^-	2.208	1.820	1.162	2.094	2.019	179.2	92.2
**11**	CNH^-	2.043	1.908	1.150	2.111	2.027	177.8	92.0
**12**	NHC	2.099	1.906	1.157	2.123	2.020	178.0	91.5
**13**	$P(OH)_3$	2.400	1.897	1.152	2.117	2.022	177.7	91.8
**14**	PF_3	2.369	1.890	1.148	2.096	2.029	177.9	92.4
**15**	PH_3	2.476	1.863	1.156	2.104	2.026	178.4	92.0

[a] Number refer to average values.

Table S2. Selected structural parameters (bond lengths in Å, bond angles in º) of the [Ru(salen)X]$^{0/-1}$ complexes in their ground S_0 state, at the PBE0/LanL2DZ(Ru)U6-31G(d,p)(E) level of theory in water solvent.

Complex	R_e(Ru-X)	R_e(Ru-N)	R_e(Ru-O)	$\widehat{X\text{-}Ru\text{-}O}$	$\widehat{N\text{-}Ru\text{-}N}$	$\widehat{O\text{-}Ru\text{-}O}$	$\widehat{O\text{-}Ru\text{-}N}$
**1**	2.392	1.994	2.096	96.3	83.9	92.3	91.6
**2**	1.965	1.984	2.100	90.6	84.0	92.2	91.8
**3**	2.329	1.998	2.094	96.2	83.6	92.4	91.7
**4**	2.059	1.997	2.094	87.0	83.8	92.7	91.8
**5**	2.160	2.011	2.079	95.7	83.1	91.1	92.2
**6**	2.271	1.992	2.101	94.6	83.7	92.0	91.5
**7**	2.199	2.007	2.089	93.2	83.2	91.8	91.9
**8**	1.906	2.000	2.096	97.4	83.5	92.1	91.7
**9**	2.044	1.994	2.099	93.9	83.7	92.4	91.7
**10**	2.035	1.997	2.097	99.1	83.5	92.2	91.8
**11**	1.797	2.017	2.069	99.2	82.8	90.8	92.2
**12**	1.926	2.002	2.096	93.1	83.4	92.3	91.9
**13**	2.138	2.010	2.092	93.9	83.2	91.7	92.1
**14**	2.080	2.021	2.066	96.7	82.8	90.8	92.1
**15**	2.184	2.011	2.082	92.0	83.2	92.0	91.9

Table S3 Selected structural parameters (bond lengths in Å, bond angles in º) of the $[Ru(salen)(H_2O)X]^{0/-1}$ complexes in their ground S_0 state, at the PBE0/Lanl2dz(Ru)U6-31G(d,p)(E) level of theory, in water solvent.

Complex	R_e(Ru-OH$_2$)	N-$\widehat{Ru}$-OH$_2$	R_e(Ru-X)	R_e(Ru-O)	R_e(Ru-N)	O-$\widehat{Ru}$-N
**1**	2.190	90.4	2.469	2.111	1.993	91.5
**2**	2.234	89.9	1.995	2.112	1.979	91.7
**3**	2.217	89.7	2.402	2.111	1.997	91.6
**4**	2.185	90.4	2.114	2.103	1.994	91.8
**5**	2.264	92.1	2.203	2.100	2.011	92.0
**6**	2.303	88.8	2.316	2.114	1.997	91.5
**7**	2.291	87.9	2.245	2.102	2.009	91.8
**8**	2.324	88.9	1.939	2.112	1.999	91.6
**9**	2.229	90.3	2.094	2.113	1.993	91.5
**10**	2.185	89.4	2.089	2.109	1.994	91.7
**11**	2.290	91.7	1.830	2.088	2.016	92.2
**12**	2.320	89.4	1.957	2.112	1.999	91.6
**13**	2.293	92.3	2.171	2.099	2.011	91.9
**14**	2.248	90.7	2.118	2.084	2.021	92.3
**15**	2.271	93.3	2.229	2.100	2.010	91.9

Table S4. $\sigma^{13}C$ chemical shielding tensors and $\delta^{13}C$ NMR of the CO ligand of complexes **1** - **15** versus the TMS reference at the PBE0/LanL2DZ(Ru)U6-31G(d,p)(E) level of theory in water solvent.

Complex	$\sigma^{13}C$ (ppm)	$\delta^{13}C$ (ppm)
**1**	-36.38	196.94
**2**	-33.04	196.94
**3**	-34.27	196.94
**4**	-39.14	196.94
**5**	-28.03	196.94
**6**	-28.96	196.94
**7**	-22.16	196.94
**8**	-21.56	196.94
**9**	-33.38	196.94
**10**	-41.33	196.94
**11**	-18.20	196.94
**12**	-22.04	196.94
**13**	-21.72	196.94
**14**	-22.44	196.94
**15**	-26.98	196.94

Table S5. Bonding σ(Ru-CO) and antibonding σ*(Ru-CO) NBOs of the $[Ru(salen)(CO)X]^{0/-1}$ complexes in their ground S_0 state, calculated at the PBE0/LanL2DZ(Ru)U6-31G(d,p)(E) level of theory in water solvent.

	σ(Ru-CO)			
	Linear Comb.[a]	h_{Ru}	h_C	*Occ. Num.*[b]
**1**	$0.565h_{Ru}+0.825h_C$	$sp^{1.44}d^{1.41}$	$sp^{0.56}$	1.837
**2**	$0.578h_{Ru}+0.816h_C$	$sp^{0.62}d^{1.27}$	$sp^{0.53}$	1.931
**3**	$0.558h_{Ru}+0.830h_C$	$sp^{1.36}d^{1.45}$	$sp^{0.56}$	1.828
**4**	$0.591h_{Ru}+0.807h_C$	$sp^{0.56}d^{1.24}$	$sp^{0.56}$	1.918
**5**	$0.570h_{Ru}+0.822h_C$	$sp^{2.21}d^{1.94}$	$sp^{0.54}$	1.904
**6**	$0.562h_{Ru}+0.827h_C$	$sp^{1.70}d^{1.35}$	$sp^{0.53}$	1.897
**7**	$0.556h_{Ru}+0.831h_C$	$sp^{2.30}d^{1.81}$	$sp^{0.53}$	1.908
**8**	$0.540h_{Ru}+0.841h_C$	$sp^{2.08}d^{1.27}$	$sp^{0.50}$	1.906
**9**	$0.587h_{Ru}+0.810h_C$	$sp^{0.55}d^{1.23}$	$sp^{0.54}$	1.922
**10**	$0.593h_{Ru}+0.806h_C$	$sp^{0.54}d^{1.26}$	$sp^{0.56}$	1.916
**11**	$0.546h_{Ru}+0.838h_C$	$sp^{2.71}d^{1.87}$	$sp^{0.51}$	1.911
**12**	$0.533h_{Ru}+0.847h_C$	$sp^{2.29}d^{1.27}$	$sp^{0.50}$	1.903
**13**	$0.513h_{Ru}+0.858h_C$	$sp^{1.02}d^{1.39}$	$sp^{0.52}$	1.798
**14**	$0.559h_{Ru}+0.829h_C$	$sp^{1.73}d^{1.30}$	$sp^{0.53}$	1.897
**15**	$0.570h_{Ru}+0.822h_C$	$sp^{2.19}d^{1.90}$	$sp^{0.54}$	1.905

[a] Linear combination of the NBO [b] Occupation Number

Table S6. Energies, eigenvalues, linear combinations and % compositions of the dominant NOCV pairs of complexes **1 – 15**.

Compound	NOCV Pair No.	Energy	Eigenvalue	Linear Combination	% AO contribution[a]
**1**	1	-80.78	0.70348	$0.703[\varphi_1(r)]^2 - 0.703[\varphi_{418}(r)]^2$	3%s(Ru), 20%d_{z^2}(Ru), -3%s(C), -26%s(C)
	2	-35.04	0.61050	$0.611[\varphi_2(r)]^2 - 0.611[\varphi_{417}(r)]^2$	-21%d_{xz}(Ru), 10%p_x(C)
	3	-33.11	0.58796	$0.588[\varphi_3(r)]^2 - 0.588[\varphi_{416}(r)]^2$	-13%d_{yz}(Ru), 10%p_y(C)
**2**	1	-60.16	0.63390	$0.634[\varphi_1(r)]^2 - 0.634[\varphi_{414}(r)]^2$	8%d_{z^2}(Ru), 3%d_{xz}(Ru), -19%s(C)
	2	-35.92	0.62461	$0.634[\varphi_2(r)]^2 - 0.634[\varphi_{413}(r)]^2$	-23%d_{xz}(Ru), 9%p_x(C)
	3	-40.93	0.59760	$0.598[\varphi_3(r)]^2 - 0.598[\varphi_{412}(r)]^2$	6%py(Ru), 9%d_{z^2}(Ru), -10%s(C)
**3**	1	-77.83	0.69880	$0.699[\varphi_1(r)]^2 - 0.699[\varphi_{446}(r)]^2$	3%s(Ru), 8%d_{z^2}(Ru), 9%d_{yz}(Ru), -3%s(C), -26%s(C)
	2	-33.42	0.59611	$0.596[\varphi_2(r)]^2 - 0.596[\varphi_{445}(r)]^2$	-17%d_{xz}(Ru), -3%d_{xy}(Ru), 11%p_x(C)
	3	-31.45	0.58099	$0.581[\varphi_3(r)]^2 - 0.581[\varphi_{444}(r)]^2$	-3%d_{z^2}(Ru), -6%d_{yz}(Ru), 9%p_y(C)
**4**	1	-77.90	0.67500	$0.675[\varphi_1(r)]^2 - 0.675[\varphi_{490}(r)]^2$	3%s(Ru), 12%d_{yz}(Ru), -28%s(C)
	2	-35.86	0.61302	$0.613[\varphi_2(r)]^2 - 0.613[\varphi_{489}(r)]^2$	-10%d_{xz}(Ru), -14%d_{xy}(Ru), 11%p_x(C)
	3	-31.83	0.57991	$0.580[\varphi_3(r)]^2 - 0.580[\varphi_{488}(r)]^2$	-10%d_{z^2}(Ru), -4%$d_{x^2-y^2}$(Ru), 6%p_y(C), 5%p_z(C)
**5**	1	-67.89	0.65309	$0.653[\varphi_1(r)]^2 - 0.653[\varphi_{513}(r)]^2$	9%$d_{x^2-y^2}$(Ru), -4%s(C), -3%p_y(C), -21%s(C)
	2	-28.12	0.56723	$0.567[\varphi_2(r)]^2 - 0.567[\varphi_{512}(r)]^2$	-12%d_{xy}(Ru), 9%p_x(C)
	3	-26.31	0.53579	$0.536[\varphi_3(r)]^2 - 0.536[\varphi_{511}(r)]^2$	-7%d_{yz}(Ru), 8%p_z(C)
**6**	1	-66.56	0.66085	$0.661[\varphi_1(r)]^2 - 0.661[\varphi_{494}(r)]^2$	12%$d_{x^2-y^2}$(Ru), -4%s(C), -5%p_y(C), -21%s(C)
	2	-30.84	0.59092	$0.591[\varphi_2(r)]^2 - 0.591[\varphi_{493}(r)]^2$	-4%d_{xz}(Ru), -10%d_{xy}(Ru), 8%p_x(C)
	3	-29.42	0.56637	$0.566[\varphi_3(r)]^2 - 0.566[\varphi_{492}(r)]^2$	-4%d_{z^2}(Ru), -3%$d_{x^2-y^2}$(Ru), 3%p_y(C), 5%p_z(C)
**7**	1	-60.55	0.61592	$0.616[\varphi_1(r)]^2 - 0.616[\varphi_{547}(r)]^2$	8%$d_{x^2-y^2}$(Ru), -4%s(C), -4%p_y(C), -20%s(C)
	2	-26.93	0.55334	$0.553[\varphi_2(r)]^2 - 0.553[\varphi_{546}(r)]^2$	-13%d_{xy}(Ru), 9%p_x(C)
	3	-25.51	0.53281	$0.533[\varphi_3(r)]^2 - 0.533[\varphi_{545}(r)]^2$	-7%d_{yz}(Ru), 8%p_z(C)
**8**	1	-49.34	0.56559	$0.566[\varphi_1(r)]^2 - 0.566[\varphi_{428}(r)]^2$	3%d_{z^2}(Ru), 3%d_{xz}(Ru), -3%s(C), -19%s(C)
	2	-31.97	0.55653	$0.557[\varphi_2(r)]^2 - 0.557[\varphi_{427}(r)]^2$	-16%d_{xz}(Ru), 8%p_x(C)
	3	-25.22	0.53641	$0.536[\varphi_3(r)]^2 - 0.536[\varphi_{426}(r)]^2$	-9%d_{yz}(Ru), 9%p_y(C)
**9**	1	-70.35	0.67297	$0.673[\varphi_1(r)]^2 - 0.673[\varphi_{442}(r)]^2$	3%s(Ru), 3%d_{z^2}(Ru), 5%d_{xz}(Ru), 9%d_{yz}(Ru), -3%s(C), -26%s(C)
	2	-34.09	0.60692	$0.607[\varphi_2(r)]^2 - 0.607[\varphi_{441}(r)]^2$	-18%d_{xz}(Ru), 9%p_x(C)
	3	-33.88	0.57605	$0.576[\varphi_3(r)]^2 - 0.576[\varphi_{440}(r)]^2$	-4%d_{xz}(Ru), -12%d_{yz}(Ru), 9%p_y(C)
**10**	1	-78.95	0.69940	$0.699[\varphi_1(r)]^2 - 0.699[\varphi_{456}(r)]^2$	3%s(Ru), 4%d_{z^2}(Ru), 4%d_{xz}(Ru), 10%d_{yz}(Ru), 4%$d_{x^2-y^2}$(Ru), -3%s(C), -27%s(C)
	2	-37.12	0.59858	$0.599[\varphi_2(r)]^2 - 0.599[\varphi_{455}(r)]^2$	-14%d_{xz}(Ru), -7%d_{xy}(Ru), 10%p_x(C)
	3	-32.54	0.58542	$0.585[\varphi_3(r)]^2 - 0.585[\varphi_{454}(r)]^2$	-9%d_{z^2}(Ru), -4%$d_{x^2-y^2}$(Ru), 6%p_y(C), 4%p_z(C)
**11**	1	-55.33	0.57489	$0.575[\varphi_1(r)]^2 - 0.575[\varphi_{433}(r)]^2$	9%d_{z^2}(Ru), -3%s(C), -24%s(C)
	2	-23.40	0.51692	$0.517[\varphi_2(r)]^2 - 0.517[\varphi_{432}(r)]^2$	-12%d_{xz}(Ru), 8%p_x(C)
	3	-22.07	0.49461	$0.495[\varphi_3(r)]^2 - 0.495[\varphi_{431}(r)]^2$	-8%d_{yz}(Ru), 8%p_y(C)
**12**	1	-27.26	0.55532	$0.555[\varphi_1(r)]^2 - 0.555[\varphi_{490}(r)]^2$	-14%d_{xy}(Ru), 9%p_x(C)
	2	-48.36	0.54411	$0.544[\varphi_2(r)]^2 - 0.544[\varphi_{489}(r)]^2$	6%$d_{x^2-y^2}$(Ru), -3%p_y(C), -21%s(C)
	3	-26.73	0.53088	$0.531[\varphi_3(r)]^2 - 0.531[\varphi_{488}(r)]^2$	-6%d_{z^2}(Ru), -5%$d_{x^2-y^2}$(Ru), 5%p_y(C)
**13**	1	-56.50	0.60057	$0.601[\varphi_1(r)]^2 - 0.601[\varphi_{475}(r)]^2$	6%d_{z^2}(Ru), 4%d_{yz}(Ru), -3%s(C), -3%p_z(C), -19%s(C)
	2	-25.39	0.52701	$0.527[\varphi_2(r)]^2 - 0.527[\varphi_{474}(r)]^2$	-6%d_{xz}(Ru), 7%p_x(C)
	3	-23.65	0.50721	$0.507[\varphi_3(r)]^2 - 0.507[\varphi_{473}(r)]^2$	-8%d_{yz}(Ru), 8%p_y(C)
**14**	1	-62.88	0.63353	$0.634[\varphi_1(r)]^2 - 0.634[\varphi_{460}(r)]^2$	5%d_{z^2}(Ru), 4%d_{yz}(Ru), -3%s(C), -22%s(C)
	2	-23.64	0.51475	$0.515[\varphi_2(r)]^2 - 0.515[\varphi_{459}(r)]^2$	-7%d_{xz}(Ru), 9%p_x(C)
	3	-22.29	0.49115	$0.491[\varphi_3(r)]^2 - 0.491[\varphi_{458}(r)]^2$	-5%d_{yz}(Ru), 8%p_y(C)
**15**	1	-67.16	0.65008	$0.650[\varphi_1(r)]^2 - 0.650[\varphi_{433}(r)]^2$	12%d_{z^2}(Ru), -3%s(C), -3%p_z(C), -22%s(C)
	2	-28.67	0.56233	$0.562[\varphi_2(r)]^2 - 0.562[\varphi_{432}(r)]^2$	-15%d_{xz}(Ru), 10%p_x(C)
	3	-26.61	0.53912	$0.539[\varphi_3(r)]^2 - 0.539[\varphi_{431}(r)]^2$	-9%d_{yz}(Ru), 9%p_y(C)

[a] Positive and negative percentages refer to the positive and negative part of the NOCV pair; C refers to carbon atom of the CO ligand.

Table S7. Cartesian coordinates and energetic data.

$[Ru(salen)(CO)Cl]^{-1}$ S_0

Ru	0.007186106	0.064651167	0.108835846
C	0.210978054	-0.037686664	1.914369714
O	0.345324334	-0.115560006	3.066897394
Cl	-0.281650397	0.210214125	-2.448003032
N	1.328597376	1.580632244	-0.080948928
O	1.515236520	-1.372180859	-0.170218176
C	3.354359531	0.234363372	-0.066913678
C	2.775703653	-1.087341330	-0.102642732
C	3.695673898	-2.177195919	-0.082340500
C	5.060700010	-1.996732554	-0.027188911
C	5.617062137	-0.705887233	0.007489135
C	4.761606807	0.374437476	-0.019230346
C	2.609934345	1.467037611	-0.131055903
C	0.656605020	2.859777191	-0.294512387
N	-1.327730250	1.572805681	0.257318250
O	-1.503709970	-1.387894169	0.103273810
C	-3.345640214	0.221926415	0.101651868
C	-2.763456063	-1.098152405	0.063946042
C	-3.681436522	-2.186456462	-0.028238785
C	-5.046532152	-2.006434744	-0.081275073
C	-5.606215538	-0.717204483	-0.044801203
C	-4.752732176	0.361145043	0.046737720
C	-2.608250900	1.455572769	0.208163073
C	-0.670094372	2.859983862	0.461345955
H	3.267732448	-3.175697131	-0.111499215
H	5.712632818	-2.867142651	-0.011642078
H	6.691989832	-0.563425172	0.048328034
H	5.170033031	1.383325739	-0.004004761
H	3.201080996	2.381838159	-0.242638919
H	1.284144069	3.702220180	0.017340437
H	0.453644698	2.949327322	-1.368219618
H	-3.251203025	-3.183980919	-0.056703076
H	-5.695701528	-2.876165594	-0.152583359
H	-6.681087140	-0.574477483	-0.085675249
H	-5.163237670	1.368786621	0.079709291
H	-3.207554914	2.370355230	0.259713304
H	-1.301919371	3.692409086	0.132047354
H	-0.479156174	2.978438268	1.535868749

Sum of electronic and zero-point Energies= -1544.209541
Sum of electronic and thermal Energies= -1544.188756
Sum of electronic and thermal Enthalpies= -1544.187812
Sum of electronic and thermal Free Energies= -1544.259563

$[Ru(salen)(CO)Cl]^{-1}$ T_1

Ru	0.048140000	0.052930000	-0.061808000
C	0.678090000	-0.146973000	1.671580000
O	1.067656000	-0.294020000	2.746119000
Cl	-0.835643000	0.240385000	-2.416851000
N	1.273900000	1.609262000	-0.352299000
O	1.499812000	-1.134723000	-0.785905000
C	3.312545000	0.306587000	-0.006910000
C	2.762481000	-0.983852000	-0.389391000
C	3.597441000	-2.108132000	-0.405132000
C	4.941353000	-2.041046000	-0.041973000
C	5.484340000	-0.803243000	0.343263000
C	4.695183000	0.327810000	0.356239000
C	2.589126000	1.515136000	-0.018748000

C	0.582635000	2.879066000	-0.367722000
N	-1.274851000	1.544821000	0.416686000
O	-1.451212000	-1.343806000	0.332242000
C	-3.310904000	0.219090000	0.322255000
C	-2.724705000	-1.090933000	0.278018000
C	-3.618678000	-2.192994000	0.213194000
C	-4.987243000	-2.022345000	0.174666000
C	-5.556966000	-0.738613000	0.215939000
C	-4.717308000	0.352103000	0.298441000
C	-2.557399000	1.437787000	0.447933000
C	-0.595157000	2.820178000	0.611618000
H	3.150518000	-3.052040000	-0.708356000
H	5.556392000	-2.936021000	-0.056277000
H	6.530855000	-0.731961000	0.629006000
H	5.126600000	1.283544000	0.647679000
H	3.121804000	2.441396000	0.202627000
H	1.253789000	3.702315000	-0.093715000
H	0.190972000	3.076847000	-1.375104000
H	-3.178938000	-3.185915000	0.188663000
H	-5.630600000	-2.896715000	0.115140000
H	-6.633835000	-0.608803000	0.191303000
H	-5.136439000	1.355094000	0.344378000
H	-3.139495000	2.352681000	0.597112000
H	-1.285871000	3.660515000	0.474596000
H	-0.210397000	2.847663000	1.638991000

Sum of electronic and zero-point Energies= -1544.133868
Sum of electronic and thermal Energies= -1544.112720
Sum of electronic and thermal Enthalpies= -1544.111776
Sum of electronic and thermal Free Energies= -1544.184796

[Ru(salen)Cl]$^{-1}$ S_0

Ru	-0.018191000	0.074506000	-0.095430000
N	-1.349718000	1.555920000	-0.104396000
O	-1.532610000	-1.372852000	-0.055989000
C	-3.380845000	0.218498000	-0.191246000
C	-2.796801000	-1.101413000	-0.139763000
C	-3.709193000	-2.196283000	-0.194318000
C	-5.074816000	-2.026378000	-0.284981000
C	-5.635802000	-0.738670000	-0.329419000
C	-4.785919000	0.347467000	-0.282660000
C	-2.638276000	1.454689000	-0.155790000
C	-0.701636000	2.856474000	0.048604000
N	1.299027000	1.543353000	-0.401633000
O	1.485168000	-1.378638000	-0.251775000
C	3.334682000	0.212632000	-0.317374000
C	2.750416000	-1.105954000	-0.241647000
C	3.667354000	-2.196155000	-0.165826000
C	5.035582000	-2.021690000	-0.154818000
C	5.595505000	-0.734883000	-0.225669000
C	4.741667000	0.346318000	-0.311081000
C	2.586645000	1.439543000	-0.448529000
C	0.644728000	2.827100000	-0.669105000
H	-3.275350000	-3.192443000	-0.158282000
H	-5.720803000	-2.900785000	-0.320962000
H	-6.710128000	-0.600747000	-0.399380000
H	-5.200178000	1.353917000	-0.316931000
H	-3.229788000	2.375724000	-0.175752000
H	-1.327649000	3.672034000	-0.332744000
H	-0.535029000	3.018095000	1.120797000

H	3.234991000	-3.192171000	-0.111526000
H	5.684566000	-2.892278000	-0.089952000
H	6.671700000	-0.593938000	-0.218968000
H	5.154802000	1.351704000	-0.377041000
H	3.174054000	2.347182000	-0.622381000
H	1.270528000	3.670917000	-0.355418000
H	0.481564000	2.912780000	-1.751198000
Cl	0.238926000	0.248293000	2.276161000

Sum of electronic and zero-point Energies= -1430.955626
Sum of electronic and thermal Energies= -1430.936957
Sum of electronic and thermal Enthalpies= -1430.936013
Sum of electronic and thermal Free Energies= -1431.003270

$[Ru(salen)Cl]^{-1}$ T_1

Ru	-0.008996000	0.068934000	-0.075949000
N	-1.337356000	1.558282000	-0.185701000
O	-1.497321000	-1.358959000	-0.040047000
C	-3.357870000	0.203933000	-0.267832000
C	-2.765607000	-1.106306000	-0.170914000
C	-3.656196000	-2.215902000	-0.222586000
C	-5.020361000	-2.063266000	-0.342581000
C	-5.594993000	-0.781888000	-0.427076000
C	-4.764510000	0.316258000	-0.390587000
C	-2.626418000	1.440259000	-0.258029000
C	-0.704472000	2.872081000	-0.084987000
N	1.300642000	1.541654000	-0.440270000
O	1.472747000	-1.362220000	-0.177027000
C	3.332916000	0.202179000	-0.365764000
C	2.745078000	-1.106494000	-0.230448000
C	3.644209000	-2.208454000	-0.163109000
C	5.011907000	-2.047958000	-0.205388000
C	5.581842000	-0.767567000	-0.331634000
C	4.743573000	0.321731000	-0.416116000
C	2.590230000	1.421653000	-0.516651000
C	0.654784000	2.815097000	-0.771940000
H	-3.209551000	-3.204203000	-0.155055000
H	-5.656609000	-2.944553000	-0.370646000
H	-6.669455000	-0.661031000	-0.520332000
H	-5.189950000	1.315735000	-0.457655000
H	-3.223528000	2.355270000	-0.320130000
H	-1.329465000	3.659104000	-0.522846000
H	-0.565730000	3.089898000	0.980593000
H	3.201134000	-3.196066000	-0.068036000
H	5.655090000	-2.922319000	-0.141636000
H	6.659152000	-0.641705000	-0.367943000
H	5.165101000	1.319036000	-0.526027000
H	3.177521000	2.318678000	-0.736910000
H	1.276242000	3.668036000	-0.475945000
H	0.518183000	2.856183000	-1.859741000
Cl	0.126858000	0.367797000	2.705666000

Sum of electronic and zero-point Energies= -1430.961272
Sum of electronic and thermal Energies= -1430.942251
Sum of electronic and thermal Enthalpies= -1430.941307
Sum of electronic and thermal Free Energies= -1431.010537

$[Ru(salen)(H_2O)Cl]^{-1}$ S_0

Ru	-0.009316000	0.074987000	0.015740000
N	-1.339212000	1.556420000	-0.000496000
O	-1.539870000	-1.371784000	0.173960000

C	-3.370441000	0.219075000	-0.112290000
C	-2.792745000	-1.100571000	0.020430000
C	-3.713512000	-2.192153000	-0.021566000
C	-5.072647000	-2.020307000	-0.177702000
C	-5.624464000	-0.733615000	-0.303901000
C	-4.769681000	0.349348000	-0.266493000
C	-2.626585000	1.455393000	-0.076227000
C	-0.692200000	2.855826000	0.159307000
N	1.307801000	1.541742000	-0.290457000
O	1.511892000	-1.383256000	-0.075055000
C	3.350132000	0.213443000	-0.238735000
C	2.772807000	-1.106237000	-0.116145000
C	3.699431000	-2.190707000	-0.047374000
C	5.066112000	-2.011248000	-0.090398000
C	5.618039000	-0.724163000	-0.209151000
C	4.756135000	0.351453000	-0.284234000
C	2.595214000	1.437471000	-0.359107000
C	0.655394000	2.826314000	-0.555716000
H	-3.288860000	-3.188190000	0.077530000
H	-5.721793000	-2.893014000	-0.202308000
H	-6.694109000	-0.594238000	-0.424656000
H	-5.176359000	1.355626000	-0.357256000
H	-3.216967000	2.377084000	-0.109626000
H	-1.316695000	3.673763000	-0.220347000
H	-0.526850000	3.011805000	1.232347000
H	3.274192000	-3.187345000	0.042724000
H	5.720637000	-2.878287000	-0.031842000
H	6.693123000	-0.579035000	-0.245274000
H	5.162348000	1.357085000	-0.383627000
H	3.178734000	2.346788000	-0.538187000
H	1.281438000	3.669510000	-0.240696000
H	0.493684000	2.916487000	-1.638333000
O	-0.223488000	-0.181936000	-2.148212000
H	-0.830334000	0.489172000	-2.486056000
H	0.629083000	0.015396000	-2.556188000
Cl	0.227901000	0.284747000	2.464482000

Sum of electronic and zero-point Energies= -1507.300323
Sum of electronic and thermal Energies= -1507.278955
Sum of electronic and thermal Enthalpies= -1507.278011
Sum of electronic and thermal Free Energies= -1507.350357

[Ru(salen)(CO)F]$^{-1}$ S$_0$

Ru	-0.005764000	0.079692000	-0.005142000
C	0.193919000	-0.011389000	1.824186000
O	0.328151000	-0.075654000	2.981885000
F	-0.210937000	0.164507000	-2.007748000
N	1.314080000	1.581856000	-0.220511000
O	1.501566000	-1.362530000	-0.294073000
C	3.340336000	0.240309000	-0.167707000
C	2.759361000	-1.081447000	-0.203579000
C	3.680601000	-2.170951000	-0.159321000
C	5.044645000	-1.991203000	-0.080559000
C	5.601224000	-0.700373000	-0.046462000
C	4.746154000	0.379972000	-0.098292000
C	2.595709000	1.470922000	-0.268082000
C	0.639929000	2.847890000	-0.490666000
N	-1.338577000	1.580920000	0.103464000
O	-1.518714000	-1.377146000	-0.037691000
C	-3.359374000	0.231683000	-0.036061000

C	-2.776927000	-1.089711000	-0.065210000
C	-3.699095000	-2.177342000	-0.141704000
C	-5.064495000	-1.997081000	-0.182395000
C	-5.622718000	-0.706891000	-0.152610000
C	-4.766457000	0.371257000	-0.081350000
C	-2.618952000	1.466143000	0.041485000
C	-0.684601000	2.874799000	0.267422000
H	3.253314000	-3.169893000	-0.189429000
H	5.695716000	-2.861897000	-0.046513000
H	6.675435000	-0.557831000	0.011757000
H	5.154720000	1.389067000	-0.086758000
H	3.185855000	2.382919000	-0.407187000
H	1.265203000	3.707169000	-0.222346000
H	0.432571000	2.887072000	-1.566569000
H	-3.270434000	-3.175847000	-0.165647000
H	-5.715139000	-2.866929000	-0.239216000
H	-6.697838000	-0.563106000	-0.184290000
H	-5.175609000	1.379876000	-0.056830000
H	-3.216777000	2.383401000	0.061577000
H	-1.317422000	3.696872000	-0.085898000
H	-0.489649000	3.025814000	1.337105000

Sum of electronic and zero-point Energies= -1183.887728
Sum of electronic and thermal Energies= -1183.867463
Sum of electronic and thermal Enthalpies= -1183.866519
Sum of electronic and thermal Free Energies= -1183.936760

[Ru(salen)(CO)F]$^{-1}$ T$_1$

Ru	-0.036238000	0.087609000	0.057123000
C	0.560546000	0.118782000	1.851146000
O	1.000103000	0.126634000	2.916744000
F	-0.510133000	-0.002859000	-1.862470000
N	1.248743000	1.591205000	-0.290896000
O	1.401431000	-1.296998000	-0.374722000
C	3.289890000	0.246054000	-0.276525000
C	2.692257000	-1.073522000	-0.291259000
C	3.553828000	-2.190211000	-0.273235000
C	4.935502000	-2.064743000	-0.223056000
C	5.520618000	-0.786743000	-0.206922000
C	4.711945000	0.329153000	-0.240050000
C	2.563857000	1.461953000	-0.373926000
C	0.572762000	2.841863000	-0.581523000
N	-1.341819000	1.595905000	0.173440000
O	-1.507784000	-1.247441000	0.457808000
C	-3.360423000	0.250706000	-0.062208000
C	-2.773211000	-1.050011000	0.164179000
C	-3.617244000	-2.178534000	0.118807000
C	-4.974573000	-2.078012000	-0.151767000
C	-5.551209000	-0.814550000	-0.370843000
C	-4.758752000	0.312469000	-0.317637000
C	-2.644828000	1.474277000	0.006001000
C	-0.691271000	2.889533000	0.274560000
H	3.085959000	-3.171313000	-0.295592000
H	5.558795000	-2.954509000	-0.198433000
H	6.600830000	-0.676532000	-0.172028000
H	5.160583000	1.320739000	-0.236634000
H	3.143126000	2.374490000	-0.533466000
H	1.219178000	3.704058000	-0.378852000
H	0.290587000	2.864651000	-1.644974000
H	-3.156675000	-3.146013000	0.301700000

H	-5.586787000	-2.974745000	-0.189811000
H	-6.613982000	-0.724006000	-0.576599000
H	-5.202475000	1.293015000	-0.478517000
H	-3.226147000	2.395372000	-0.078987000
H	-1.357779000	3.699550000	-0.042879000
H	-0.406175000	3.068038000	1.322979000

Sum of electronic and zero-point Energies= -1183.805067
Sum of electronic and thermal Energies= -1183.784413
Sum of electronic and thermal Enthalpies= -1183.783469
Sum of electronic and thermal Free Energies= -1183.855410

$[Ru(salen)F]^{-1}$ S_0

Ru	-0.007498000	0.091065000	-0.019015000
F	0.192265000	0.139619000	1.934959000
N	-1.332908000	1.564487000	0.033106000
O	-1.521455000	-1.362786000	0.023648000
C	-3.368235000	0.233395000	-0.075095000
C	-2.785107000	-1.089034000	-0.052419000
C	-3.700446000	-2.180939000	-0.122607000
C	-5.066759000	-2.009113000	-0.204352000
C	-5.625692000	-0.720088000	-0.223765000
C	-4.773572000	0.364038000	-0.159227000
C	-2.624667000	1.467624000	-0.005275000
C	-0.687998000	2.860677000	0.233521000
N	1.308850000	1.560543000	-0.247168000
O	1.500756000	-1.365603000	-0.157767000
C	3.347788000	0.231306000	-0.175252000
C	2.764848000	-1.090857000	-0.133663000
C	3.684681000	-2.179702000	-0.066483000
C	5.052929000	-2.004280000	-0.037676000
C	5.610574000	-0.715505000	-0.079690000
C	4.754500000	0.365546000	-0.151979000
C	2.599782000	1.461572000	-0.274938000
C	0.662007000	2.855254000	-0.476654000
H	-3.267620000	-3.178245000	-0.105781000
H	-5.713741000	-2.882284000	-0.252956000
H	-6.700126000	-0.578946000	-0.287255000
H	-5.187267000	1.371402000	-0.171557000
H	-3.214881000	2.389426000	0.014553000
H	-1.313340000	3.688213000	-0.122988000
H	-0.527642000	2.990524000	1.311362000
H	3.253711000	-3.177464000	-0.034281000
H	5.702560000	-2.875057000	0.019086000
H	6.686362000	-0.571913000	-0.059352000
H	5.166634000	1.372885000	-0.192058000
H	3.187739000	2.375920000	-0.405339000
H	1.288005000	3.686360000	-0.130468000
H	0.505351000	2.978302000	-1.556234000

Sum of electronic and zero-point Energies= -1070.632788
Sum of electronic and thermal Energies= -1070.614491
Sum of electronic and thermal Enthalpies= -1070.613546
Sum of electronic and thermal Free Energies= -1070.679342

$[Ru(salen)F]^{-1}$ T_1

Ru	-0.000640000	0.096302000	0.125161000
F	0.095951000	0.172371000	2.261159000
N	-1.331785000	1.576303000	0.024616000
O	-1.494124000	-1.342028000	0.094308000
C	-3.350040000	0.224938000	-0.142234000

C	-2.756701000	-1.087698000	-0.069977000
C	-3.644918000	-2.194750000	-0.191130000
C	-5.005769000	-2.041077000	-0.347695000
C	-5.580278000	-0.758406000	-0.404401000
C	-4.752073000	0.338493000	-0.304819000
C	-2.619941000	1.461537000	-0.082606000
C	-0.705642000	2.890920000	0.146789000
N	1.300191000	1.570459000	-0.236565000
O	1.484317000	-1.338231000	-0.018669000
C	3.336515000	0.231888000	-0.237575000
C	2.752290000	-1.080648000	-0.109233000
C	3.656360000	-2.181838000	-0.091027000
C	5.022614000	-2.020052000	-0.167890000
C	5.587720000	-0.736572000	-0.284174000
C	4.745210000	0.352630000	-0.324243000
C	2.589362000	1.453418000	-0.347034000
C	0.654227000	2.850689000	-0.539304000
H	-3.198427000	-3.184662000	-0.145840000
H	-5.638271000	-2.922106000	-0.427339000
H	-6.651600000	-0.634678000	-0.527043000
H	-5.177309000	1.339459000	-0.352459000
H	-3.214766000	2.377938000	-0.148385000
H	-1.333216000	3.682347000	-0.280375000
H	-0.570264000	3.099152000	1.215147000
H	3.217142000	-3.172093000	-0.003114000
H	5.667626000	-2.895113000	-0.139396000
H	6.663568000	-0.607743000	-0.347395000
H	5.162922000	1.352627000	-0.426449000
H	3.170386000	2.354313000	-0.568867000
H	1.274946000	3.697083000	-0.222187000
H	0.517859000	2.918750000	-1.625950000

Sum of electronic and zero-point Energies= -1070.624897
Sum of electronic and thermal Energies= -1070.606303
Sum of electronic and thermal Enthalpies= -1070.605359
Sum of electronic and thermal Free Energies= -1070.672820

[Ru(salen)(H_2O)F]$^{-1}$ S_0

Ru	-0.003901000	0.088395000	-0.102515000
F	-0.208487000	0.089609000	-2.087367000
N	1.319268000	1.556514000	-0.179315000
O	1.520640000	-1.370786000	-0.211574000
C	3.355894000	0.228760000	-0.023831000
C	2.776760000	-1.095851000	-0.091042000
C	3.698484000	-2.184548000	-0.019019000
C	5.060508000	-2.008686000	0.111052000
C	5.612763000	-0.718208000	0.178077000
C	4.756909000	0.363152000	0.107605000
C	2.610852000	1.461946000	-0.110894000
C	0.678475000	2.852452000	-0.392463000
N	-1.317000000	1.556259000	0.106926000
O	-1.521975000	-1.372215000	0.020918000
C	-3.362057000	0.230300000	0.087422000
C	-2.783133000	-1.094411000	0.029708000
C	-3.710397000	-2.179626000	-0.017418000
C	-5.078314000	-1.999947000	-0.007457000
C	-5.630645000	-0.709401000	0.052929000
C	-4.768529000	0.368436000	0.102125000
C	-2.608668000	1.458863000	0.160003000
C	-0.670749000	2.853801000	0.318730000

H	3.272261000	-3.183613000	-0.072076000
H	5.709835000	-2.880236000	0.161119000
H	6.683919000	-0.573762000	0.279131000
H	5.165285000	1.372048000	0.150903000
H	3.199801000	2.384832000	-0.125453000
H	1.305230000	3.681705000	-0.041607000
H	0.518988000	2.975519000	-1.471058000
H	-3.284255000	-3.179121000	-0.062815000
H	-5.732070000	-2.868723000	-0.047295000
H	-6.706118000	-0.561984000	0.063360000
H	-5.176104000	1.377309000	0.153362000
H	-3.192445000	2.376705000	0.285325000
H	-1.298050000	3.681464000	-0.033442000
H	-0.510222000	2.990821000	1.396694000
O	0.251391000	-0.008610000	2.115071000
H	-0.530911000	0.385316000	2.520081000
H	0.977641000	0.587145000	2.338986000

Sum of electronic and zero-point Energies= -1146.975975
Sum of electronic and thermal Energies= -1146.955006
Sum of electronic and thermal Enthalpies= -1146.954062
Sum of electronic and thermal Free Energies= -1147.025156

[Ru(salen)(CO)SCN]$^{-1}$ S_0

Ru	-0.058663000	-0.122186000	0.278656000
C	0.089789000	-0.916507000	1.919474000
O	0.191162000	-1.435055000	2.953292000
S	-0.401138000	0.834528000	-2.114649000
N	1.291508000	1.315319000	0.717874000
O	1.433303000	-1.383027000	-0.497230000
C	3.294821000	0.020912000	0.235208000
C	2.696818000	-1.173610000	-0.310068000
C	3.598340000	-2.209021000	-0.695752000
C	4.964638000	-2.093424000	-0.558542000
C	5.540462000	-0.926166000	-0.026618000
C	4.702997000	0.101221000	0.350596000
C	2.572061000	1.198675000	0.642878000
C	0.653168000	2.593451000	1.029110000
N	-1.372296000	1.251353000	0.967869000
O	-1.600753000	-1.398501000	-0.353321000
C	-3.410578000	0.137919000	0.240513000
C	-2.855443000	-1.078046000	-0.301242000
C	-3.789178000	-2.013564000	-0.834896000
C	-5.148030000	-1.783571000	-0.845280000
C	-5.682483000	-0.595296000	-0.317841000
C	-4.811846000	0.332884000	0.211248000
C	-2.652607000	1.204567000	0.844970000
C	-0.699449000	2.330303000	1.685728000
C	0.771793000	1.999383000	-2.343883000
N	1.588840000	2.825679000	-2.512257000
H	3.155361000	-3.111169000	-1.108946000
H	5.602379000	-2.918248000	-0.867969000
H	6.616421000	-0.834187000	0.079253000
H	5.126740000	1.018316000	0.755741000
H	3.180189000	2.069334000	0.907350000
H	1.286266000	3.208899000	1.677937000
H	0.504766000	3.129366000	0.083934000
H	-3.377650000	-2.932550000	-1.243256000
H	-5.811569000	-2.534412000	-1.267984000
H	-6.752233000	-0.413991000	-0.324788000

H	-5.203123000	1.258583000	0.629223000
H	-3.234860000	2.043253000	1.239578000
H	-1.305471000	3.242873000	1.701318000
H	-0.545566000	2.003221000	2.722162000

Sum of electronic and zero-point Energies= -1574.925600
Sum of electronic and thermal Energies= -1574.902541
Sum of electronic and thermal Enthalpies= -1574.901597
Sum of electronic and thermal Free Energies= -1574.979156

[Ru(salen)(CO)SCN]$^{-1}$ T_1

Ru	0.085367000	0.237198000	-0.368452000
C	-0.525157000	0.870297000	-2.014206000
O	-0.942797000	1.243864000	-3.020294000
S	0.839708000	-0.644556000	1.905668000
N	-1.211451000	1.505407000	0.573083000
O	-1.385261000	-1.233712000	-0.378499000
C	-3.244110000	0.258851000	0.103798000
C	-2.659081000	-0.976906000	-0.341301000
C	-3.555582000	-2.007385000	-0.732775000
C	-4.923397000	-1.833710000	-0.711429000
C	-5.491850000	-0.622716000	-0.279513000
C	-4.651894000	0.390542000	0.128885000
C	-2.492753000	1.373833000	0.616604000
C	-0.517530000	2.632019000	1.189240000
N	1.362567000	1.743348000	-0.057163000
O	1.568592000	-0.801827000	-1.261247000
C	3.344506000	0.307235000	0.005993000
C	2.804696000	-0.795894000	-0.776192000
C	3.628824000	-1.886836000	-1.086097000
C	4.947800000	-1.971911000	-0.647718000
C	5.477954000	-0.921029000	0.123033000
C	4.704007000	0.176459000	0.433052000
C	2.644403000	1.485510000	0.319432000
C	0.695768000	2.971073000	0.314831000
C	-0.553015000	-1.310799000	2.554079000
N	-1.499160000	-1.783171000	3.060389000
H	-3.116570000	-2.945477000	-1.059954000
H	-5.568147000	-2.648756000	-1.030644000
H	-6.568712000	-0.492312000	-0.258855000
H	-5.070482000	1.329772000	0.484339000
H	-3.070606000	2.170699000	1.094432000
H	-1.190526000	3.488086000	1.316858000
H	-0.163651000	2.305615000	2.174181000
H	3.188292000	-2.681888000	-1.682993000
H	5.554194000	-2.837017000	-0.898449000
H	6.506115000	-0.968102000	0.473355000
H	5.128929000	0.987150000	1.021606000
H	3.171864000	2.268843000	0.865268000
H	1.369845000	3.644758000	0.857039000
H	0.348476000	3.497306000	-0.587108000

Sum of electronic and zero-point Energies= -1574.849665
Sum of electronic and thermal Energies= -1574.826196
Sum of electronic and thermal Enthalpies= -1574.825252
Sum of electronic and thermal Free Energies= -1574.904245

[Ru(salen)SCN]$^{-1}$ S_0

Ru	0.062436000	-0.049645000	-0.267388000
N	-1.295491000	1.372188000	-0.619401000
O	-1.420974000	-1.497086000	0.004821000

C	-3.294617000	-0.009664000	-0.489402000
C	-2.687440000	-1.277379000	-0.158873000
C	-3.576418000	-2.383092000	-0.014166000
C	-4.941501000	-2.267700000	-0.170126000
C	-5.525833000	-1.028967000	-0.486026000
C	-4.699039000	0.064577000	-0.638762000
C	-2.578140000	1.225109000	-0.690345000
C	-0.681069000	2.693603000	-0.729195000
N	1.360069000	1.364460000	-0.824182000
O	1.599753000	-1.466552000	-0.093526000
C	3.417872000	0.132019000	-0.409372000
C	2.860419000	-1.162463000	-0.099706000
C	3.793888000	-2.193790000	0.210515000
C	5.157115000	-1.983764000	0.230345000
C	5.692372000	-0.719306000	-0.067086000
C	4.820884000	0.302668000	-0.384655000
C	2.650476000	1.287658000	-0.807255000
C	0.693215000	2.547746000	-1.375429000
H	-3.124773000	-3.341015000	0.230940000
H	-5.569543000	-3.146946000	-0.045391000
H	-6.600173000	-0.934482000	-0.607771000
H	-5.131017000	1.033172000	-0.885270000
H	-3.187160000	2.104819000	-0.921949000
H	-1.307001000	3.386821000	-1.303527000
H	-0.570429000	3.093196000	0.286731000
H	3.379542000	-3.172090000	0.440430000
H	5.821026000	-2.808533000	0.479427000
H	6.764533000	-0.551060000	-0.054324000
H	5.215579000	1.287235000	-0.630454000
H	3.226044000	2.156378000	-1.143102000
H	1.292336000	3.453252000	-1.224301000
H	0.571451000	2.400945000	-2.456269000
S	0.359219000	0.435134000	1.991254000
C	-0.903122000	1.427756000	2.489864000
N	-1.764531000	2.126152000	2.870370000

Sum of electronic and zero-point Energies= -1461.681209
Sum of electronic and thermal Energies= -1461.660288
Sum of electronic and thermal Enthalpies= -1461.659344
Sum of electronic and thermal Free Energies= -1461.732451

$[Ru(salen)SCN]^{-1}$ T_1

Ru	-0.012402000	0.255638000	-0.205237000
N	-1.340022000	1.686735000	0.232072000
O	-1.500068000	-1.089270000	-0.681587000
C	-3.360103000	0.451115000	-0.329356000
C	-2.768306000	-0.807422000	-0.707886000
C	-3.659907000	-1.826604000	-1.147834000
C	-5.024063000	-1.641555000	-1.200911000
C	-5.598279000	-0.414093000	-0.822088000
C	-4.767316000	0.599326000	-0.399558000
C	-2.629191000	1.602406000	0.120225000
C	-0.707885000	2.879507000	0.794327000
N	1.298975000	1.760004000	-0.006885000
O	1.465628000	-1.038217000	-0.825247000
C	3.328759000	0.481904000	-0.421209000
C	2.738450000	-0.783841000	-0.775798000
C	3.635402000	-1.836417000	-1.114034000
C	5.003402000	-1.675209000	-1.091271000
C	5.575852000	-0.438926000	-0.738990000

C	4.739822000	0.607993000	-0.420963000
C	2.588398000	1.674706000	-0.121308000
C	0.653618000	3.068600000	0.135790000
H	-3.213866000	-2.773934000	-1.438135000
H	-5.661144000	-2.455257000	-1.538901000
H	-6.672940000	-0.268716000	-0.862816000
H	-5.192160000	1.556839000	-0.104498000
H	-3.226616000	2.479193000	0.388229000
H	-1.331696000	3.770867000	0.661073000
H	-0.575478000	2.711802000	1.870617000
H	3.190306000	-2.789007000	-1.388055000
H	5.644966000	-2.513869000	-1.350591000
H	6.653463000	-0.311452000	-0.724373000
H	5.163250000	1.575333000	-0.157807000
H	3.176381000	2.590134000	-0.002644000
H	1.274686000	3.760226000	0.716392000
H	0.518380000	3.493340000	-0.866600000
S	0.175441000	-0.381417000	2.485524000
C	0.051981000	-2.041169000	2.577629000
N	-0.035692000	-3.211445000	2.645413000

Sum of electronic and zero-point Energies= -1461.678499
Sum of electronic and thermal Energies= -1461.657038
Sum of electronic and thermal Enthalpies= -1461.656094
Sum of electronic and thermal Free Energies= -1461.732704

[Ru(salen)(H_2O)SCN]$^{-1}$ S_0

Ru	0.055648000	-0.055030000	-0.187325000
N	-1.298950000	1.345507000	-0.622346000
O	-1.450357000	-1.444960000	0.312970000
C	-3.305578000	-0.012551000	-0.373592000
C	-2.708402000	-1.238648000	0.107847000
C	-3.612812000	-2.312142000	0.372517000
C	-4.974350000	-2.207195000	0.184602000
C	-5.545438000	-1.011599000	-0.285536000
C	-4.707197000	0.051693000	-0.550258000
C	-2.582294000	1.197769000	-0.680034000
C	-0.681019000	2.652155000	-0.838834000
N	1.353898000	1.312154000	-0.845293000
O	1.607760000	-1.444629000	0.155765000
C	3.416629000	0.120706000	-0.333500000
C	2.864311000	-1.140241000	0.104235000
C	3.807458000	-2.134481000	0.501693000
C	5.170096000	-1.921971000	0.483148000
C	5.698939000	-0.691339000	0.058418000
C	4.819516000	0.294251000	-0.342011000
C	2.644461000	1.239127000	-0.819486000
C	0.686963000	2.449443000	-1.483218000
H	-3.172870000	-3.237582000	0.735761000
H	-5.610791000	-3.061803000	0.403829000
H	-6.617238000	-0.925299000	-0.433140000
H	-5.128694000	0.989243000	-0.909875000
H	-3.188348000	2.059984000	-0.976968000
H	-1.309567000	3.301029000	-1.460648000
H	-0.560229000	3.128678000	0.141725000
H	3.399163000	-3.087392000	0.829102000
H	5.839069000	-2.718753000	0.800942000
H	6.770722000	-0.521011000	0.040424000
H	5.208370000	1.252366000	-0.683746000
H	3.218156000	2.084861000	-1.212839000

H	1.290198000	3.362122000	-1.412856000
H	0.553358000	2.218510000	-2.548477000
O	-0.142717000	-0.853609000	-2.245745000
H	-0.883060000	-0.402079000	-2.671151000
H	0.636533000	-0.597756000	-2.754778000
S	0.388236000	0.598523000	2.099623000
C	-0.768096000	1.744504000	2.499276000
N	-1.563760000	2.552132000	2.802710000

Sum of electronic and zero-point Energies= -1538.023551
Sum of electronic and thermal Energies= -1537.999779
Sum of electronic and thermal Enthalpies= -1537.998835
Sum of electronic and thermal Free Energies= -1538.077676

[Ru(salen)(CO)DMSO]^0 S_0

Ru	0.046662000	-0.588441000	-0.181415000
C	0.287840000	-1.916244000	-1.409123000
O	0.448261000	-2.744748000	-2.208616000
O	-0.239417000	1.015636000	1.280519000
N	1.369476000	-1.364202000	1.132627000
O	1.509692000	0.640149000	-1.033488000
C	3.376698000	-0.395268000	0.158087000
C	2.781378000	0.465435000	-0.832028000
C	3.681233000	1.210961000	-1.645310000
C	5.050467000	1.118378000	-1.512307000
C	5.625300000	0.274803000	-0.546457000
C	4.786052000	-0.453496000	0.268918000
C	2.643797000	-1.179042000	1.120434000
C	0.703789000	-2.043068000	2.244103000
N	-1.274841000	-1.665807000	0.896021000
O	-1.470940000	0.385148000	-1.239756000
C	-3.301414000	-0.641225000	0.020561000
C	-2.732055000	0.239917000	-0.967683000
C	-3.652405000	1.037316000	-1.706982000
C	-5.015296000	0.976785000	-1.506455000
C	-5.564535000	0.111422000	-0.545313000
C	-4.705427000	-0.673145000	0.193562000
C	-2.554514000	-1.527641000	0.877249000
C	-0.608214000	-2.644914000	1.748595000
S	-0.240474000	2.442405000	0.673878000
C	-1.697226000	3.206186000	1.393933000
C	1.026134000	3.308746000	1.606383000
H	3.237774000	1.865098000	-2.390999000
H	5.689867000	1.708582000	-2.164447000
H	6.702758000	0.203888000	-0.439937000
H	5.208775000	-1.103120000	1.032962000
H	3.235939000	-1.642657000	1.915794000
H	1.345688000	-2.812082000	2.687584000
H	0.484421000	-1.291165000	3.012052000
H	-3.229342000	1.705884000	-2.451679000
H	-5.669265000	1.608813000	-2.102573000
H	-6.636906000	0.061992000	-0.387959000
H	-5.108169000	-1.350371000	0.944324000
H	-3.144416000	-2.139788000	1.566496000
H	-1.240372000	-2.942795000	2.592222000
H	-0.398269000	-3.535980000	1.143416000
H	-1.704428000	4.268325000	1.138595000
H	-1.690550000	3.064355000	2.476897000
H	-2.563881000	2.714329000	0.948106000
H	0.996665000	4.369285000	1.346115000

H	0.860525000	3.163428000	2.676174000
H	1.984787000	2.882052000	1.306275000

Sum of electronic and zero-point Energies= -1636.807177
Sum of electronic and thermal Energies= -1636.781391
Sum of electronic and thermal Enthalpies= -1636.780446
Sum of electronic and thermal Free Energies= -1636.863890

[Ru(salen)(CO)DMSO]^0 T$_1$

Ru	0.112791000	-0.663159000	-0.314191000
C	0.901703000	-1.841026000	-1.507414000
O	1.431899000	-2.543364000	-2.251969000
O	-0.660571000	0.747211000	1.126831000
N	1.404738000	-1.289204000	1.143468000
O	1.433444000	0.866377000	-0.795824000
C	3.356379000	-0.068546000	0.364477000
C	2.719151000	0.809257000	-0.576123000
C	3.553058000	1.712123000	-1.284158000
C	4.920646000	1.738371000	-1.101329000
C	5.542768000	0.874459000	-0.184943000
C	4.757799000	0.001014000	0.536929000
C	2.652505000	-0.981072000	1.228522000
C	0.738567000	-2.140437000	2.126143000
N	-1.078907000	-2.023558000	0.528319000
O	-1.368339000	-0.167327000	-1.578235000
C	-3.114226000	-0.757795000	0.034532000
C	-2.602928000	-0.011017000	-1.105901000
C	-3.447839000	0.881422000	-1.781516000
C	-4.765916000	1.098158000	-1.389298000
C	-5.273277000	0.387067000	-0.284790000
C	-4.476325000	-0.508786000	0.394728000
C	-2.378152000	-1.697566000	0.772265000
C	-0.356268000	-2.927549000	1.397128000
S	-0.852392000	2.205548000	0.622202000
C	-2.374299000	2.711868000	1.424882000
C	0.322033000	3.167965000	1.579921000
H	3.070235000	2.383230000	-1.988440000
H	5.520591000	2.440626000	-1.674435000
H	6.617666000	0.901316000	-0.040545000
H	5.216937000	-0.663837000	1.265267000
H	3.236518000	-1.434021000	2.035317000
H	1.456998000	-2.793988000	2.634905000
H	0.268598000	-1.486173000	2.870199000
H	-3.025544000	1.411916000	-2.631415000
H	-5.389340000	1.804767000	-1.928366000
H	-6.300851000	0.540309000	0.035038000
H	-4.881848000	-1.054182000	1.244395000
H	-2.881034000	-2.224538000	1.583941000
H	-1.023204000	-3.402870000	2.125637000
H	0.112844000	-3.722175000	0.799121000
H	-2.531151000	3.776464000	1.236609000
H	-2.305249000	2.510836000	2.496136000
H	-3.178978000	2.128746000	0.971606000
H	0.134795000	4.230005000	1.404936000
H	0.217518000	2.921785000	2.638891000
H	1.315426000	2.900694000	1.217114000

Sum of electronic and zero-point Energies= -1636.731142
Sum of electronic and thermal Energies= -1636.705095
Sum of electronic and thermal Enthalpies= -1636.704151
Sum of electronic and thermal Free Energies= -1636.787848

[Ru(salen)DMSO]^0 S_0

Ru	-0.072874000	0.566664000	-0.345279000
N	-1.414307000	1.741046000	0.553585000
O	-1.563249000	-0.734590000	-1.020277000
C	-3.432524000	0.581523000	-0.156790000
C	-2.834819000	-0.525484000	-0.863030000
C	-3.735320000	-1.467969000	-1.438704000
C	-5.105514000	-1.342910000	-1.343881000
C	-5.681188000	-0.259829000	-0.658274000
C	-4.842009000	0.670729000	-0.081449000
C	-2.701620000	1.620831000	0.525750000
C	-0.773875000	2.745490000	1.403347000
N	1.233090000	1.936399000	0.294169000
O	1.458093000	-0.583512000	-1.200350000
C	3.283539000	0.768611000	-0.296751000
C	2.720608000	-0.368609000	-0.982949000
C	3.648208000	-1.338263000	-1.462782000
C	5.012855000	-1.209514000	-1.300461000
C	5.553914000	-0.093747000	-0.641736000
C	4.686848000	0.865594000	-0.159064000
C	2.522382000	1.862854000	0.258126000
C	0.562009000	3.142698000	0.782703000
H	-3.289403000	-2.305235000	-1.969389000
H	-5.743479000	-2.093134000	-1.805470000
H	-6.759013000	-0.159058000	-0.580679000
H	-5.267530000	1.513969000	0.460079000
H	-3.302270000	2.354109000	1.073427000
H	-1.413434000	3.624370000	1.546106000
H	-0.594678000	2.290143000	2.385689000
H	3.229650000	-2.199977000	-1.976292000
H	5.671784000	-1.982311000	-1.689608000
H	6.626494000	0.013118000	-0.515413000
H	5.085645000	1.739476000	0.353373000
H	3.101918000	2.695762000	0.668855000
H	1.181772000	3.685362000	1.505878000
H	0.381944000	3.805364000	-0.073660000
O	0.181546000	-0.559761000	1.359039000
S	0.236654000	-2.100113000	1.071995000
C	1.775762000	-2.605544000	1.843807000
C	-0.924201000	-2.758046000	2.271993000
H	1.818051000	-3.697146000	1.858806000
H	1.829456000	-2.198530000	2.855790000
H	2.583977000	-2.212667000	1.223884000
H	-0.850901000	-3.848032000	2.271039000
H	-0.702346000	-2.351777000	3.260948000
H	-1.920573000	-2.454898000	1.946285000

Sum of electronic and zero-point Energies= -1523.548857
Sum of electronic and thermal Energies= -1523.525127
Sum of electronic and thermal Enthalpies= -1523.524183
Sum of electronic and thermal Free Energies= -1523.602933

[Ru(salen)DMSO]^0 T_1

Ru	0.060294000	0.599548000	-0.217125000
N	-1.243856000	1.690486000	0.835150000
O	-1.450365000	-0.439815000	-1.165195000
C	-3.284475000	0.838170000	-0.183305000
C	-2.715933000	-0.155490000	-1.058184000
C	-3.626003000	-0.894333000	-1.865375000

C	-4.987161000	-0.681935000	-1.826514000
C	-5.538017000	0.290053000	-0.971983000
C	-4.688924000	1.023068000	-0.173093000
C	-2.535018000	1.670580000	0.716223000
C	-0.595459000	2.497205000	1.869662000
N	1.398704000	1.797001000	0.666012000
O	1.524131000	-0.361718000	-1.294753000
C	3.408742000	0.733094000	-0.200107000
C	2.800254000	-0.223300000	-1.089398000
C	3.680786000	-1.077625000	-1.811053000
C	5.049691000	-1.014257000	-1.669234000
C	5.639965000	-0.079607000	-0.798405000
C	4.820888000	0.771935000	-0.091586000
C	2.689692000	1.705560000	0.572690000
C	0.780294000	2.923501000	1.370136000
H	-3.197040000	-1.644297000	-2.524237000
H	-5.638887000	-1.274065000	-2.464220000
H	-6.609781000	0.457219000	-0.940717000
H	-5.096922000	1.776768000	0.497490000
H	-3.120240000	2.338378000	1.355680000
H	-1.199749000	3.370925000	2.139567000
H	-0.483010000	1.869656000	2.762129000
H	3.222210000	-1.796256000	-2.484729000
H	5.678196000	-1.694277000	-2.239048000
H	6.718456000	-0.028195000	-0.689425000
H	5.258782000	1.507267000	0.580582000
H	3.296876000	2.437420000	1.114112000
H	1.406022000	3.269607000	2.200661000
H	0.671206000	3.751248000	0.658495000
O	0.113472000	-1.089832000	1.530973000
S	-0.073404000	-2.528393000	1.018059000
C	0.695792000	-3.563865000	2.273251000
C	-1.789288000	-2.955949000	1.349995000
H	0.507512000	-4.613774000	2.037062000
H	0.292618000	-3.307122000	3.255610000
H	1.768565000	-3.366701000	2.241051000
H	-1.939756000	-4.016102000	1.132481000
H	-2.027744000	-2.734791000	2.393167000
H	-2.402563000	-2.351042000	0.680181000

Sum of electronic and zero-point Energies= -1523.552308
Sum of electronic and thermal Energies= -1523.528018
Sum of electronic and thermal Enthalpies= -1523.527074
Sum of electronic and thermal Free Energies= -1523.609198

[Ru(salen)(H_2O)DMSO]^0 S_0

Ru	0.077358000	-0.522200000	-0.177659000
N	1.419625000	-1.524024000	0.902969000
O	1.561470000	0.724961000	-0.987919000
C	3.433811000	-0.485311000	0.012564000
C	2.831049000	0.523245000	-0.827598000
C	3.733032000	1.369993000	-1.537644000
C	5.103432000	1.243473000	-1.448990000
C	5.681777000	0.255656000	-0.633727000
C	4.843355000	-0.578222000	0.077199000
C	2.707135000	-1.408681000	0.850783000
C	0.784586000	-2.387212000	1.899040000
N	-1.217169000	-1.790272000	0.654936000
O	-1.465176000	0.494343000	-1.187268000
C	-3.278733000	-0.765295000	-0.137377000

C	-2.725326000	0.275744000	-0.970633000
C	-3.666184000	1.138481000	-1.606508000
C	-5.030936000	0.996652000	-1.457096000
C	-5.561098000	-0.025346000	-0.653110000
C	-4.682460000	-0.878551000	-0.016245000
C	-2.507642000	-1.744634000	0.591485000
C	-0.541342000	-2.893597000	1.339683000
H	3.286201000	2.134075000	-2.168794000
H	5.739798000	1.917770000	-2.017811000
H	6.759847000	0.154108000	-0.560180000
H	5.270553000	-1.344043000	0.722758000
H	3.311083000	-2.045598000	1.505140000
H	1.432779000	-3.224293000	2.184508000
H	0.591573000	-1.783837000	2.795083000
H	-3.256081000	1.926876000	-2.232561000
H	-5.698511000	1.685118000	-1.970303000
H	-6.633555000	-0.142777000	-0.534961000
H	-5.072813000	-1.678158000	0.611366000
H	-3.080335000	-2.509930000	1.125131000
H	-1.163761000	-3.318990000	2.135459000
H	-0.345642000	-3.686616000	0.605722000
O	0.388387000	-1.896309000	-1.847448000
H	1.118784000	-2.493811000	-1.640613000
H	-0.384967000	-2.463730000	-1.956512000
O	-0.208691000	0.876540000	1.381201000
S	-0.315233000	2.345550000	0.875922000
C	-1.904106000	2.905189000	1.500198000
C	0.756740000	3.237107000	2.008654000
H	-1.984011000	3.983731000	1.344757000
H	-1.989594000	2.656648000	2.560430000
H	-2.671896000	2.390175000	0.919484000
H	0.636632000	4.309911000	1.841096000
H	0.505285000	2.972765000	3.038128000
H	1.780937000	2.940237000	1.776985000

Sum of electronic and zero-point Energies= -1599.896811
Sum of electronic and thermal Energies= -1599.870344
Sum of electronic and thermal Enthalpies= -1599.869400
Sum of electronic and thermal Free Energies= -1599.953963

[Ru(salen)(CO)Phosphabenzene]^0 S_0

Ru	-0.089838000	-0.701031000	0.029294000
C	-0.403136000	-2.512870000	-0.276640000
O	-0.599977000	-3.629182000	-0.496603000
N	-1.376267000	-0.639111000	1.595704000
O	-1.602286000	-0.150720000	-1.324035000
C	-3.419314000	-0.392543000	0.298438000
C	-2.863661000	-0.199973000	-1.017587000
C	-3.796957000	-0.027438000	-2.078970000
C	-5.160535000	-0.050921000	-1.878295000
C	-5.697095000	-0.243252000	-0.593628000
C	-4.825327000	-0.401269000	0.461645000
C	-2.656188000	-0.526578000	1.514306000
C	-0.674598000	-0.631945000	2.877081000
C	3.264004000	-0.872325000	0.112039000
C	4.675244000	-0.878172000	0.230888000
C	5.513882000	-0.708520000	-0.848401000
C	4.936938000	-0.529107000	-2.118085000
C	3.568627000	-0.520460000	-2.278746000
C	2.665807000	-0.685773000	-1.187984000

C	2.545005000	-1.094351000	1.340764000
C	0.617343000	-1.435481000	2.746601000
N	1.264593000	-1.089582000	1.483768000
O	1.399396000	-0.655397000	-1.461041000
C	-0.476898000	2.886109000	-0.779734000
C	1.281109000	2.672013000	1.271675000
C	-0.231962000	4.250698000	-0.680857000
C	1.361270000	4.057874000	1.177850000
C	0.643129000	4.812668000	0.250213000
P	0.277739000	1.724518000	0.241345000
H	-3.383235000	0.122773000	-3.072332000
H	-5.825751000	0.082131000	-2.728110000
H	-6.770298000	-0.259698000	-0.435165000
H	-5.218124000	-0.539141000	1.467107000
H	-3.229009000	-0.520252000	2.446776000
H	-1.302054000	-1.030134000	3.681724000
H	-0.426937000	0.409971000	3.119532000
H	5.099281000	-1.026180000	1.222204000
H	6.591433000	-0.716886000	-0.722244000
H	5.575368000	-0.395295000	-2.988159000
H	3.124744000	-0.382094000	-3.260754000
H	3.155643000	-1.291959000	2.227222000
H	1.276288000	-1.248538000	3.601418000
H	0.379447000	-2.506731000	2.723552000
H	-1.170938000	2.507147000	-1.524798000
H	1.873992000	2.154461000	2.021403000
H	-0.750235000	4.917994000	-1.365812000
H	2.020485000	4.582010000	1.866012000
H	0.771204000	5.890975000	0.252990000

Sum of electronic and zero-point Energies= -1618.427770
Sum of electronic and thermal Energies= -1618.402280
Sum of electronic and thermal Enthalpies= -1618.401336
Sum of electronic and thermal Free Energies= -1618.485432

[Ru(salen)(CO)Phosphabenzene]^0 T_1

Ru	-0.005302000	-0.681793000	0.178833000
C	-0.707555000	-2.283760000	1.214098000
O	-1.331412000	-2.685053000	2.100136000
N	-1.271313000	0.397220000	1.351456000
O	-1.486931000	-0.847346000	-1.228656000
C	-3.327919000	-0.254988000	0.236651000
C	-2.762109000	-0.827951000	-0.950775000
C	-3.661157000	-1.363249000	-1.907125000
C	-5.025381000	-1.363503000	-1.699368000
C	-5.578497000	-0.805782000	-0.534307000
C	-4.731890000	-0.251320000	0.401462000
C	-2.557705000	0.413958000	1.251104000
C	-0.569577000	1.207093000	2.342752000
C	3.336359000	-0.617575000	0.346262000
C	4.732317000	-0.388971000	0.333625000
C	5.542563000	-0.876446000	-0.668522000
C	4.962706000	-1.643007000	-1.694247000
C	3.608633000	-1.906115000	-1.703853000
C	2.743476000	-1.403233000	-0.698739000
C	2.608506000	-0.087898000	1.466852000
C	0.669795000	0.438497000	2.790428000
N	1.323519000	-0.083311000	1.594889000
O	1.481207000	-1.723584000	-0.759112000
C	-0.878322000	2.425230000	-1.376942000

C	1.609336000	2.694817000	-0.376710000
C	-1.037833000	3.752579000	-1.013923000
C	1.226705000	3.999984000	-0.104137000
C	-0.039077000	4.516256000	-0.398695000
P	0.589237000	1.498658000	-1.149051000
H	-3.232324000	-1.785227000	-2.811206000
H	-5.678169000	-1.797901000	-2.452045000
H	-6.652270000	-0.801815000	-0.379518000
H	-5.141166000	0.203132000	1.300906000
H	-3.119872000	1.000591000	1.982604000
H	-1.217308000	1.444312000	3.192875000
H	-0.262378000	2.142129000	1.858795000
H	5.163412000	0.194255000	1.144184000
H	6.609331000	-0.679151000	-0.661997000
H	5.587731000	-2.039718000	-2.490055000
H	3.160619000	-2.506506000	-2.490047000
H	3.204116000	0.342939000	2.276046000
H	1.344133000	1.080406000	3.366325000
H	0.372017000	-0.408938000	3.419354000
H	-1.713753000	1.904107000	-1.838283000
H	2.619098000	2.385448000	-0.116017000
H	-1.995055000	4.231031000	-1.214283000
H	1.951582000	4.662529000	0.365891000
H	-0.253606000	5.550216000	-0.145113000

Sum of electronic and zero-point Energies= -1618.371337
Sum of electronic and thermal Energies= -1618.345006
Sum of electronic and thermal Enthalpies= -1618.344061
Sum of electronic and thermal Free Energies= -1618.431414

[Ru(salen)Phosphabenzene]^0 S_0

Ru	-0.054306000	-0.648430000	0.066597000
N	-1.362410000	-0.758439000	1.586878000
O	-1.570033000	-0.602226000	-1.357141000
C	-3.401396000	-0.744783000	0.256862000
C	-2.835181000	-0.636269000	-1.064438000
C	-3.758022000	-0.582782000	-2.147533000
C	-5.122768000	-0.630184000	-1.961415000
C	-5.669989000	-0.736893000	-0.670797000
C	-4.808811000	-0.793466000	0.403111000
C	-2.649178000	-0.812901000	1.482288000
C	-0.702518000	-0.708928000	2.890597000
C	3.280387000	-0.967520000	0.120554000
C	4.692301000	-0.982221000	0.214184000
C	5.509692000	-0.895421000	-0.892056000
C	4.911355000	-0.806695000	-2.160902000
C	3.539322000	-0.799842000	-2.295532000
C	2.661550000	-0.872577000	-1.177361000
C	2.568680000	-1.132791000	1.361591000
C	0.655049000	-1.397130000	2.787812000
N	1.289787000	-1.034271000	1.515793000
O	1.383959000	-0.874834000	-1.416555000
C	-0.458082000	2.601811000	-1.030161000
C	0.878014000	2.499811000	1.342755000
C	-0.283476000	3.976340000	-0.913873000
C	0.917079000	3.885910000	1.220449000
C	0.368761000	4.597061000	0.153024000
P	0.130898000	1.501524000	0.155981000
H	-3.335026000	-0.501774000	-3.145111000
H	-5.780621000	-0.583911000	-2.825975000

H	-6.744374000	-0.773525000	-0.523382000
H	-5.211121000	-0.876301000	1.410923000
H	-3.234019000	-0.910608000	2.402106000
H	-1.313057000	-1.176865000	3.670684000
H	-0.563212000	0.346851000	3.155787000
H	5.134599000	-1.068039000	1.204869000
H	6.589588000	-0.904179000	-0.786248000
H	5.534669000	-0.743916000	-3.049665000
H	3.076695000	-0.734362000	-3.276559000
H	3.174984000	-1.383177000	2.237774000
H	1.289850000	-1.134554000	3.641355000
H	0.509614000	-2.484458000	2.795552000
H	-0.981099000	2.184442000	-1.885317000
H	1.337880000	2.022595000	2.203321000
H	-0.683284000	4.605396000	-1.706758000
H	1.410679000	4.447491000	2.011041000
H	0.453598000	5.679226000	0.151553000

Sum of electronic and zero-point Energies= -1505.209181
Sum of electronic and thermal Energies= -1505.186373
Sum of electronic and thermal Enthalpies= -1505.185429
Sum of electronic and thermal Free Energies= -1505.262087

$[Ru(salen)Phosphabenzene]^0$ T_1

Ru	0.121213000	-0.564105000	0.296570000
N	-1.201549000	0.206933000	1.583980000
O	-1.357199000	-1.402247000	-0.851204000
C	-3.196001000	-0.823569000	0.639753000
C	-2.610001000	-1.461016000	-0.510780000
C	-3.484991000	-2.205408000	-1.350235000
C	-4.831369000	-2.322123000	-1.085887000
C	-5.402269000	-1.695841000	0.038306000
C	-4.587543000	-0.964026000	0.871226000
C	-2.479117000	-0.022881000	1.587780000
C	-0.595406000	1.126849000	2.544786000
C	3.462590000	-0.447317000	0.428484000
C	4.862405000	-0.240430000	0.420998000
C	5.684633000	-0.814816000	-0.523608000
C	5.113258000	-1.655240000	-1.495200000
C	3.756005000	-1.898411000	-1.507578000
C	2.874941000	-1.302174000	-0.566902000
C	2.733580000	0.146051000	1.515479000
C	0.810033000	0.631851000	2.866221000
N	1.444080000	0.169300000	1.628324000
O	1.607694000	-1.595441000	-0.651631000
H	-3.042078000	-2.684416000	-2.218820000
H	-5.458841000	-2.903571000	-1.756697000
H	-6.464091000	-1.786747000	0.242297000
H	-5.010677000	-0.469841000	1.743270000
H	-3.076377000	0.438722000	2.379312000
H	-1.196844000	1.210368000	3.456454000
H	-0.538825000	2.115353000	2.073051000
H	5.288115000	0.397016000	1.193028000
H	6.753865000	-0.630373000	-0.511677000
H	5.746533000	-2.123300000	-2.244568000
H	3.312146000	-2.552286000	-2.252864000
H	3.329737000	0.584667000	2.320976000
H	1.402664000	1.417364000	3.347684000
H	0.748815000	-0.220395000	3.553774000
C	-1.164328000	1.828440000	-1.962091000

C	0.598974000	3.019363000	-0.300484000
C	-1.806255000	3.058086000	-1.963903000
C	-0.200068000	4.141829000	-0.457782000
C	-1.353623000	4.169986000	-1.248304000
P	0.315099000	1.490154000	-1.099196000
H	-1.591440000	1.009032000	-2.534987000
H	1.479146000	3.088816000	0.334946000
H	-2.716144000	3.163933000	-2.551839000
H	0.082013000	5.053094000	0.066575000
H	-1.921944000	5.093530000	-1.306171000

Sum of electronic and zero-point Energies= -1505.184088
Sum of electronic and thermal Energies= -1505.160584
Sum of electronic and thermal Enthalpies= -1505.159640
Sum of electronic and thermal Free Energies= -1505.239936

[Ru(salen)(H_2O)Phosphabenzene]^0 S_0

Ru	-0.074607000	-0.558701000	0.095942000
N	-1.375006000	-0.542450000	1.624996000
O	-1.600992000	-0.422386000	-1.328940000
C	-3.418866000	-0.563167000	0.302407000
C	-2.859888000	-0.475471000	-1.025204000
C	-3.794072000	-0.454593000	-2.101860000
C	-5.156870000	-0.515864000	-1.906062000
C	-5.694943000	-0.602757000	-0.610254000
C	-4.824350000	-0.623646000	0.458027000
C	-2.660896000	-0.581175000	1.528410000
C	-0.702843000	-0.434558000	2.916955000
C	3.264359000	-0.892076000	0.161465000
C	4.674074000	-0.869764000	0.266063000
C	5.499791000	-0.811848000	-0.837563000
C	4.910816000	-0.793489000	-2.111608000
C	3.538304000	-0.826862000	-2.254918000
C	2.655775000	-0.873264000	-1.142594000
C	2.547687000	-1.004054000	1.410434000
C	0.631820000	-1.171974000	2.847654000
N	1.270311000	-0.899046000	1.556176000
O	1.374496000	-0.922075000	-1.390235000
C	-0.459901000	2.722408000	-1.062395000
C	1.032393000	2.649681000	1.204053000
C	-0.238988000	4.093799000	-1.007029000
C	1.106927000	4.029581000	1.039194000
C	0.505661000	4.725915000	-0.009435000
P	0.175013000	1.630098000	0.110096000
H	-3.379322000	-0.388403000	-3.104184000
H	-5.820313000	-0.496692000	-2.767595000
H	-6.767726000	-0.650159000	-0.454239000
H	-5.219060000	-0.687307000	1.470454000
H	-3.243013000	-0.620710000	2.454489000
H	-1.320641000	-0.832030000	3.729978000
H	-0.523100000	0.630081000	3.114405000
H	5.110237000	-0.899293000	1.262732000
H	6.578552000	-0.788708000	-0.722626000
H	5.538731000	-0.753294000	-2.998399000
H	3.082294000	-0.816467000	-3.241175000
H	3.154286000	-1.202849000	2.299497000
H	1.279536000	-0.885526000	3.683550000
H	0.450885000	-2.252037000	2.918129000
H	-1.053925000	2.296652000	-1.865883000
H	1.536881000	2.185036000	2.047007000

H	-0.675266000	4.711291000	-1.789827000
H	1.671305000	4.598731000	1.775258000
H	0.622136000	5.804486000	-0.051798000
O	-0.225387000	-2.790294000	-0.254784000
H	0.451730000	-2.711620000	-0.950609000
H	0.195317000	-3.302247000	0.446961000

Sum of electronic and zero-point Energies= -1581.550321
Sum of electronic and thermal Energies= -1581.524830
Sum of electronic and thermal Enthalpies= -1581.523886
Sum of electronic and thermal Free Energies= -1581.606512

[Ru(salen)(CO)Phosphole]$^{-1}$ S_0

Ru	0.043344000	-0.495771000	0.199005000
C	-0.129111000	-2.172060000	0.979131000
O	-0.252817000	-3.218553000	1.465537000
N	-1.276847000	0.335615000	1.484489000
O	-1.485640000	-0.955500000	-1.178465000
C	-3.307048000	-0.371118000	0.345270000
C	-2.739831000	-0.920266000	-0.863706000
C	-3.667696000	-1.459891000	-1.803559000
C	-5.028174000	-1.472976000	-1.583506000
C	-5.572569000	-0.939223000	-0.402475000
C	-4.709730000	-0.398083000	0.526750000
C	-2.559199000	0.271726000	1.398660000
C	-0.611523000	1.122392000	2.519454000
C	3.402377000	-0.341899000	0.285872000
C	4.806419000	-0.181487000	0.360024000
C	5.659750000	-0.592823000	-0.641271000
C	5.102732000	-1.201807000	-1.779618000
C	3.741054000	-1.380017000	-1.891314000
C	2.821477000	-0.965437000	-0.880891000
C	2.669446000	0.122312000	1.438175000
C	0.744758000	0.494103000	2.832505000
N	1.390322000	0.107829000	1.580978000
O	1.566670000	-1.184516000	-1.095186000
C	-1.040852000	2.625355000	-1.683805000
C	0.738353000	3.228173000	0.007655000
C	-1.076628000	3.951065000	-1.295088000
C	-0.074558000	4.291184000	-0.340061000
P	0.320784000	1.783948000	-0.928029000
H	-3.248908000	-1.871539000	-2.718024000
H	-5.685295000	-1.902080000	-2.336532000
H	-6.643727000	-0.946787000	-0.229102000
H	-5.109673000	0.031674000	1.443533000
H	-3.150322000	0.755690000	2.182912000
H	-1.224409000	1.197029000	3.424873000
H	-0.448463000	2.127617000	2.111280000
H	5.215231000	0.287091000	1.253605000
H	6.732068000	-0.453231000	-0.551359000
H	5.751484000	-1.538478000	-2.585135000
H	3.313389000	-1.851043000	-2.772416000
H	3.271508000	0.511418000	2.265680000
H	1.367212000	1.187668000	3.409225000
H	0.598756000	-0.414359000	3.431623000
H	-1.716926000	2.178179000	-2.405660000
H	1.565091000	3.289766000	0.708801000
H	-1.785765000	4.677262000	-1.687919000
H	0.043166000	5.297942000	0.056258000
H	-0.954666000	0.090665000	-3.138084000

H	3.971937000	3.825086000	-1.272754000
H	5.244052000	3.939967000	0.669173000
H	4.334975000	3.278817000	2.866294000
H	2.037754000	2.353086000	3.055088000
H	1.853797000	2.993004000	-2.450243000
H	0.152771000	2.136620000	-3.811404000
H	-1.116400000	3.136080000	-3.074363000
H	-0.228564000	-0.715599000	1.867626000
H	0.160795000	-0.425846000	-2.374517000
H	0.987025000	-3.037543000	1.041555000
H	1.235450000	-2.852988000	-1.662315000

Sum of electronic and zero-point Energies= -1579.897001
Sum of electronic and thermal Energies= -1579.872346
Sum of electronic and thermal Enthalpies= -1579.871402
Sum of electronic and thermal Free Energies= -1579.952907

$[Ru(salen)(CO)Phosphole]^{-1}$ T_1

Ru	0.043142000	-0.604753000	0.278516000
C	-0.594530000	-2.195163000	1.115468000
O	-1.072567000	-3.126338000	1.596518000
N	-1.250527000	0.451258000	1.450909000
O	-1.447959000	-0.849339000	-1.182308000
C	-3.284784000	-0.077058000	0.223204000
C	-2.713722000	-0.692287000	-0.947228000
C	-3.628583000	-1.141036000	-1.941428000
C	-4.994010000	-1.020344000	-1.795223000
C	-5.546853000	-0.427222000	-0.646920000
C	-4.690725000	0.038029000	0.327897000
C	-2.527008000	0.513550000	1.297473000
C	-0.557524000	1.182701000	2.508100000
C	3.329323000	-0.263525000	0.294980000
C	4.702516000	0.108811000	0.126015000
C	5.475034000	-0.349579000	-0.919637000
C	4.930264000	-1.223769000	-1.877559000
C	3.597148000	-1.612609000	-1.752642000
C	2.772222000	-1.156603000	-0.714235000
C	2.636564000	0.194124000	1.426562000
C	0.687646000	0.381534000	2.903299000
N	1.335508000	-0.088351000	1.701798000
O	1.526686000	-1.606326000	-0.676326000
C	-0.828769000	2.382633000	-1.559446000
C	1.150890000	2.937889000	-0.075381000
C	-0.767199000	3.707497000	-1.197075000
C	0.353094000	4.021835000	-0.359554000
P	0.587287000	1.498206000	-0.952667000
H	-3.201339000	-1.596113000	-2.830656000
H	-5.648289000	-1.388082000	-2.581992000
H	-6.621605000	-0.329426000	-0.534632000
H	-5.096664000	0.515927000	1.217361000
H	-3.101112000	1.081516000	2.036196000
H	-1.221184000	1.364298000	3.361981000
H	-0.237541000	2.142574000	2.084241000
H	5.138442000	0.777946000	0.865557000
H	6.512676000	-0.033736000	-0.998163000
H	5.533781000	-1.589999000	-2.702754000
H	3.145879000	-2.286780000	-2.477166000
H	3.179593000	0.798316000	2.155266000
H	1.358820000	1.007566000	3.504289000
H	0.380437000	-0.474216000	3.525935000

H	-1.599614000	1.947331000	-2.185925000
H	2.051268000	2.968105000	0.528796000
H	-1.483963000	4.459392000	-1.521277000
H	0.552757000	5.030661000	-0.003773000

Sum of electronic and zero-point Energies= -1579.825870
Sum of electronic and thermal Energies= -1579.800776
Sum of electronic and thermal Enthalpies= -1579.799832
Sum of electronic and thermal Free Energies= -1579.882675

$[Ru(salen)Phosphole]^{-1}$ S_0

Ru	-0.067711000	-0.386085000	0.296790000
N	-1.384394000	0.723532000	1.294664000
O	-1.596642000	-1.477640000	-0.638236000
C	-3.430905000	-0.308921000	0.473712000
C	-2.860380000	-1.292395000	-0.416445000
C	-3.785248000	-2.138552000	-1.096097000
C	-5.151179000	-2.032735000	-0.936152000
C	-5.699080000	-1.067925000	-0.073947000
C	-4.836434000	-0.234906000	0.608921000
C	-2.674357000	0.626782000	1.268952000
C	-0.732304000	1.797987000	2.040826000
C	3.280056000	-0.527709000	0.614317000
C	4.688165000	-0.461233000	0.719762000
C	5.532215000	-1.262166000	-0.022853000
C	4.961483000	-2.190793000	-0.909471000
C	3.591571000	-2.292299000	-1.036511000
C	2.685893000	-1.474273000	-0.299126000
C	2.539395000	0.337060000	1.500082000
C	0.617197000	1.300951000	2.547581000
N	1.254838000	0.489958000	1.504310000
O	1.416433000	-1.652904000	-0.486677000
C	-0.936640000	2.577480000	-1.454645000
C	1.586793000	2.257241000	-1.414253000
C	-0.226181000	3.721359000	-1.743396000
C	1.194700000	3.540477000	-1.721588000
P	0.167834000	1.193152000	-1.317742000
H	-3.361208000	-2.884358000	-1.763791000
H	-5.807278000	-2.704178000	-1.485560000
H	-6.773381000	-0.980084000	0.053714000
H	-5.240491000	0.516939000	1.285139000
H	-3.256294000	1.307158000	1.898922000
H	-1.352763000	2.150910000	2.873549000
H	-0.580532000	2.628617000	1.339905000
H	5.109965000	0.255022000	1.423250000
H	6.609543000	-1.180936000	0.081679000
H	5.602765000	-2.838542000	-1.503105000
H	3.149913000	-3.012144000	-1.721154000
H	3.128536000	0.885769000	2.242198000
H	1.253904000	2.142434000	2.845217000
H	0.464278000	0.667208000	3.430532000
H	-2.019301000	2.506115000	-1.443659000
H	2.615067000	1.915338000	-1.362746000
H	-0.688462000	4.670047000	-2.009299000
H	1.887532000	4.342458000	-1.968953000

Sum of electronic and zero-point Energies= -1466.671176
Sum of electronic and thermal Energies= -1466.648789
Sum of electronic and thermal Enthalpies= -1466.647844
Sum of electronic and thermal Free Energies= -1466.724084

[Ru(salen)Phosphole]$^{-1}$ T_1

Ru	-0.126564000	-0.351492000	-0.263404000
N	1.212451000	0.500655000	-1.475596000
O	1.332911000	-1.458553000	0.695392000
C	3.191411000	-0.670407000	-0.673620000
C	2.585749000	-1.476459000	0.357203000
C	3.447392000	-2.367353000	1.059690000
C	4.796365000	-2.454431000	0.793910000
C	5.384652000	-1.657810000	-0.205912000
C	4.581858000	-0.792406000	-0.915410000
C	2.485961000	0.256106000	-1.514018000
C	0.616809000	1.520699000	-2.336294000
C	-3.478093000	-0.324671000	-0.492580000
C	-4.886107000	-0.195652000	-0.559515000
C	-5.732975000	-0.866168000	0.296291000
C	-5.174257000	-1.726879000	1.258115000
C	-3.808524000	-1.891999000	1.345536000
C	-2.900351000	-1.199144000	0.495958000
C	-2.726859000	0.384645000	-1.492048000
C	-0.789485000	1.073927000	-2.717030000
N	-1.434445000	0.470690000	-1.546168000
O	-1.630707000	-1.418587000	0.661801000
C	1.252650000	1.858886000	2.330125000
C	-0.046100000	3.284283000	0.707573000
C	1.836858000	3.089357000	2.094339000
C	1.100294000	3.897222000	1.180051000
P	-0.268387000	1.676876000	1.429965000
H	2.991075000	-2.979909000	1.832646000
H	5.410472000	-3.146379000	1.365311000
H	6.447428000	-1.725446000	-0.415142000
H	5.017436000	-0.171629000	-1.696201000
H	3.088801000	0.791629000	-2.254002000
H	1.222874000	1.699449000	-3.232354000
H	0.562654000	2.446539000	-1.751200000
H	-5.300041000	0.462895000	-1.320850000
H	-6.808354000	-0.738655000	0.224222000
H	-5.823513000	-2.270586000	1.940203000
H	-3.374088000	-2.558896000	2.085449000
H	-3.309845000	0.867888000	-2.282469000
H	-1.374872000	1.916101000	-3.104116000
H	-0.731648000	0.311896000	-3.504472000
H	1.662800000	1.103465000	2.993852000
H	-0.745436000	3.747264000	0.017102000
H	2.760893000	3.421976000	2.564037000
H	1.410819000	4.902145000	0.898902000

Sum of electronic and zero-point Energies= -1466.651168
Sum of electronic and thermal Energies= -1466.628204
Sum of electronic and thermal Enthalpies= -1466.627260
Sum of electronic and thermal Free Energies= -1466.707427

[Ru(salen)(H_2O)Phosphole]$^{-1}$ S_0

Ru	0.016417000	-0.371093000	0.173252000
N	-1.294556000	0.373059000	1.473274000
O	-1.528119000	-1.044792000	-1.096769000
C	-3.338750000	-0.440421000	0.427238000
C	-2.778079000	-1.026544000	-0.769573000
C	-3.709581000	-1.639627000	-1.661275000
C	-5.065885000	-1.681064000	-1.415131000
C	-5.601834000	-1.108111000	-0.249151000

C	-4.734992000	-0.503870000	0.638915000
C	-2.580913000	0.236794000	1.452385000
C	-0.639528000	1.176504000	2.502725000
C	3.380370000	-0.501891000	0.382864000
C	4.788226000	-0.428864000	0.490291000
C	5.640065000	-0.907061000	-0.484430000
C	5.076090000	-1.501917000	-1.626428000
C	3.708098000	-1.600494000	-1.767254000
C	2.790348000	-1.112233000	-0.787637000
C	2.639786000	0.011909000	1.509594000
C	0.714555000	0.552459000	2.826373000
N	1.354134000	0.126573000	1.578736000
O	1.527808000	-1.268926000	-1.009486000
C	-0.995811000	2.549513000	-1.774799000
C	0.881746000	3.145326000	-0.178566000
C	-0.820812000	3.913971000	-1.605609000
C	0.231446000	4.248564000	-0.709150000
P	0.257293000	1.664185000	-0.904549000
H	-3.296524000	-2.083029000	-2.563975000
H	-5.724434000	-2.163725000	-2.134057000
H	-6.668855000	-1.137236000	-0.052336000
H	-5.129278000	-0.050302000	1.547192000
H	-3.159569000	0.669287000	2.275125000
H	-1.254171000	1.263278000	3.406926000
H	-0.478053000	2.177383000	2.083034000
H	5.203601000	0.027172000	1.387851000
H	6.716578000	-0.830123000	-0.369181000
H	5.722912000	-1.890787000	-2.409979000
H	3.274012000	-2.062859000	-2.650364000
H	3.233522000	0.321740000	2.376151000
H	1.345096000	1.257043000	3.381568000
H	0.565022000	-0.332918000	3.458707000
H	-1.733145000	2.099413000	-2.431721000
H	1.719007000	3.192326000	0.510353000
H	-1.403270000	4.665675000	-2.134937000
H	0.516306000	5.276905000	-0.495084000
O	-0.213476000	-2.425392000	1.187900000
H	-0.863161000	-2.310993000	1.892353000
H	0.618639000	-2.605133000	1.642034000

Sum of electronic and zero-point Energies= -1543.005544
Sum of electronic and thermal Energies= -1542.980268
Sum of electronic and thermal Enthalpies= -1542.979324
Sum of electronic and thermal Free Energies= -1543.061508

$[Ru(salen)(CO)TPH]^0$ S_0

Ru	0.058511000	-0.751772000	0.088720000
C	0.322755000	-2.615022000	0.061483000
O	0.490713000	-3.756590000	0.003609000
P	-0.240612000	1.666472000	-0.133591000
N	1.383847000	-0.478508000	1.594962000
O	1.567494000	-0.465675000	-1.363456000
C	3.407571000	-0.565422000	0.247576000
C	2.830697000	-0.541638000	-1.074180000
C	3.747287000	-0.588183000	-2.163434000
C	5.111865000	-0.662887000	-1.982542000
C	5.667810000	-0.689784000	-0.692018000
C	4.814102000	-0.632858000	0.388370000
C	2.666731000	-0.459745000	1.481325000
C	0.709885000	-0.234182000	2.868406000

O	-1.477908000	-0.883397000	-1.353790000
C	-3.302198000	-0.848781000	0.279609000
C	-2.737922000	-0.858806000	-1.048146000
C	-3.667762000	-0.835145000	-2.128145000
C	-5.032023000	-0.801528000	-1.933558000
C	-5.575537000	-0.791754000	-0.637576000
C	-4.709056000	-0.817439000	0.433662000
C	-2.549366000	-0.892230000	1.509598000
C	-0.591502000	-1.031738000	2.900531000
C	-0.723327000	2.077055000	-1.862836000
O	-0.711348000	3.474072000	-2.049820000
N	-1.266407000	-0.881302000	1.613915000
C	-1.454909000	2.612599000	0.915042000
O	-0.929376000	2.839139000	2.201650000
C	1.308474000	2.657987000	0.137260000
O	0.989445000	3.953222000	0.621169000
H	3.318921000	-0.565496000	-3.161763000
H	5.762704000	-0.700661000	-2.852883000
H	6.741897000	-0.746284000	-0.549357000
H	5.222846000	-0.638638000	1.397031000
H	3.260056000	-0.338252000	2.393024000
H	1.349723000	-0.493205000	3.719130000
H	0.464095000	0.835149000	2.921405000
H	-3.249115000	-0.844743000	-3.130788000
H	-5.692205000	-0.782979000	-2.797455000
H	-6.649231000	-0.767057000	-0.483622000
H	-5.107530000	-0.816120000	1.446414000
H	-3.136800000	-0.943537000	2.431615000
H	-1.226865000	-0.700629000	3.729211000
H	-0.365756000	-2.096580000	3.042726000
H	-0.003263000	1.552789000	-2.507618000
H	-1.710303000	1.629401000	-2.040487000
H	-0.997821000	3.648990000	-2.952978000
H	-1.706198000	3.544536000	0.393286000
H	-2.363913000	2.012448000	1.009578000
H	-0.185462000	3.447866000	2.052325000
H	1.844594000	2.698305000	-0.819533000
H	1.934818000	2.108894000	0.849618000
H	1.813806000	4.386186000	0.871704000

Sum of electronic and zero-point Energies= -1770.097829
Sum of electronic and thermal Energies= -1770.067822
Sum of electronic and thermal Enthalpies= -1770.066878
Sum of electronic and thermal Free Energies= -1770.159591

[Ru(salen)(CO)TPH]^0 T_1

Ru	0.136959000	-0.719331000	0.091464000
C	0.850058000	-2.527148000	0.139065000
O	1.335032000	-3.563961000	0.161106000
P	-0.520775000	1.599648000	-0.137372000
N	1.427197000	-0.313449000	1.598492000
O	1.556917000	-0.136721000	-1.257423000
C	3.461326000	-0.349241000	0.248684000
C	2.856391000	-0.268665000	-1.059433000
C	3.693909000	-0.277876000	-2.189661000
C	5.074496000	-0.372249000	-2.080955000
C	5.672924000	-0.447676000	-0.811689000
C	4.880583000	-0.429225000	0.316159000
C	2.742888000	-0.278151000	1.474247000
C	0.753475000	-0.111633000	2.870110000

O	-1.316977000	-1.267758000	-1.217382000
C	-3.196314000	-1.014462000	0.314237000
C	-2.611278000	-1.148679000	-0.998330000
C	-3.471710000	-1.219109000	-2.111909000
C	-4.851246000	-1.141239000	-1.982330000
C	-5.428423000	-1.013450000	-0.707748000
C	-4.615476000	-0.961065000	0.404392000
C	-2.455298000	-1.008198000	1.528629000
C	-0.455801000	-1.041761000	2.906487000
C	-1.107633000	1.913724000	-1.853601000
O	-1.272507000	3.298223000	-2.050814000
N	-1.142052000	-0.913554000	1.632129000
C	-1.773222000	2.429533000	0.961820000
O	-1.206972000	2.779629000	2.198950000
C	0.951527000	2.709645000	0.060519000
O	0.534397000	3.993607000	0.494668000
H	3.214489000	-0.209126000	-3.162515000
H	5.687194000	-0.383262000	-2.977876000
H	6.752712000	-0.516049000	-0.717254000
H	5.340196000	-0.479656000	1.300934000
H	3.327906000	-0.185621000	2.391608000
H	1.427640000	-0.300883000	3.712835000
H	0.396341000	0.928339000	2.927285000
H	-3.009038000	-1.333449000	-3.088529000
H	-5.479568000	-1.184677000	-2.867325000
H	-6.507361000	-0.960000000	-0.596389000
H	-5.058287000	-0.872691000	1.394113000
H	-3.022632000	-1.080962000	2.458533000
H	-1.120196000	-0.793814000	3.741630000
H	-0.115955000	-2.082132000	3.031898000
H	-0.348962000	1.473091000	-2.516833000
H	-2.040280000	1.348453000	-1.990860000
H	-1.639058000	3.426477000	-2.932762000
H	-2.162934000	3.296481000	0.412860000
H	-2.592749000	1.727959000	1.135445000
H	-0.542179000	3.456145000	1.981825000
H	1.466419000	2.746719000	-0.907163000
H	1.625572000	2.234037000	0.783353000
H	1.323971000	4.506859000	0.701989000

Sum of electronic and zero-point Energies= -1770.011708
Sum of electronic and thermal Energies= -1769.981300
Sum of electronic and thermal Enthalpies= -1769.980356
Sum of electronic and thermal Free Energies= -1770.073763

[Ru(salen)TPH]^0 S_0

Ru	0.058511000	-0.751772000	0.088720000
C	0.322755000	-2.615022000	0.061483000
O	0.490713000	-3.756590000	0.003609000
P	-0.240612000	1.666472000	-0.133591000
N	1.383847000	-0.478508000	1.594962000
O	1.567494000	-0.465675000	-1.363456000
C	3.407571000	-0.565422000	0.247576000
C	2.830697000	-0.541638000	-1.074180000
C	3.747287000	-0.588183000	-2.163434000
C	5.111865000	-0.662887000	-1.982542000
C	5.667810000	-0.689784000	-0.692018000
C	4.814102000	-0.632858000	0.388370000
C	2.666731000	-0.459745000	1.481325000
C	0.709885000	-0.234182000	2.868406000

O	-1.477908000	-0.883397000	-1.353790000
C	-3.302198000	-0.848781000	0.279609000
C	-2.737922000	-0.858806000	-1.048146000
C	-3.667762000	-0.835145000	-2.128145000
C	-5.032023000	-0.801528000	-1.933558000
C	-5.575537000	-0.791754000	-0.637576000
C	-4.709056000	-0.817439000	0.433662000
C	-2.549366000	-0.892230000	1.509598000
C	-0.591502000	-1.031738000	2.900531000
C	-0.723327000	2.077055000	-1.862836000
O	-0.711348000	3.474072000	-2.049820000
N	-1.266407000	-0.881302000	1.613915000
C	-1.454909000	2.612599000	0.915042000
O	-0.929376000	2.839139000	2.201650000
C	1.308474000	2.657987000	0.137260000
O	0.989445000	3.953222000	0.621169000
H	3.318921000	-0.565496000	-3.161763000
H	5.762704000	-0.700661000	-2.852883000
H	6.741897000	-0.746284000	-0.549357000
H	5.222846000	-0.638638000	1.397031000
H	3.260056000	-0.338252000	2.393024000
H	1.349723000	-0.493205000	3.719130000
H	0.464095000	0.835149000	2.921405000
H	-3.249115000	-0.844743000	-3.130788000
H	-5.692205000	-0.782979000	-2.797455000
H	-6.649231000	-0.767057000	-0.483622000
H	-5.107530000	-0.816120000	1.446414000
H	-3.136800000	-0.943537000	2.431615000
H	-1.226865000	-0.700629000	3.729211000
H	-0.365756000	-2.096580000	3.042726000
H	-0.003263000	1.552789000	-2.507618000
H	-1.710303000	1.629401000	-2.040487000
H	-0.997821000	3.648990000	-2.952978000
H	-1.706198000	3.544536000	0.393286000
H	-2.363913000	2.012448000	1.009578000
H	-0.185462000	3.447866000	2.052325000
H	1.844594000	2.698305000	-0.819533000
H	1.934818000	2.108894000	0.849618000
H	1.813806000	4.386186000	0.871704000

Sum of electronic and zero-point Energies= -1770.097829
Sum of electronic and thermal Energies= -1770.067822
Sum of electronic and thermal Enthalpies= -1770.066878
Sum of electronic and thermal Free Energies= -1770.159591

$[Ru(salen)TPH]^0$ T_1

Ru	0.132689000	-0.772106000	0.184866000
P	-0.410064000	1.685398000	-0.386621000
N	1.479564000	-0.405463000	1.621356000
O	1.614927000	-0.942271000	-1.241623000
C	3.491965000	-0.787065000	0.307007000
C	2.887112000	-1.010473000	-0.980678000
C	3.765578000	-1.314391000	-2.058011000
C	5.131889000	-1.387310000	-1.895448000
C	5.719757000	-1.158453000	-0.637843000
C	4.900986000	-0.863594000	0.429388000
C	2.769971000	-0.470842000	1.507344000
C	0.852684000	0.019459000	2.873993000
O	-1.345786000	-1.487048000	-1.065297000
C	-3.190931000	-1.156520000	0.490301000

C	-2.618203000	-1.444467000	-0.798944000
C	-3.526660000	-1.729392000	-1.855680000
C	-4.893342000	-1.709572000	-1.675867000
C	-5.448814000	-1.423170000	-0.416034000
C	-4.598812000	-1.165919000	0.637220000
C	-2.427364000	-0.942207000	1.689255000
C	-0.454251000	-0.747670000	3.039728000
C	-1.416956000	1.986684000	-1.906544000
O	-1.590838000	3.363925000	-2.153681000
N	-1.141845000	-0.792727000	1.746293000
C	-1.255605000	2.763514000	0.885424000
O	-0.353240000	3.152737000	1.894643000
C	1.149243000	2.655506000	-0.669817000
O	0.978856000	4.024074000	-0.329911000
H	3.309359000	-1.485525000	-3.029105000
H	5.760091000	-1.621887000	-2.751227000
H	6.796217000	-1.212127000	-0.511614000
H	5.336731000	-0.682228000	1.409870000
H	3.373971000	-0.265009000	2.396069000
H	1.513652000	-0.148991000	3.731988000
H	0.637956000	1.092605000	2.793495000
H	-3.094848000	-1.955815000	-2.826582000
H	-5.546121000	-1.921219000	-2.519121000
H	-6.524628000	-1.413845000	-0.274309000
H	-5.008928000	-0.959297000	1.623699000
H	-2.990273000	-0.931959000	2.627634000
H	-1.083168000	-0.286795000	3.809909000
H	-0.238494000	-1.778648000	3.346380000
H	-0.900677000	1.474006000	-2.732654000
H	-2.372863000	1.463844000	-1.752206000
H	-2.219358000	3.453072000	-2.879077000
H	-1.705392000	3.621967000	0.371177000
H	-2.055772000	2.178966000	1.348464000
H	0.291483000	3.718813000	1.436175000
H	1.431923000	2.525399000	-1.723228000
H	1.932412000	2.189705000	-0.057334000
H	1.844498000	4.447945000	-0.368550000

Sum of electronic and zero-point Energies= -1656.843813
Sum of electronic and thermal Energies= -1656.815572
Sum of electronic and thermal Enthalpies= -1656.814628
Sum of electronic and thermal Free Energies= -1656.905145

[Ru(salen)(H_2O)TPH]^0 S_0

Ru	0.121485000	-0.588558000	0.107939000
P	-0.144682000	1.635360000	-0.041431000
N	1.454875000	-0.509051000	1.608299000
O	1.643660000	-0.533605000	-1.343761000
C	3.482405000	-0.641013000	0.264056000
C	2.904568000	-0.605161000	-1.058793000
C	3.824612000	-0.656102000	-2.147888000
C	5.188519000	-0.739488000	-1.968345000
C	5.743577000	-0.777246000	-0.677105000
C	4.888165000	-0.727010000	0.402748000
C	2.740383000	-0.589502000	1.499179000
C	0.817160000	-0.392514000	2.918926000
O	-1.361010000	-0.921565000	-1.340987000
C	-3.205466000	-1.076486000	0.253473000
C	-2.623328000	-1.013854000	-1.065862000
C	-3.534481000	-1.087575000	-2.160514000

C	-4.897176000	-1.201317000	-1.988346000
C	-5.457598000	-1.257885000	-0.699989000
C	-4.608761000	-1.201537000	0.384974000
C	-2.460015000	-1.084078000	1.486731000
C	-0.521212000	-1.125463000	2.889135000
C	1.437430000	2.485013000	-0.478782000
O	1.262865000	3.881213000	-0.566348000
N	-1.186572000	-0.892059000	1.602967000
C	-1.313230000	2.294203000	-1.344157000
O	-2.621394000	2.476417000	-0.861943000
C	-0.742054000	2.536444000	1.470902000
O	-1.328605000	3.783081000	1.129252000
H	3.397127000	-0.627308000	-3.146809000
H	5.839634000	-0.776111000	-2.838779000
H	6.817239000	-0.842296000	-0.533460000
H	5.296351000	-0.752132000	1.411704000
H	3.333903000	-0.614101000	2.418672000
H	1.454442000	-0.795166000	3.714696000
H	0.652603000	0.670693000	3.129225000
H	-3.102162000	-1.045558000	-3.156920000
H	-5.543150000	-1.248908000	-2.862113000
H	-6.530096000	-1.350763000	-0.562688000
H	-5.020029000	-1.256387000	1.391528000
H	-3.034327000	-1.292748000	2.395369000
H	-1.152266000	-0.816579000	3.730272000
H	-0.339986000	-2.203791000	2.987647000
H	2.177295000	2.198418000	0.283885000
H	1.761140000	2.035963000	-1.427828000
H	2.066062000	4.249612000	-0.949965000
H	-0.874428000	3.224710000	-1.730460000
H	-1.344276000	1.552123000	-2.145568000
H	-2.533943000	3.136883000	-0.153311000
H	0.118741000	2.668916000	2.140482000
H	-1.461396000	1.875620000	1.971183000
H	-1.721175000	4.153497000	1.928386000
O	0.426984000	-2.858690000	0.050995000
H	0.962964000	-3.147591000	0.799683000
H	-0.434474000	-3.274468000	0.178740000

Sum of electronic and zero-point Energies= -1733.213767
Sum of electronic and thermal Energies= -1733.183425
Sum of electronic and thermal Enthalpies= -1733.182481
Sum of electronic and thermal Free Energies= -1733.274342

$[Ru(salen)(CO)CN]^{-1}$ S_0

Ru	0.002181000	0.065068000	0.069413000
C	0.247422000	-0.024385000	1.948086000
O	0.405166000	-0.104420000	3.091953000
C	-0.266724000	0.108398000	-2.002975000
N	-0.420636000	0.134495000	-3.164361000
N	1.322603000	1.580552000	-0.148647000
O	1.534428000	-1.381269000	-0.184571000
C	3.357805000	0.247035000	-0.126707000
C	2.790097000	-1.081341000	-0.136350000
C	3.723234000	-2.161422000	-0.106437000
C	5.086796000	-1.967508000	-0.065065000
C	5.631123000	-0.671090000	-0.055801000
C	4.764061000	0.399938000	-0.092830000
C	2.603944000	1.474226000	-0.203768000
C	0.644363000	2.853080000	-0.374877000

N	-1.333026000	1.575744000	0.216393000
O	-1.535464000	-1.390524000	0.133186000
C	-3.359868000	0.237475000	0.057183000
C	-2.789071000	-1.089579000	0.057044000
C	-3.718220000	-2.170082000	-0.030602000
C	-5.080302000	-1.978110000	-0.112182000
C	-5.627544000	-0.683089000	-0.111774000
C	-4.764196000	0.388285000	-0.025435000
C	-2.613722000	1.467393000	0.161465000
C	-0.670293000	2.862026000	0.402202000
H	3.304866000	-3.164464000	-0.116469000
H	5.747012000	-2.831577000	-0.040650000
H	6.704890000	-0.517282000	-0.026209000
H	5.162912000	1.412904000	-0.097153000
H	3.188752000	2.391488000	-0.328837000
H	1.273412000	3.703585000	-0.088736000
H	0.423717000	2.927389000	-1.447195000
H	-3.297853000	-3.172331000	-0.030640000
H	-5.736755000	-2.842923000	-0.177824000
H	-6.700000000	-0.530353000	-0.174860000
H	-5.165314000	1.400358000	-0.018178000
H	-3.206916000	2.386670000	0.205659000
H	-1.305287000	3.694793000	0.079239000
H	-0.458458000	2.985437000	1.472400000

Sum of electronic and zero-point Energies= -1176.864726
Sum of electronic and thermal Energies= -1176.843192
Sum of electronic and thermal Enthalpies= -1176.842248
Sum of electronic and thermal Free Energies= -1176.915703

[Ru(salen)(CO)CN]$^{-1}$ T$_1$

Ru	-0.015982000	0.068486000	0.068309000
C	0.634875000	0.067157000	1.918295000
O	1.090322000	0.058896000	2.969537000
C	-0.580490000	0.032263000	-1.906270000
N	-0.888139000	0.000916000	-3.034683000
N	1.275844000	1.591624000	-0.239480000
O	1.446363000	-1.285127000	-0.412001000
C	3.323125000	0.259049000	-0.198614000
C	2.734668000	-1.060466000	-0.275609000
C	3.597262000	-2.175071000	-0.262393000
C	4.975943000	-2.045360000	-0.159785000
C	5.553949000	-0.766869000	-0.084288000
C	4.741776000	0.347126000	-0.110723000
C	2.591773000	1.474596000	-0.283261000
C	0.596673000	2.850020000	-0.482865000
N	-1.316365000	1.592061000	0.255038000
O	-1.493039000	-1.251508000	0.528811000
C	-3.353476000	0.261174000	0.096147000
C	-2.768090000	-1.047166000	0.274638000
C	-3.618197000	-2.170027000	0.241650000
C	-4.984433000	-2.055913000	0.023937000
C	-5.560902000	-0.785859000	-0.148947000
C	-4.760020000	0.335865000	-0.104072000
C	-2.628380000	1.480475000	0.170695000
C	-0.653058000	2.874932000	0.394654000
H	3.134841000	-3.156386000	-0.332210000
H	5.602148000	-2.933119000	-0.141363000
H	6.631727000	-0.653985000	-0.009042000
H	5.185256000	1.339691000	-0.061467000

H	3.168789000	2.395839000	-0.390979000
H	1.248031000	3.704974000	-0.267552000
H	0.295140000	2.899613000	-1.540476000
H	-3.157317000	-3.143258000	0.389619000
H	-5.603217000	-2.948355000	-0.007798000
H	-6.630050000	-0.685603000	-0.312608000
H	-5.203608000	1.321704000	-0.228343000
H	-3.207543000	2.406446000	0.157837000
H	-1.317714000	3.701072000	0.117725000
H	-0.349829000	3.012136000	1.445004000

$[Ru(salen)CN]^{-1}$ S_0

Ru	-0.013194000	0.076648000	-0.028544000
C	0.194627000	0.226593000	1.859840000
N	0.329091000	0.309798000	3.028616000
N	-1.346656000	1.563973000	-0.059375000
O	-1.526466000	-1.372851000	-0.018929000
C	-3.373244000	0.220736000	-0.168893000
C	-2.789794000	-1.098646000	-0.109495000
C	-3.701447000	-2.193704000	-0.168019000
C	-5.065952000	-2.023860000	-0.270643000
C	-5.626858000	-0.736302000	-0.323692000
C	-4.777774000	0.349776000	-0.273283000
C	-2.632932000	1.457808000	-0.133866000
C	-0.703048000	2.868484000	0.080962000
N	1.299692000	1.552034000	-0.359121000
O	1.484818000	-1.377710000	-0.217830000
C	3.332741000	0.216593000	-0.293847000
C	2.750303000	-1.102034000	-0.212682000
C	3.667413000	-2.191855000	-0.141301000
C	5.035210000	-2.016505000	-0.138581000
C	5.594282000	-0.729311000	-0.214620000
C	4.740338000	0.351165000	-0.297342000
C	2.585575000	1.442627000	-0.429184000
C	0.644065000	2.833722000	-0.634970000
H	-3.267594000	-3.189533000	-0.126345000
H	-5.711523000	-2.898374000	-0.310027000
H	-6.700509000	-0.598952000	-0.403403000
H	-5.191625000	1.356025000	-0.314375000
H	-3.225837000	2.377279000	-0.173655000
H	-1.329913000	3.677503000	-0.312263000
H	-0.540553000	3.045075000	1.151450000
H	3.235493000	-3.187767000	-0.084009000
H	5.685089000	-2.886558000	-0.076910000
H	6.670432000	-0.588300000	-0.214767000
H	5.152581000	1.356410000	-0.368707000
H	3.172107000	2.346433000	-0.624408000
H	1.269566000	3.680078000	-0.328205000
H	0.480096000	2.910288000	-1.717533000

Sum of electronic and zero-point Energies= -1063.643361
Sum of electronic and thermal Energies= -1063.624388
Sum of electronic and thermal Enthalpies= -1063.623443
Sum of electronic and thermal Free Energies= -1063.690829

$[Ru(salen)CN]^{-1}$ T_1

Ru	-0.010352000	0.079021000	0.073821000
C	0.178782000	0.346333000	2.309305000
N	0.292562000	0.603874000	3.449353000
N	-1.346547000	1.556279000	-0.121906000

O	-1.515943000	-1.352725000	0.090650000
C	-3.364429000	0.202167000	-0.250548000
C	-2.775370000	-1.105456000	-0.102967000
C	-3.663236000	-2.216950000	-0.176347000
C	-5.021370000	-2.067962000	-0.355990000
C	-5.592591000	-0.789001000	-0.486371000
C	-4.764187000	0.310625000	-0.435016000
C	-2.631344000	1.437926000	-0.247185000
C	-0.712871000	2.871817000	-0.072514000
N	1.291531000	1.533027000	-0.414952000
O	1.475699000	-1.357639000	-0.083759000
C	3.327330000	0.199208000	-0.375015000
C	2.743459000	-1.105736000	-0.190040000
C	3.645235000	-2.207238000	-0.136039000
C	5.010991000	-2.048191000	-0.225814000
C	5.576425000	-0.771012000	-0.395814000
C	4.734849000	0.316669000	-0.476328000
C	2.576510000	1.412009000	-0.542408000
C	0.633684000	2.790438000	-0.781969000
H	-3.219511000	-3.203586000	-0.073400000
H	-5.655445000	-2.950426000	-0.396284000
H	-6.662209000	-0.670897000	-0.627063000
H	-5.186587000	1.308241000	-0.539700000
H	-3.221701000	2.351039000	-0.371228000
H	-1.344199000	3.644865000	-0.526295000
H	-0.556107000	3.125956000	0.982565000
H	3.206002000	-3.192822000	-0.007249000
H	5.656017000	-2.921555000	-0.165842000
H	6.651977000	-0.646261000	-0.469588000
H	5.151979000	1.311161000	-0.623223000
H	3.151423000	2.299048000	-0.827159000
H	1.258144000	3.656138000	-0.532369000
H	0.473649000	2.788964000	-1.867519000

Sum of electronic and zero-point Energies= -1063.601584
Sum of electronic and thermal Energies= -1063.581339
Sum of electronic and thermal Enthalpies= -1063.580394
Sum of electronic and thermal Free Energies= -1063.652646

[Ru(salen)(H_2O)CN]$^{-1}$ S_0

Ru	-0.003926000	0.072534000	0.058172000
C	0.219278000	0.159899000	1.982648000
N	0.363639000	0.213469000	3.151339000
N	-1.334360000	1.561432000	0.083446000
O	-1.534815000	-1.380764000	0.131915000
C	-3.367037000	0.226681000	-0.041842000
C	-2.790634000	-1.097915000	0.029133000
C	-3.715201000	-2.185480000	-0.025400000
C	-5.077192000	-2.005460000	-0.138561000
C	-5.628315000	-0.713876000	-0.205915000
C	-4.769838000	0.365047000	-0.155646000
C	-2.621246000	1.460737000	0.011521000
C	-0.687001000	2.859407000	0.255961000
N	1.311105000	1.553947000	-0.226319000
O	1.514607000	-1.382567000	-0.120939000
C	3.351044000	0.225140000	-0.180914000
C	2.774812000	-1.098885000	-0.114449000
C	3.702380000	-2.183001000	-0.055467000
C	5.068853000	-1.999179000	-0.056949000
C	5.620064000	-0.707723000	-0.121763000

C	4.757726000	0.367616000	-0.187516000
C	2.597752000	1.450633000	-0.290855000
C	0.654546000	2.839931000	-0.471055000
H	-3.290620000	-3.184955000	0.027637000
H	-5.729404000	-2.875384000	-0.175168000
H	-6.700336000	-0.568304000	-0.293121000
H	-5.175618000	1.374569000	-0.202625000
H	-3.211579000	2.382942000	-0.002697000
H	-1.314483000	3.681356000	-0.109323000
H	-0.514368000	3.005739000	1.329532000
H	3.277578000	-3.182661000	-0.007661000
H	5.724271000	-2.866065000	-0.007749000
H	6.695286000	-0.559546000	-0.125531000
H	5.163363000	1.376492000	-0.247706000
H	3.182070000	2.362280000	-0.454960000
H	1.282232000	3.681243000	-0.154547000
H	0.480099000	2.939123000	-1.550737000
O	-0.259735000	-0.070172000	-2.247627000
H	-0.934609000	0.569919000	-2.503260000
H	0.555792000	0.256388000	-2.645304000

Sum of electronic and zero-point Energies= -1139.979239
Sum of electronic and thermal Energies= -1139.957208
Sum of electronic and thermal Enthalpies= -1139.956264
Sum of electronic and thermal Free Energies= -1140.030195

[Ru(salen)(CO)N$_3$]$^{-1}$ S$_0$

Ru	-0.064545000	-0.064848000	-0.201944000
C	-0.329107000	-0.560501000	-1.951162000
O	-0.487274000	-0.866492000	-3.062094000
N	1.331700000	2.420247000	2.649715000
N	0.798730000	1.461189000	2.261200000
N	0.240336000	0.468492000	1.905077000
N	-1.350896000	1.490727000	-0.315083000
O	-1.585623000	-1.362305000	0.456048000
C	-3.397740000	0.229306000	0.058500000
C	-2.841407000	-1.064404000	0.376954000
C	-3.782076000	-2.102258000	0.648756000
C	-5.144646000	-1.901275000	0.608073000
C	-5.678649000	-0.638507000	0.296079000
C	-4.803038000	0.393780000	0.036562000
C	-2.630616000	1.423957000	-0.191240000
C	-0.653711000	2.770996000	-0.426190000
N	1.291933000	1.343882000	-0.699515000
O	1.428570000	-1.515283000	0.096056000
C	3.290441000	0.038525000	-0.225515000
C	2.690324000	-1.236565000	0.089761000
C	3.594601000	-2.288293000	0.426146000
C	4.961031000	-2.114704000	0.456878000
C	5.537545000	-0.869299000	0.150473000
C	4.698772000	0.171968000	-0.183498000
C	2.571198000	1.225732000	-0.611857000
C	0.654286000	2.562985000	-1.186749000
H	-3.371334000	-3.078391000	0.892699000
H	-5.812229000	-2.732873000	0.821950000
H	-6.751538000	-0.478840000	0.266637000
H	-5.193430000	1.383020000	-0.195743000
H	-3.201741000	2.354865000	-0.269540000
H	-1.276003000	3.525959000	-0.919708000
H	-0.422178000	3.123526000	0.586549000

H	3.151589000	-3.251805000	0.663594000
H	5.598424000	-2.955040000	0.722544000
H	6.613646000	-0.732002000	0.173565000
H	5.122346000	1.144185000	-0.429306000
H	3.183029000	2.098903000	-0.859934000
H	1.309291000	3.434088000	-1.074633000
H	0.438876000	2.429923000	-2.254802000

Sum of electronic and zero-point Energies= -1248.145418
Sum of electronic and thermal Energies= -1248.123001
Sum of electronic and thermal Enthalpies= -1248.122056
Sum of electronic and thermal Free Energies= -1248.197999

$[Ru(salen)(CO)N_3]^{-1}$ T_1

Ru	0.018423000	-0.064137000	0.331838000
C	0.713107000	-0.306299000	2.059404000
O	1.185498000	-0.448343000	3.101446000
N	-1.345041000	2.069177000	-2.766275000
N	-0.980998000	1.113517000	-2.218519000
N	-0.612028000	0.098573000	-1.705142000
N	1.282351000	1.527730000	0.096914000
O	1.488073000	-1.348195000	-0.429912000
C	3.320262000	0.247431000	-0.266694000
C	2.754169000	-1.065353000	-0.439510000
C	3.669216000	-2.128884000	-0.677102000
C	5.032284000	-1.929367000	-0.722568000
C	5.580778000	-0.646435000	-0.548545000
C	4.723927000	0.411344000	-0.336062000
C	2.555210000	1.454133000	-0.090903000
C	0.579365000	2.806531000	0.184852000
N	-1.260111000	1.375478000	0.840109000
O	-1.429675000	-1.437544000	0.629410000
C	-3.252525000	0.130200000	0.163218000
C	-2.677559000	-1.206502000	0.241523000
C	-3.486632000	-2.317803000	-0.033339000
C	-4.823814000	-2.195545000	-0.406887000
C	-5.389211000	-0.910306000	-0.495579000
C	-4.630700000	0.206603000	-0.217095000
C	-2.568886000	1.314957000	0.475888000
C	-0.605587000	2.627457000	1.141543000
H	3.245039000	-3.119175000	-0.816651000
H	5.688882000	-2.777984000	-0.898266000
H	6.653844000	-0.491528000	-0.589462000
H	5.125976000	1.415046000	-0.214335000
H	3.117741000	2.391953000	-0.134419000
H	1.256483000	3.605705000	0.509068000
H	0.196029000	3.053511000	-0.812311000
H	-3.020700000	-3.297028000	0.050043000
H	-5.416336000	-3.079488000	-0.623101000
H	-6.431618000	-0.792718000	-0.781978000
H	-5.082038000	1.194786000	-0.282761000
H	-3.120922000	2.255115000	0.442232000
H	-1.294776000	3.474930000	1.044685000
H	-0.229041000	2.617296000	2.176329000

Sum of electronic and zero-point Energies= -1248.071930
Sum of electronic and thermal Energies= -1248.049238
Sum of electronic and thermal Enthalpies= -1248.048294
Sum of electronic and thermal Free Energies= -1248.124999

$[Ru(salen)N_3]^{-1}$ S_0

Ru	-0.047896000	-0.048983000	-0.195360000
N	0.694831000	2.392549000	2.762992000
N	0.457426000	1.377385000	2.251851000
N	0.205488000	0.292200000	1.804065000
N	-1.360598000	1.431053000	-0.430648000
O	-1.574242000	-1.455483000	0.090762000
C	-3.407032000	0.124604000	-0.243555000
C	-2.836712000	-1.178628000	0.009850000
C	-3.763811000	-2.250274000	0.175481000
C	-5.129579000	-2.074250000	0.105766000
C	-5.676865000	-0.802858000	-0.138758000
C	-4.813585000	0.259948000	-0.307627000
C	-2.652273000	1.336749000	-0.442267000
C	-0.708289000	2.737839000	-0.527467000
N	1.283080000	1.344414000	-0.716912000
O	1.447533000	-1.526055000	-0.123096000
C	3.307711000	0.031065000	-0.400028000
C	2.713125000	-1.259912000	-0.137893000
C	3.623345000	-2.331140000	0.107996000
C	4.992421000	-2.163887000	0.109445000
C	5.561688000	-0.904217000	-0.143177000
C	4.715712000	0.156697000	-0.396321000
C	2.571436000	1.226861000	-0.730670000
C	0.643612000	2.566363000	-1.214205000
H	-3.340097000	-3.233293000	0.365205000
H	-5.786587000	-2.930279000	0.242689000
H	-6.751410000	-0.659674000	-0.193235000
H	-5.217528000	1.253018000	-0.498307000
H	-3.236019000	2.246916000	-0.613588000
H	-1.328736000	3.461228000	-1.069562000
H	-0.552501000	3.116855000	0.490628000
H	3.183747000	-3.306358000	0.302080000
H	5.634846000	-3.019016000	0.308053000
H	6.638666000	-0.769153000	-0.145504000
H	5.136245000	1.139038000	-0.606322000
H	3.167573000	2.092257000	-1.038305000
H	1.276621000	3.446898000	-1.052784000
H	0.489261000	2.456155000	-2.295374000

Sum of electronic and zero-point Energies= -1134.897743
Sum of electronic and thermal Energies= -1134.877457
Sum of electronic and thermal Enthalpies= -1134.876513
Sum of electronic and thermal Free Energies= -1134.947885

$[Ru(salen)N_3]^{-1}$ T_1

Ru	0.018423000	-0.064137000	0.331838000
C	0.713107000	-0.306299000	2.059404000
O	1.185498000	-0.448343000	3.101446000
N	-1.345041000	2.069177000	-2.766275000
N	-0.980998000	1.113517000	-2.218519000
N	-0.612028000	0.098573000	-1.705142000
N	1.282351000	1.527730000	0.096914000
O	1.488073000	-1.348195000	-0.429912000
C	3.320262000	0.247431000	-0.266694000
C	2.754169000	-1.065353000	-0.439510000
C	3.669216000	-2.128884000	-0.677102000
C	5.032284000	-1.929367000	-0.722568000
C	5.580778000	-0.646435000	-0.548545000
C	4.723927000	0.411344000	-0.336062000
C	2.555210000	1.454133000	-0.090903000

C	0.579365000	2.806531000	0.184852000
N	-1.260111000	1.375478000	0.840109000
O	-1.429675000	-1.437544000	0.629410000
C	-3.252525000	0.130200000	0.163218000
C	-2.677559000	-1.206502000	0.241523000
C	-3.486632000	-2.317803000	-0.033339000
C	-4.823814000	-2.195545000	-0.406887000
C	-5.389211000	-0.910306000	-0.495579000
C	-4.630700000	0.206603000	-0.217095000
C	-2.568886000	1.314957000	0.475888000
C	-0.605587000	2.627457000	1.141543000
H	3.245039000	-3.119175000	-0.816651000
H	5.688882000	-2.777984000	-0.898266000
H	6.653844000	-0.491528000	-0.589462000
H	5.125976000	1.415046000	-0.214335000
H	3.117741000	2.391953000	-0.134419000
H	1.256483000	3.605705000	0.509068000
H	0.196029000	3.053511000	-0.812311000
H	-3.020700000	-3.297028000	0.050043000
H	-5.416336000	-3.079488000	-0.623101000
H	-6.431618000	-0.792718000	-0.781978000
H	-5.082038000	1.194786000	-0.282761000
H	-3.120922000	2.255115000	0.442232000
H	-1.294776000	3.474930000	1.044685000
H	-0.229041000	2.617296000	2.176329000

Sum of electronic and zero-point Energies= -1248.071930
Sum of electronic and thermal Energies= -1248.049238
Sum of electronic and thermal Enthalpies= -1248.048294
Sum of electronic and thermal Free Energies= -1248.124999

[Ru(salen)(H_2O)N_3]$^{-1}$ S_0

Ru	-0.046740000	-0.036130000	-0.106963000
N	-1.350625000	1.450330000	-0.348223000
O	-1.589513000	-1.398589000	0.362066000
C	-3.405784000	0.164084000	-0.112775000
C	-2.844093000	-1.123662000	0.234181000
C	-3.784903000	-2.176645000	0.452789000
C	-5.147678000	-1.999335000	0.343416000
C	-5.683707000	-0.744393000	0.005528000
C	-4.809799000	0.301370000	-0.211274000
C	-2.642541000	1.365156000	-0.350997000
C	-0.686200000	2.747447000	-0.475959000
N	1.285516000	1.333321000	-0.677807000
O	1.458792000	-1.514021000	0.033658000
C	3.313899000	0.030397000	-0.330696000
C	2.721704000	-1.248323000	-0.005291000
C	3.637824000	-2.305595000	0.282651000
C	5.006554000	-2.137577000	0.262539000
C	5.572298000	-0.890541000	-0.053625000
C	4.721691000	0.156790000	-0.345822000
C	2.574009000	1.214558000	-0.693627000
C	0.646996000	2.547307000	-1.191106000
H	-3.371629000	-3.147551000	0.715099000
H	-5.812005000	-2.842244000	0.521751000
H	-6.756111000	-0.599982000	-0.079685000
H	-5.204105000	1.283395000	-0.468876000
H	-3.220702000	2.276051000	-0.538416000
H	-1.309873000	3.472853000	-1.012284000
H	-0.498565000	3.135390000	0.533082000

H	3.201857000	-3.271522000	0.525922000
H	5.651793000	-2.982339000	0.494276000
H	6.649040000	-0.754644000	-0.072722000
H	5.138822000	1.129652000	-0.602372000
H	3.168874000	2.074501000	-1.018963000
H	1.290999000	3.426304000	-1.069455000
H	0.463923000	2.412302000	-2.265563000
N	0.964967000	2.458679000	2.791236000
N	0.606300000	1.451420000	2.331010000
N	0.226928000	0.392359000	1.924627000
O	-0.373846000	-0.648897000	-2.225024000
H	-1.017170000	-0.042878000	-2.613797000
H	0.444731000	-0.496491000	-2.712745000

Sum of electronic and zero-point Energies= -1211.239589
Sum of electronic and thermal Energies= -1211.216459
Sum of electronic and thermal Enthalpies= -1211.215515
Sum of electronic and thermal Free Energies= -1211.292655

$[Ru(salen)(CO)NO_3]^{-1}$ S_0

Ru	-0.064545000	-0.064848000	-0.201944000
C	-0.329107000	-0.560501000	-1.951162000
O	-0.487274000	-0.866492000	-3.062094000
N	1.331700000	2.420247000	2.649715000
N	0.798730000	1.461189000	2.261200000
N	0.240336000	0.468492000	1.905077000
N	-1.350896000	1.490727000	-0.315083000
O	-1.585623000	-1.362305000	0.456048000
C	-3.397740000	0.229306000	0.058500000
C	-2.841407000	-1.064404000	0.376954000
C	-3.782076000	-2.102258000	0.648756000
C	-5.144646000	-1.901275000	0.608073000
C	-5.678649000	-0.638507000	0.296079000
C	-4.803038000	0.393780000	0.036562000
C	-2.630616000	1.423957000	-0.191240000
C	-0.653711000	2.770996000	-0.426190000
N	1.291933000	1.343882000	-0.699515000
O	1.428570000	-1.515283000	0.096056000
C	3.290441000	0.038525000	-0.225515000
C	2.690324000	-1.236565000	0.089761000
C	3.594601000	-2.288293000	0.426146000
C	4.961031000	-2.114704000	0.456878000
C	5.537545000	-0.869299000	0.150473000
C	4.698772000	0.171968000	-0.183498000
C	2.571198000	1.225732000	-0.611857000
C	0.654286000	2.562985000	-1.186749000
H	-3.371334000	-3.078391000	0.892699000
H	-5.812229000	-2.732873000	0.821950000
H	-6.751538000	-0.478840000	0.266637000
H	-5.193430000	1.383020000	-0.195743000
H	-3.201741000	2.354865000	-0.269540000
H	-1.276003000	3.525959000	-0.919708000
H	-0.422178000	3.123526000	0.586549000
H	3.151589000	-3.251805000	0.663594000
H	5.598424000	-2.955040000	0.722544000
H	6.613646000	-0.732002000	0.173565000
H	5.122346000	1.144185000	-0.429306000
H	3.183029000	2.098903000	-0.859934000
H	1.309291000	3.434088000	-1.074633000
H	0.438876000	2.429923000	-2.254802000

Sum of electronic and zero-point Energies=	-1248.145418
Sum of electronic and thermal Energies=	-1248.123001
Sum of electronic and thermal Enthalpies=	-1248.122056
Sum of electronic and thermal Free Energies=	-1248.197999

$[Ru(salen)(CO)NO_3]^{-1}$ T_1

Ru	0.018423000	-0.064137000	0.331838000
C	0.713107000	-0.306299000	2.059404000
O	1.185498000	-0.448343000	3.101446000
N	-1.345041000	2.069177000	-2.766275000
N	-0.980998000	1.113517000	-2.218519000
N	-0.612028000	0.098573000	-1.705142000
N	1.282351000	1.527730000	0.096914000
O	1.488073000	-1.348195000	-0.429912000
C	3.320262000	0.247431000	-0.266694000
C	2.754169000	-1.065353000	-0.439510000
C	3.669216000	-2.128884000	-0.677102000
C	5.032284000	-1.929367000	-0.722568000
C	5.580778000	-0.646435000	-0.548545000
C	4.723927000	0.411344000	-0.336062000
C	2.555210000	1.454133000	-0.090903000
C	0.579365000	2.806531000	0.184852000
N	-1.260111000	1.375478000	0.840109000
O	-1.429675000	-1.437544000	0.629410000
C	-3.252525000	0.130200000	0.163218000
C	-2.677559000	-1.206502000	0.241523000
C	-3.486632000	-2.317803000	-0.033339000
C	-4.823814000	-2.195545000	-0.406887000
C	-5.389211000	-0.910306000	-0.495579000
C	-4.630700000	0.206603000	-0.217095000
C	-2.568886000	1.314957000	0.475888000
C	-0.605587000	2.627457000	1.141543000
H	3.245039000	-3.119175000	-0.816651000
H	5.688882000	-2.777984000	-0.898266000
H	6.653844000	-0.491528000	-0.589462000
H	5.125976000	1.415046000	-0.214335000
H	3.117741000	2.391953000	-0.134419000
H	1.256483000	3.605705000	0.509068000
H	0.196029000	3.053511000	-0.812311000
H	-3.020700000	-3.297028000	0.050043000
H	-5.416336000	-3.079488000	-0.623101000
H	-6.431618000	-0.792718000	-0.781978000
H	-5.082038000	1.194786000	-0.282761000
H	-3.120922000	2.255115000	0.442232000
H	-1.294776000	3.474930000	1.044685000
H	-0.229041000	2.617296000	2.176329000

Sum of electronic and zero-point Energies=	-1248.071930
Sum of electronic and thermal Energies=	-1248.049238
Sum of electronic and thermal Enthalpies=	-1248.048294
Sum of electronic and thermal Free Energies=	-1248.124999

$[Ru(salen)NO_3]^{-1}$ S_0

Ru	-0.047896000	-0.048983000	-0.195360000
N	0.694831000	2.392549000	2.762992000
N	0.457426000	1.377385000	2.251851000
N	0.205488000	0.292200000	1.804065000
N	-1.360598000	1.431053000	-0.430648000
O	-1.574242000	-1.455483000	0.090762000
C	-3.407032000	0.124604000	-0.243555000

C	-2.836712000	-1.178628000	0.009850000
C	-3.763811000	-2.250274000	0.175481000
C	-5.129579000	-2.074250000	0.105766000
C	-5.676865000	-0.802858000	-0.138758000
C	-4.813585000	0.259948000	-0.307627000
C	-2.652273000	1.336749000	-0.442267000
C	-0.708289000	2.737839000	-0.527467000
N	1.283080000	1.344414000	-0.716912000
O	1.447533000	-1.526055000	-0.123096000
C	3.307711000	0.031065000	-0.400028000
C	2.713125000	-1.259912000	-0.137893000
C	3.623345000	-2.331140000	0.107996000
C	4.992421000	-2.163887000	0.109445000
C	5.561688000	-0.904217000	-0.143177000
C	4.715712000	0.156697000	-0.396321000
C	2.571436000	1.226861000	-0.730670000
C	0.643612000	2.566363000	-1.214205000
H	-3.340097000	-3.233293000	0.365205000
H	-5.786587000	-2.930279000	0.242689000
H	-6.751410000	-0.659674000	-0.193235000
H	-5.217528000	1.253018000	-0.498307000
H	-3.236019000	2.246916000	-0.613588000
H	-1.328736000	3.461228000	-1.069562000
H	-0.552501000	3.116855000	0.490628000
H	3.183747000	-3.306358000	0.302080000
H	5.634846000	-3.019016000	0.308053000
H	6.638666000	-0.769153000	-0.145504000
H	5.136245000	1.139038000	-0.606322000
H	3.167573000	2.092257000	-1.038305000
H	1.276621000	3.446898000	-1.052784000
H	0.489261000	2.456155000	-2.295374000

Sum of electronic and zero-point Energies= -1134.897743
Sum of electronic and thermal Energies= -1134.877457
Sum of electronic and thermal Enthalpies= -1134.876513
Sum of electronic and thermal Free Energies= -1134.947885

$[Ru(salen)NO_3]^{-1}$ T_1

Ru	0.003063000	-0.058498000	-0.125980000
N	-0.177925000	2.808973000	2.705148000
N	0.002945000	1.689243000	2.420169000
N	0.187857000	0.543833000	2.142694000
N	-1.329924000	1.376274000	-0.536128000
O	-1.487972000	-1.455250000	0.191474000
C	-3.349854000	0.031440000	-0.337684000
C	-2.755740000	-1.234533000	0.011890000
C	-3.645530000	-2.334648000	0.172479000
C	-5.010101000	-2.210502000	0.024644000
C	-5.586168000	-0.970246000	-0.304449000
C	-4.756238000	0.115407000	-0.480086000
C	-2.619159000	1.245383000	-0.580323000
C	-0.699132000	2.682054000	-0.733922000
N	1.306265000	1.305787000	-0.801683000
O	1.491802000	-1.477994000	0.063442000
C	3.344680000	0.026515000	-0.432966000
C	2.761657000	-1.228901000	-0.029285000
C	3.668035000	-2.283262000	0.280808000
C	5.035162000	-2.123257000	0.223011000
C	5.599252000	-0.893944000	-0.165680000
C	4.755123000	0.144506000	-0.490976000

C	2.596931000	1.180101000	-0.846908000
C	0.655622000	2.474629000	-1.400756000
H	-3.197527000	-3.291111000	0.428363000
H	-5.644883000	-3.082099000	0.165319000
H	-6.660655000	-0.870787000	-0.420445000
H	-5.182851000	1.082294000	-0.740448000
H	-3.217202000	2.126277000	-0.833622000
H	-1.329402000	3.347985000	-1.335221000
H	-0.556733000	3.133622000	0.256296000
H	3.229811000	-3.231941000	0.578868000
H	5.682644000	-2.958160000	0.480152000
H	6.676204000	-0.768700000	-0.213872000
H	5.171886000	1.099513000	-0.805482000
H	3.180784000	2.012510000	-1.252447000
H	1.277555000	3.372221000	-1.303642000
H	0.513174000	2.275031000	-2.470215000

Sum of electronic and zero-point Energies= -1134.892954
Sum of electronic and thermal Energies= -1134.872191
Sum of electronic and thermal Enthalpies= -1134.871246
Sum of electronic and thermal Free Energies= -1134.945144

[Ru(salen)(H_2O)NO_3]$^{-1}$ S_0

Ru	-0.046740000	-0.036130000	-0.106963000
N	-1.350625000	1.450330000	-0.348223000
O	-1.589513000	-1.398589000	0.362066000
C	-3.405784000	0.164084000	-0.112775000
C	-2.844093000	-1.123662000	0.234181000
C	-3.784903000	-2.176645000	0.452789000
C	-5.147678000	-1.999335000	0.343416000
C	-5.683707000	-0.744393000	0.005528000
C	-4.809799000	0.301370000	-0.211274000
C	-2.642541000	1.365156000	-0.350997000
C	-0.686200000	2.747447000	-0.475959000
N	1.285516000	1.333321000	-0.677807000
O	1.458792000	-1.514021000	0.033658000
C	3.313899000	0.030397000	-0.330696000
C	2.721704000	-1.248323000	-0.005291000
C	3.637824000	-2.305595000	0.282651000
C	5.006554000	-2.137577000	0.262539000
C	5.572298000	-0.890541000	-0.053625000
C	4.721691000	0.156790000	-0.345822000
C	2.574009000	1.214558000	-0.693627000
C	0.646996000	2.547307000	-1.191106000
H	-3.371629000	-3.147551000	0.715099000
H	-5.812005000	-2.842244000	0.521751000
H	-6.756111000	-0.599982000	-0.079685000
H	-5.204105000	1.283395000	-0.468876000
H	-3.220702000	2.276051000	-0.538416000
H	-1.309873000	3.472853000	-1.012284000
H	-0.498565000	3.135390000	0.533082000
H	3.201857000	-3.271522000	0.525922000
H	5.651793000	-2.982339000	0.494276000
H	6.649040000	-0.754644000	-0.072722000
H	5.138822000	1.129652000	-0.602372000
H	3.168874000	2.074501000	-1.018963000
H	1.290999000	3.426304000	-1.069455000
H	0.463923000	2.412302000	-2.265563000
N	0.964967000	2.458679000	2.791236000
N	0.606300000	1.451420000	2.331010000

N	0.226928000	0.392359000	1.924627000
O	-0.373846000	-0.648897000	-2.225024000
H	-1.017170000	-0.042878000	-2.613797000
H	0.444731000	-0.496491000	-2.712745000

Sum of electronic and zero-point Energies= -1211.239589
Sum of electronic and thermal Energies= -1211.216459
Sum of electronic and thermal Enthalpies= -1211.215515
Sum of electronic and thermal Free Energies= -1211.292655

$[Ru(salen)(CO)CNH]^0$ S_0

Ru	-0.003624000	0.075795000	-0.055573000
C	-0.231300000	0.025651000	-1.949426000
O	-0.370229000	-0.047900000	-3.088718000
C	0.240865000	0.003418000	1.971224000
N	0.377958000	-0.077093000	3.124469000
H	0.494923000	-0.160114000	4.115133000
N	-1.329740000	1.593474000	0.166281000
O	-1.525686000	-1.374580000	0.143476000
C	-3.356866000	0.248926000	0.095015000
C	-2.785314000	-1.075792000	0.090809000
C	-3.709006000	-2.160347000	0.039299000
C	-5.073038000	-1.972047000	-0.010775000
C	-5.623838000	-0.678403000	-0.008082000
C	-4.764283000	0.396549000	0.050111000
C	-2.611786000	1.478328000	0.193247000
C	-0.657760000	2.869344000	0.398799000
N	1.330343000	1.596748000	-0.181228000
O	1.524082000	-1.376703000	-0.166531000
C	3.354156000	0.248092000	-0.078065000
C	2.782545000	-1.076612000	-0.099516000
C	3.705839000	-2.161605000	-0.046987000
C	5.069036000	-1.974328000	0.024416000
C	5.619845000	-0.680954000	0.046401000
C	4.760716000	0.394537000	-0.007997000
C	2.612128000	1.481044000	-0.154980000
C	0.665019000	2.883211000	-0.364281000
H	-3.285170000	-3.160825000	0.039273000
H	-5.728498000	-2.838802000	-0.051960000
H	-6.698018000	-0.530275000	-0.045099000
H	-5.168120000	1.407102000	0.063665000
H	-3.202916000	2.392049000	0.309949000
H	-1.286971000	3.715210000	0.101371000
H	-0.453307000	2.954559000	1.474010000
H	3.282078000	-3.161950000	-0.064938000
H	5.723692000	-2.841786000	0.063711000
H	6.693323000	-0.533242000	0.100960000
H	5.164579000	1.405133000	0.000772000
H	3.207044000	2.398409000	-0.203586000
H	1.295821000	3.714041000	-0.030071000
H	0.464014000	3.012985000	-1.435706000

Sum of electronic and zero-point Energies= -1177.298750
Sum of electronic and thermal Energies= -1177.276619
Sum of electronic and thermal Enthalpies= -1177.275675
Sum of electronic and thermal Free Energies= -1177.350177

$[Ru(salen)(CO)CNH]^0$ T_1

Ru	0.003634000	0.070340000	-0.045265000
C	-0.643651000	0.086955000	-1.888388000
O	-1.064214000	0.105744000	-2.950728000

C	0.617691000	0.015209000	1.896532000
N	0.962379000	-0.017482000	3.005111000
H	1.264691000	-0.044957000	3.959767000
N	-1.294685000	1.592527000	0.248937000
O	-1.452456000	-1.267227000	0.463709000
C	-3.333196000	0.253469000	0.156789000
C	-2.741139000	-1.058168000	0.284396000
C	-3.589648000	-2.181872000	0.272485000
C	-4.964052000	-2.064294000	0.124428000
C	-5.548367000	-0.792100000	-0.001108000
C	-4.748045000	0.329750000	0.022767000
C	-2.611015000	1.474591000	0.237535000
C	-0.622932000	2.860944000	0.464712000
N	1.301539000	1.598436000	-0.236027000
O	1.468304000	-1.252752000	-0.537046000
C	3.338501000	0.259231000	-0.138374000
C	2.750799000	-1.047418000	-0.317474000
C	3.600251000	-2.171211000	-0.320203000
C	4.969458000	-2.057471000	-0.132252000
C	5.549369000	-0.789235000	0.044185000
C	4.748832000	0.332767000	0.031063000
C	2.616120000	1.481884000	-0.198700000
C	0.633301000	2.876103000	-0.404277000
H	-3.121405000	-3.156603000	0.379508000
H	-5.583885000	-2.956214000	0.110237000
H	-6.624018000	-0.690929000	-0.111623000
H	-5.197389000	1.316620000	-0.064037000
H	-3.192830000	2.396725000	0.292970000
H	-1.279242000	3.705348000	0.227236000
H	-0.329268000	2.938319000	1.523048000
H	3.136007000	-3.142260000	-0.469222000
H	5.589635000	-2.949222000	-0.126845000
H	6.621737000	-0.691128000	0.184704000
H	5.195055000	1.316975000	0.155454000
H	3.197393000	2.405739000	-0.212651000
H	1.292176000	3.708976000	-0.135724000
H	0.340083000	2.992318000	-1.459499000

Sum of electronic and zero-point Energies= -1177.209525
Sum of electronic and thermal Energies= -1177.186995
Sum of electronic and thermal Enthalpies= -1177.186051
Sum of electronic and thermal Free Energies= -1177.261990

$[Ru(salen)CNH]^0$ S_0

Ru	-0.011972000	0.082509000	-0.007054000
C	0.156917000	0.188257000	1.778421000
N	0.398162000	0.274357000	2.956942000
N	-1.351949000	1.586435000	-0.076865000
O	-1.490419000	-1.358047000	-0.115694000
C	-3.359325000	0.217232000	-0.192766000
C	-2.763088000	-1.093063000	-0.164935000
C	-3.655481000	-2.199497000	-0.217335000
C	-5.023413000	-2.042678000	-0.285065000
C	-5.601426000	-0.761690000	-0.309285000
C	-4.768146000	0.334546000	-0.266424000
C	-2.633287000	1.459353000	-0.166019000
C	-0.709171000	2.893535000	0.042288000
N	1.299655000	1.573433000	-0.365407000
O	1.453990000	-1.360960000	-0.242054000

C	3.317298000	0.216351000	-0.308441000
C	2.725741000	-1.094783000	-0.229891000
C	3.625349000	-2.195394000	-0.163092000
C	4.993649000	-2.030761000	-0.161402000
C	5.566367000	-0.748512000	-0.235023000
C	4.727705000	0.341004000	-0.315255000
C	2.581698000	1.443182000	-0.453025000
C	0.638586000	2.846062000	-0.670572000
H	-0.180961000	0.143302000	3.772999000
H	-3.208335000	-3.189447000	-0.199460000
H	-5.659448000	-2.923828000	-0.319537000
H	-6.678121000	-0.638673000	-0.362069000
H	-5.193968000	1.335675000	-0.287333000
H	-3.234711000	2.371504000	-0.223704000
H	-1.336053000	3.691206000	-0.370401000
H	-0.553734000	3.093897000	1.109700000
H	3.183079000	-3.186237000	-0.109183000
H	5.635486000	-2.906471000	-0.103365000
H	6.643832000	-0.620874000	-0.236497000
H	5.149115000	1.341656000	-0.387015000
H	3.172354000	2.337991000	-0.672270000
H	1.262094000	3.698677000	-0.380836000
H	0.476664000	2.894834000	-1.754434000

Sum of electronic and zero-point Energies= -1064.092328
Sum of electronic and thermal Energies= -1064.072666
Sum of electronic and thermal Enthalpies= -1064.071722
Sum of electronic and thermal Free Energies= -1064.140542

[Ru(salen)CNH]^0 T_1

Ru	-0.078614000	0.062542000	0.053276000
C	0.658765000	0.163529000	2.060364000
N	1.504266000	0.338156000	2.857238000
N	-1.401934000	1.579156000	-0.032141000
O	-1.596863000	-1.301288000	0.305178000
C	-3.425534000	0.236337000	-0.167452000
C	-2.850713000	-1.064370000	0.053593000
C	-3.738913000	-2.174376000	0.020360000
C	-5.089033000	-2.026198000	-0.212278000
C	-5.648791000	-0.752570000	-0.421667000
C	-4.819495000	0.346264000	-0.391670000
C	-2.687180000	1.466831000	-0.154015000
C	-0.756787000	2.887593000	0.040911000
N	1.225624000	1.550079000	-0.402520000
O	1.366265000	-1.321504000	-0.406437000
C	3.251613000	0.211712000	-0.401229000
C	2.648325000	-1.092182000	-0.387201000
C	3.524891000	-2.209370000	-0.392388000
C	4.896301000	-2.061942000	-0.377846000
C	5.483647000	-0.784803000	-0.384105000
C	4.661453000	0.321170000	-0.410746000
C	2.508720000	1.437252000	-0.517153000
C	0.557817000	2.812759000	-0.727141000
H	2.278720000	0.509272000	3.467386000
H	-3.305253000	-3.156013000	0.188916000
H	-5.727140000	-2.906160000	-0.230601000
H	-6.712959000	-0.638332000	-0.600132000
H	-5.233838000	1.340253000	-0.546605000
H	-3.273544000	2.385368000	-0.249740000
H	-1.398741000	3.677492000	-0.364783000

H	-0.557098000	3.109933000	1.095873000
H	3.069063000	-3.195401000	-0.395014000
H	5.527754000	-2.946773000	-0.365418000
H	6.562864000	-0.672162000	-0.379734000
H	5.096044000	1.318227000	-0.437973000
H	3.085914000	2.335469000	-0.756206000
H	1.197540000	3.671671000	-0.496343000
H	0.348261000	2.820109000	-1.803768000

Sum of electronic and zero-point Energies= -1064.042463
Sum of electronic and thermal Energies= -1064.021739
Sum of electronic and thermal Enthalpies= -1064.020795
Sum of electronic and thermal Free Energies= -1064.093205

[Ru(salen)(H_2O)CNH]^0 S_0

Ru	-0.013703000	0.095178000	0.062538000
C	0.140132000	0.218256000	1.882001000
N	0.132987000	0.296572000	3.080362000
N	-1.346554000	1.599983000	0.001875000
O	-1.504343000	-1.355326000	0.115769000
C	-3.355819000	0.230288000	-0.088872000
C	-2.767448000	-1.082642000	0.016368000
C	-3.673087000	-2.183064000	-0.001858000
C	-5.036965000	-2.020153000	-0.111140000
C	-5.604576000	-0.737669000	-0.212116000
C	-4.761976000	0.352213000	-0.199393000
C	-2.627159000	1.472101000	-0.081672000
C	-0.702486000	2.904651000	0.127634000
N	1.307476000	1.585427000	-0.279211000
O	1.468153000	-1.363980000	-0.197625000
C	3.324997000	0.227283000	-0.184324000
C	2.741526000	-1.087624000	-0.135912000
C	3.644383000	-2.180817000	-0.041382000
C	5.012349000	-2.008728000	0.009611000
C	5.577479000	-0.723947000	-0.037817000
C	4.732675000	0.360608000	-0.142162000
C	2.588851000	1.457919000	-0.341621000
C	0.650111000	2.860505000	-0.578084000
H	0.824724000	0.267596000	3.811847000
H	-3.235973000	-3.174797000	0.076202000
H	-5.679018000	-2.897829000	-0.118569000
H	-6.678618000	-0.609141000	-0.296970000
H	-5.179209000	1.354613000	-0.274653000
H	-3.228231000	2.384449000	-0.144998000
H	-1.326433000	3.705362000	-0.284285000
H	-0.550424000	3.101748000	1.196210000
H	3.207034000	-3.174849000	-0.008406000
H	5.656671000	-2.881153000	0.086636000
H	6.653284000	-0.587711000	-0.000610000
H	5.149839000	1.364362000	-0.193660000
H	3.185528000	2.353787000	-0.539798000
H	1.272768000	3.710933000	-0.279932000
H	0.493787000	2.921586000	-1.662506000
O	-0.072859000	-0.296856000	-2.193213000
H	0.374663000	0.385242000	-2.708187000
H	0.579650000	-1.011687000	-2.106169000

Sum of electronic and zero-point Energies= -1140.431447
Sum of electronic and thermal Energies= -1140.409208
Sum of electronic and thermal Enthalpies= -1140.408264
Sum of electronic and thermal Free Energies= -1140.481890

[Ru(salen)(CO)NHC]⁰ S₀

Ru	0.045932000	-0.412602000	-0.055173000
C	0.358192000	-2.111241000	-0.860363000
O	0.551239000	-3.123493000	-1.385926000
C	-0.267923000	1.534830000	0.662887000
N	0.392018000	2.585941000	0.144931000
N	-1.099231000	2.095001000	1.565778000
C	-0.965982000	3.469093000	1.609822000
C	-0.009444000	3.781605000	0.698298000
H	-1.747992000	1.561017000	2.123170000
H	1.084274000	2.424118000	-0.579269000
H	-1.547372000	4.091336000	2.270978000
H	0.407015000	4.733050000	0.408753000
N	1.348568000	-0.756264000	1.453771000
O	1.577277000	0.505127000	-1.215641000
C	3.393643000	-0.265674000	0.231333000
C	2.840782000	0.294309000	-0.974243000
C	3.771661000	0.680149000	-1.977602000
C	5.134388000	0.523890000	-1.826202000
C	5.667933000	-0.024846000	-0.649306000
C	4.796011000	-0.397740000	0.352551000
C	2.629713000	-0.663695000	1.391794000
C	0.658343000	-1.060969000	2.704235000
N	-1.294588000	-1.147047000	1.261473000
O	-1.472000000	0.041665000	-1.458754000
C	-3.314635000	-0.564195000	0.032809000
C	-2.731693000	-0.042312000	-1.181609000
C	-3.651648000	0.419537000	-2.170129000
C	-5.017297000	0.379940000	-1.993045000
C	-5.577246000	-0.128634000	-0.807484000
C	-4.723386000	-0.588113000	0.171403000
C	-2.578162000	-1.103114000	1.148624000
C	-0.638475000	-1.803528000	2.389024000
H	3.359260000	1.108090000	-2.887357000
H	5.798599000	0.831795000	-2.630243000
H	6.739224000	-0.146141000	-0.526780000
H	5.188518000	-0.812523000	1.279056000
H	3.206795000	-0.903844000	2.290362000
H	1.289507000	-1.647471000	3.381007000
H	0.418204000	-0.110123000	3.196787000
H	-3.221610000	0.812078000	-3.087693000
H	-5.667313000	0.747864000	-2.783618000
H	-6.652793000	-0.161127000	-0.668058000
H	-5.134057000	-0.991294000	1.095388000
H	-3.177263000	-1.514802000	1.967107000
H	-1.288216000	-1.839356000	3.270542000
H	-0.403944000	-2.833700000	2.091562000

Sum of electronic and zero-point Energies= -1309.908658
Sum of electronic and thermal Energies= -1309.884976
Sum of electronic and thermal Enthalpies= -1309.884031
Sum of electronic and thermal Free Energies= -1309.962473

[Ru(salen)(CO)NHC]⁰ T₁

Ru	0.080625000	-0.371548000	-0.061867000
C	0.800639000	-2.034522000	-0.840895000
O	1.287624000	-2.965348000	-1.296218000
C	-0.523253000	1.471448000	0.669082000
N	0.092402000	2.616309000	0.332290000

N	-1.527186000	1.861712000	1.474859000
C	-1.547215000	3.230955000	1.638297000
C	-0.508823000	3.712987000	0.906608000
H	-2.188095000	1.212200000	1.876138000
H	0.893825000	2.588349000	-0.289130000
H	-2.280282000	3.734183000	2.247796000
H	-0.157714000	4.720886000	0.754837000
N	1.349442000	-0.685666000	1.480981000
O	1.534521000	0.733561000	-1.001540000
C	3.408504000	-0.163419000	0.276102000
C	2.831596000	0.492171000	-0.873020000
C	3.688594000	0.953403000	-1.886095000
C	5.066945000	0.788233000	-1.818695000
C	5.638956000	0.154983000	-0.702979000
C	4.825524000	-0.299115000	0.313695000
C	2.668299000	-0.616121000	1.401680000
C	0.660573000	-1.042617000	2.706507000
N	-1.224466000	-1.204533000	1.223394000
O	-1.364129000	-0.276728000	-1.491341000
C	-3.262181000	-0.684531000	-0.019623000
C	-2.656242000	-0.235097000	-1.249776000
C	-3.502880000	0.211073000	-2.285037000
C	-4.881904000	0.247576000	-2.144613000
C	-5.478186000	-0.193042000	-0.950943000
C	-4.680532000	-0.653132000	0.075073000
C	-2.537634000	-1.220940000	1.082794000
C	-0.559583000	-1.877270000	2.327285000
H	3.228704000	1.449688000	-2.736817000
H	5.695287000	1.151982000	-2.626738000
H	6.715433000	0.025231000	-0.637280000
H	5.265914000	-0.784141000	1.182436000
H	3.237582000	-0.940150000	2.275562000
H	1.317617000	-1.592099000	3.390194000
H	0.324230000	-0.125626000	3.215008000
H	-3.028051000	0.533276000	-3.207911000
H	-5.497606000	0.612175000	-2.962114000
H	-6.557973000	-0.175829000	-0.836717000
H	-5.136262000	-1.004343000	0.998478000
H	-3.118447000	-1.682724000	1.884008000
H	-1.235117000	-2.006197000	3.180286000
H	-0.229184000	-2.873505000	1.993572000

Sum of electronic and zero-point Energies= -1309.826528

Sum of electronic and thermal Energies= -1309.802461

Sum of electronic and thermal Enthalpies= -1309.801517

Sum of electronic and thermal Free Energies= -1309.881283

$[Ru(salen)NHC]^0$ S_0

Ru	-0.022921000	0.171364000	-0.400227000
C	0.112953000	-0.184898000	1.487476000
N	-0.502044000	-1.219888000	2.111886000
N	0.800192000	0.426932000	2.485716000
C	0.618595000	-0.211626000	3.699718000
C	-0.211253000	-1.255903000	3.460965000
N	-1.339960000	1.656934000	-0.140434000
O	-1.555302000	-1.261668000	-0.547160000
C	-3.380192000	0.365333000	-0.461962000
C	-2.819391000	-0.956745000	-0.592428000
C	-3.745203000	-2.020140000	-0.790566000
C	-5.107771000	-1.818103000	-0.862999000

C	-5.648188000	-0.527374000	-0.740219000
C	-4.782971000	0.528523000	-0.542245000
C	-2.626719000	1.574083000	-0.228908000
C	-0.688038000	2.914731000	0.220729000
N	1.309789000	1.664289000	-0.406403000
O	1.449646000	-1.228509000	-0.884899000
C	3.323459000	0.312699000	-0.599797000
C	2.720572000	-0.982948000	-0.803314000
C	3.617844000	-2.080981000	-0.946722000
C	4.987765000	-1.935454000	-0.888035000
C	5.567851000	-0.671015000	-0.687324000
C	4.733355000	0.419076000	-0.555520000
C	2.596980000	1.553143000	-0.493193000
C	0.671618000	2.984104000	-0.468135000
H	-1.107887000	-1.838867000	1.590723000
H	1.391796000	1.227593000	2.331172000
H	1.087446000	0.127865000	4.609156000
H	-0.609817000	-2.007059000	4.123748000
H	-3.326041000	-3.018365000	-0.886713000
H	-5.766358000	-2.669658000	-1.017432000
H	-6.719609000	-0.364094000	-0.796493000
H	-5.181752000	1.536204000	-0.439010000
H	-3.212815000	2.491213000	-0.111614000
H	-1.301529000	3.782931000	-0.046603000
H	-0.549244000	2.920401000	1.309449000
H	3.169530000	-3.058602000	-1.104048000
H	5.623005000	-2.811228000	-0.999332000
H	6.645886000	-0.553718000	-0.643506000
H	5.162366000	1.409313000	-0.411924000
H	3.196529000	2.468987000	-0.504087000
H	1.300529000	3.757843000	-0.012925000
H	0.528309000	3.243757000	-1.524568000

Sum of electronic and zero-point Energies= -1196.688213
Sum of electronic and thermal Energies= -1196.666739
Sum of electronic and thermal Enthalpies= -1196.665795
Sum of electronic and thermal Free Energies= -1196.739160

[Ru(salen)NHC]^0 T_1

Ru	-0.028334000	0.396328000	-0.164594000
C	0.194944000	-1.352613000	1.255809000
N	-0.548760000	-2.454003000	1.023536000
N	0.930535000	-1.706306000	2.332732000
C	0.658646000	-2.991291000	2.760976000
C	-0.293940000	-3.469615000	1.919739000
N	-1.343166000	1.498546000	0.865794000
O	-1.560631000	-0.706431000	-1.042424000
C	-3.383084000	0.661191000	-0.166398000
C	-2.820745000	-0.369993000	-0.998026000
C	-3.724006000	-1.094155000	-1.822547000
C	-5.078968000	-0.838133000	-1.835352000
C	-5.626109000	0.165460000	-1.017175000
C	-4.780317000	0.887262000	-0.203287000
C	-2.633942000	1.489984000	0.739044000
C	-0.697372000	2.313400000	1.894446000
N	1.296053000	1.661155000	0.657306000
O	1.435884000	-0.351942000	-1.424070000
C	3.318226000	0.719725000	-0.312088000
C	2.712435000	-0.190834000	-1.249433000
C	3.596923000	-0.953954000	-2.062657000

C	4.967589000	-0.861199000	-1.948660000
C	5.554188000	0.022157000	-1.024554000
C	4.729513000	0.797706000	-0.238822000
C	2.584640000	1.639576000	0.512351000
C	0.658358000	2.771296000	1.371467000
H	-1.201169000	-2.466938000	0.248659000
H	1.602182000	-1.090055000	2.763358000
H	1.151182000	-3.445677000	3.606008000
H	-0.795775000	-4.423719000	1.890195000
H	-3.297723000	-1.872007000	-2.450276000
H	-5.727385000	-1.421576000	-2.484354000
H	-6.692606000	0.366127000	-1.023815000
H	-5.186085000	1.665549000	0.440043000
H	-3.217259000	2.167403000	1.369844000
H	-1.315627000	3.173644000	2.176394000
H	-0.556964000	1.684840000	2.782099000
H	3.141945000	-1.632412000	-2.779234000
H	5.599476000	-1.477554000	-2.583604000
H	6.633356000	0.098168000	-0.938645000
H	5.163964000	1.500782000	0.469324000
H	3.174013000	2.400665000	1.032969000
H	1.290078000	3.135300000	2.189964000
H	0.514555000	3.594420000	0.660570000

Sum of electronic and zero-point Energies= -1196.640609
Sum of electronic and thermal Energies= -1196.618367
Sum of electronic and thermal Enthalpies= -1196.617423
Sum of electronic and thermal Free Energies= -1196.695263

[Ru(salen)(H_2O)NHC]^0 S_0

Ru	0.029776000	-0.299447000	-0.162690000
C	-0.181537000	1.094085000	1.194984000
N	0.429859000	2.302212000	1.133349000
N	-0.920427000	1.175527000	2.331053000
C	-0.773361000	2.399951000	2.958038000
C	0.089048000	3.115324000	2.194950000
N	1.343446000	-1.334135000	0.933182000
O	1.571457000	0.873350000	-1.018275000
C	3.388155000	-0.450161000	-0.053437000
C	2.831225000	0.581183000	-0.895413000
C	3.763741000	1.346784000	-1.653199000
C	5.124483000	1.124451000	-1.608534000
C	5.659117000	0.114151000	-0.792284000
C	4.788894000	-0.642778000	-0.034735000
C	2.631132000	-1.308519000	0.828279000
C	0.684342000	-2.152354000	1.949081000
N	-1.300084000	-1.532621000	0.676391000
O	-1.461677000	0.591985000	-1.353950000
C	-3.322341000	-0.548364000	-0.255693000
C	-2.727752000	0.416318000	-1.152440000
C	-3.637951000	1.230575000	-1.890388000
C	-5.006935000	1.122058000	-1.766423000
C	-5.576887000	0.181116000	-0.891547000
C	-4.731256000	-0.631171000	-0.164692000
C	-2.587425000	-1.496954000	0.544787000
C	-0.657678000	-2.631884000	1.402614000
H	1.067355000	2.499782000	0.372880000
H	-1.518336000	0.428464000	2.646592000
H	-1.284706000	2.649844000	3.873733000
H	0.477728000	4.113704000	2.316598000

H	3.350125000	2.128221000	-2.285622000
H	5.786128000	1.739852000	-2.213930000
H	6.728993000	-0.063864000	-0.753204000
H	5.183244000	-1.424554000	0.612406000
H	3.215336000	-1.982046000	1.463569000
H	1.305335000	-3.002999000	2.254002000
H	0.515557000	-1.520837000	2.830739000
H	-3.198568000	1.957151000	-2.569262000
H	-5.649451000	1.773551000	-2.354614000
H	-6.654074000	0.091647000	-0.793596000
H	-5.151640000	-1.373829000	0.511680000
H	-3.182378000	-2.249855000	1.071838000
H	-1.295120000	-3.010509000	2.210125000
H	-0.486275000	-3.454528000	0.696293000
O	0.340845000	-1.816203000	-1.890512000
H	0.956499000	-2.497417000	-1.594914000
H	-0.492545000	-2.278888000	-2.036571000

Sum of electronic and zero-point Energies= -1273.024884
Sum of electronic and thermal Energies= -1273.000594
Sum of electronic and thermal Enthalpies= -1272.999650
Sum of electronic and thermal Free Energies= -1273.078461

[Ru(salen)(CO)(P(OH)$_3$)]^0 S$_0$

Ru	-0.008109000	0.226845000	-0.289417000
C	-0.259529000	0.779753000	-2.086478000
O	-0.418029000	1.070236000	-3.189812000
P	0.187519000	-0.772980000	1.883476000
O	-0.653460000	-2.142860000	1.791808000
H	-1.103106000	-2.080904000	0.897398000
O	-0.373559000	-0.059244000	3.241951000
H	-1.316772000	0.140660000	3.175738000
O	1.650589000	-1.176351000	2.424101000
H	1.628511000	-1.577687000	3.305862000
N	-1.339629000	1.574332000	0.431528000
O	-1.538231000	-1.250726000	-0.464867000
C	-3.365715000	0.350800000	-0.139524000
C	-2.813536000	-0.918696000	-0.513630000
C	-3.719983000	-1.912637000	-0.950556000
C	-5.081183000	-1.686536000	-1.028075000
C	-5.621093000	-0.445143000	-0.665378000
C	-4.763755000	0.541249000	-0.222554000
C	-2.620959000	1.474760000	0.387874000
C	-0.666125000	2.706060000	1.068553000
N	1.313968000	1.707830000	0.072058000
O	1.506620000	-1.111208000	-0.855750000
C	3.341409000	0.427882000	-0.348301000
C	2.769203000	-0.829376000	-0.760699000
C	3.690300000	-1.860868000	-1.101524000
C	5.055940000	-1.684802000	-1.042640000
C	5.608673000	-0.457356000	-0.637912000
C	4.749520000	0.566177000	-0.302805000
C	2.596630000	1.605603000	0.016438000
C	0.644502000	2.979182000	0.335057000
H	-3.299340000	-2.874403000	-1.229907000
H	-5.734660000	-2.482893000	-1.374748000
H	-6.689290000	-0.264597000	-0.723534000
H	-5.164745000	1.506991000	0.077232000
H	-3.215686000	2.303165000	0.783680000
H	-1.301961000	3.597318000	1.080860000

H	-0.446372000	2.421153000	2.104675000
H	3.264792000	-2.810344000	-1.414384000
H	5.710628000	-2.509502000	-1.314200000
H	6.683727000	-0.318285000	-0.592747000
H	5.154405000	1.527006000	0.009008000
H	3.187804000	2.494530000	0.256132000
H	1.279294000	3.654247000	0.918911000
H	0.429197000	3.456837000	-0.629411000

Sum of electronic and zero-point Energies= -1652.494229
Sum of electronic and thermal Energies= -1652.469109
Sum of electronic and thermal Enthalpies= -1652.468164
Sum of electronic and thermal Free Energies= -1652.548996

$[Ru(salen)(CO)(P(OH)_3)]^0$ T_1

Ru	-0.016342000	0.232039000	-0.348833000
C	-0.512177000	0.716363000	-2.138524000
O	-0.852439000	0.952670000	-3.208923000
P	0.332985000	-0.644992000	1.861249000
O	-0.520791000	-2.005221000	1.924402000
H	-1.015136000	-2.019727000	1.053588000
O	-0.127618000	0.179268000	3.187974000
H	-1.078531000	0.352495000	3.196816000
O	1.827347000	-1.019169000	2.316559000
H	1.876096000	-1.343633000	3.228515000
N	-1.352127000	1.581338000	0.369127000
O	-1.518153000	-1.280312000	-0.344853000
C	-3.373196000	0.288301000	-0.053522000
C	-2.805712000	-0.990456000	-0.362968000
C	-3.698533000	-2.036156000	-0.689094000
C	-5.066695000	-1.845890000	-0.727422000
C	-5.623713000	-0.593863000	-0.431005000
C	-4.778073000	0.441828000	-0.092394000
C	-2.633560000	1.456790000	0.366026000
C	-0.684922000	2.783527000	0.871781000
N	1.261672000	1.716821000	-0.079623000
O	1.488226000	-0.990887000	-1.037126000
C	3.339584000	0.442948000	-0.312392000
C	2.768589000	-0.809901000	-0.829397000
C	3.629438000	-1.873915000	-1.168293000
C	5.003994000	-1.800434000	-1.006934000
C	5.560663000	-0.605182000	-0.498327000
C	4.763791000	0.468391000	-0.171590000
C	2.616018000	1.597098000	0.002326000
C	0.603189000	2.993846000	0.070724000
H	-3.263849000	-3.004966000	-0.917165000
H	-5.713171000	-2.678729000	-0.990998000
H	-6.697714000	-0.443528000	-0.459093000
H	-5.192020000	1.416582000	0.155335000
H	-3.230184000	2.304424000	0.714184000
H	-1.345861000	3.654793000	0.817660000
H	-0.423542000	2.605505000	1.921668000
H	3.157206000	-2.772899000	-1.556026000
H	5.639014000	-2.641397000	-1.265193000
H	6.636822000	-0.526835000	-0.364944000
H	5.218449000	1.380178000	0.208302000
H	3.167911000	2.479864000	0.324242000
H	1.246077000	3.721825000	0.579566000
H	0.351206000	3.403504000	-0.921315000

Sum of electronic and zero-point Energies= -1652.414648

Sum of electronic and thermal Energies= -1652.388992
Sum of electronic and thermal Enthalpies= -1652.388048
Sum of electronic and thermal Free Energies= -1652.470680

[Ru(salen)(P(OH)$_3$)]^0 S$_0$

Ru	-0.000273000	0.120190000	-0.323221000
P	0.084042000	-0.050706000	1.806019000
O	-0.688845000	-1.403583000	2.251351000
O	-0.557426000	1.089927000	2.778156000
O	1.533189000	-0.146702000	2.507138000
N	-1.341972000	1.620376000	-0.305311000
O	-1.517000000	-1.349900000	-0.298010000
C	-3.350513000	0.259720000	-0.545857000
C	-2.788888000	-1.059400000	-0.448806000
C	-3.693077000	-2.148202000	-0.531055000
C	-5.051445000	-1.971871000	-0.704310000
C	-5.597982000	-0.683940000	-0.805144000
C	-4.747158000	0.398349000	-0.722121000
C	-2.617708000	1.502865000	-0.456456000
C	-0.701120000	2.923978000	-0.133727000
N	1.310765000	1.616335000	-0.587172000
O	1.467255000	-1.308983000	-0.636591000
C	3.332174000	0.265262000	-0.634219000
C	2.740262000	-1.047305000	-0.620380000
C	3.639112000	-2.150477000	-0.620167000
C	5.008247000	-1.988213000	-0.624066000
C	5.580974000	-0.704462000	-0.635944000
C	4.741552000	0.388675000	-0.648084000
C	2.592662000	1.498128000	-0.698424000
C	0.652589000	2.903541000	-0.836912000
H	-1.105702000	-1.724534000	1.413519000
H	-1.482075000	1.259760000	2.554372000
H	1.478838000	-0.154572000	3.474131000
H	-3.267545000	-3.145063000	-0.456014000
H	-5.698831000	-2.843106000	-0.764259000
H	-6.664750000	-0.540759000	-0.941953000
H	-5.152442000	1.405690000	-0.791559000
H	-3.216143000	2.417100000	-0.513785000
H	-1.325883000	3.737062000	-0.519114000
H	-0.551579000	3.080709000	0.941074000
H	3.196350000	-3.142799000	-0.613344000
H	5.649450000	-2.866408000	-0.618759000
H	6.658558000	-0.576836000	-0.641839000
H	5.163616000	1.391605000	-0.669728000
H	3.178871000	2.405652000	-0.873734000
H	1.274333000	3.741796000	-0.503923000
H	0.499314000	3.003985000	-1.918570000

Sum of electronic and zero-point Energies= -1539.281334
Sum of electronic and thermal Energies= -1539.258645
Sum of electronic and thermal Enthalpies= -1539.257701
Sum of electronic and thermal Free Energies= -1539.332565

[Ru(salen)(P(OH)$_3$)]^0 T$_1$

Ru	-0.055216000	0.330793000	-0.312706000
P	0.308885000	-1.082011000	1.841414000
O	-0.735402000	-2.297688000	1.565644000
O	0.033679000	-0.774199000	3.434712000
O	1.743024000	-1.824842000	2.027100000
N	-1.395105000	1.695387000	0.309179000

O	-1.573126000	-1.054548000	-0.632124000
C	-3.415698000	0.524077000	-0.378395000
C	-2.848835000	-0.742497000	-0.733929000
C	-3.729538000	-1.742180000	-1.208840000
C	-5.085730000	-1.518003000	-1.342622000
C	-5.642450000	-0.276859000	-0.997855000
C	-4.810281000	0.712799000	-0.518932000
C	-2.679934000	1.633252000	0.173459000
C	-0.760353000	2.826596000	0.985453000
N	1.240711000	1.828907000	0.030601000
O	1.413871000	-0.754348000	-1.242774000
C	3.279615000	0.628942000	-0.522989000
C	2.691228000	-0.549424000	-1.099752000
C	3.581676000	-1.545683000	-1.582843000
C	4.950086000	-1.414176000	-1.482172000
C	5.523117000	-0.263321000	-0.911140000
C	4.689678000	0.734720000	-0.456864000
C	2.530359000	1.771008000	-0.082352000
C	0.575790000	3.104826000	0.307683000
H	-1.163213000	-2.086859000	0.697380000
H	-0.770429000	-0.252355000	3.553358000
H	1.729117000	-2.493924000	2.728511000
H	-3.294240000	-2.701996000	-1.471993000
H	-5.724119000	-2.313724000	-1.717372000
H	-6.708228000	-0.100578000	-1.099494000
H	-5.225913000	1.677125000	-0.235318000
H	-3.275878000	2.483654000	0.516909000
H	-1.399212000	3.716489000	0.969714000
H	-0.591390000	2.539043000	2.030022000
H	3.136659000	-2.430922000	-2.028272000
H	5.591621000	-2.209809000	-1.852594000
H	6.600870000	-0.159870000	-0.839050000
H	5.113653000	1.640695000	-0.028897000
H	3.106635000	2.672120000	0.147210000
H	1.202053000	3.753773000	0.929761000
H	0.400226000	3.612870000	-0.648428000

Sum of electronic and zero-point Energies= -1539.238341
Sum of electronic and thermal Energies= -1539.214522
Sum of electronic and thermal Enthalpies= -1539.213577
Sum of electronic and thermal Free Energies= -1539.293831

[Ru(salen)(H_2O)($P(OH)_3$)]^0 S_0

Ru	-0.034539000	0.033993000	-0.282232000
P	0.120979000	0.412276000	1.849029000
O	-1.303021000	0.499099000	2.642884000
O	0.853871000	1.808553000	2.279268000
O	0.942347000	-0.714093000	2.696350000
N	-1.366181000	1.520271000	-0.508801000
O	-1.533918000	-1.397824000	-0.029602000
C	-3.380457000	0.157532000	-0.423925000
C	-2.795688000	-1.133213000	-0.149740000
C	-3.708247000	-2.220767000	-0.014826000
C	-5.072383000	-2.068605000	-0.139188000
C	-5.634708000	-0.808696000	-0.408557000
C	-4.785867000	0.269141000	-0.543294000
C	-2.647425000	1.388641000	-0.579009000
C	-0.722146000	2.830722000	-0.568429000
N	1.287103000	1.462553000	-0.805070000
O	1.457467000	-1.450283000	-0.387904000

C	3.310383000	0.135039000	-0.554413000
C	2.729986000	-1.165745000	-0.347302000
C	3.636198000	-2.234139000	-0.109909000
C	5.003918000	-2.052393000	-0.070669000
C	5.565012000	-0.781991000	-0.275425000
C	4.716331000	0.277881000	-0.520258000
C	2.568232000	1.334607000	-0.865892000
C	0.627771000	2.687771000	-1.266622000
H	-1.249781000	0.240652000	3.573113000
H	0.553800000	2.158787000	3.129196000
H	1.345262000	-0.378801000	3.508934000
H	-3.275093000	-3.195679000	0.192895000
H	-5.717865000	-2.936703000	-0.026662000
H	-6.708564000	-0.686620000	-0.505855000
H	-5.199428000	1.254993000	-0.748605000
H	-3.245559000	2.286682000	-0.763218000
H	-1.347900000	3.565913000	-1.086562000
H	-0.561289000	3.173640000	0.460513000
H	3.201803000	-3.218132000	0.044572000
H	5.650182000	-2.905851000	0.120093000
H	6.640108000	-0.637200000	-0.248315000
H	5.130676000	1.269192000	-0.694134000
H	3.159949000	2.199075000	-1.183383000
H	1.252937000	3.569858000	-1.089348000
H	0.466598000	2.602915000	-2.348895000
O	-0.077376000	-0.654489000	-2.471128000
H	0.383863000	-0.034447000	-3.048968000
H	0.598459000	-1.310274000	-2.219688000

Sum of electronic and zero-point Energies= -1615.609583
Sum of electronic and thermal Energies= -1615.583458
Sum of electronic and thermal Enthalpies= -1615.582514
Sum of electronic and thermal Free Energies= -1615.665612

$[Ru(salen)(CO)(PF_3)]^0$ S_0

Ru	-0.022027000	0.248172000	-0.338840000
C	-0.245835000	0.932769000	-2.086460000
O	-0.387860000	1.309570000	-3.161855000
P	0.172374000	-0.802661000	1.775292000
F	1.074466000	-2.086602000	1.901433000
F	-1.158605000	-1.380443000	2.389024000
F	0.692816000	-0.013681000	3.042220000
N	-1.347013000	1.569546000	0.450672000
O	-1.519715000	-1.181062000	-0.675045000
C	-3.364349000	0.322636000	-0.093342000
C	-2.786153000	-0.900131000	-0.590336000
C	-3.698132000	-1.903850000	-1.020857000
C	-5.064525000	-1.728729000	-0.979554000
C	-5.624690000	-0.533327000	-0.496763000
C	-4.773885000	0.457735000	-0.060619000
C	-2.628934000	1.438908000	0.442439000
C	-0.677354000	2.678839000	1.129396000
N	1.311040000	1.702219000	0.128342000
O	1.486956000	-1.057779000	-0.975995000
C	3.326904000	0.401046000	-0.275006000
C	2.750097000	-0.813074000	-0.796143000
C	3.662968000	-1.844980000	-1.154185000
C	5.026492000	-1.710648000	-1.007701000
C	5.584600000	-0.526505000	-0.495043000
C	4.734199000	0.498306000	-0.145221000

C	2.593228000	1.579536000	0.109416000
C	0.646521000	2.968681000	0.426170000
H	-3.265980000	-2.828580000	-1.392528000
H	-5.713794000	-2.529363000	-1.325345000
H	-6.700402000	-0.396986000	-0.462331000
H	-5.185300000	1.387199000	0.327658000
H	-3.225024000	2.239991000	0.889381000
H	-1.309199000	3.572552000	1.154684000
H	-0.475386000	2.373944000	2.164428000
H	3.232730000	-2.760136000	-1.550915000
H	5.675589000	-2.534277000	-1.294892000
H	6.658332000	-0.420824000	-0.381694000
H	5.144081000	1.427350000	0.245625000
H	3.191828000	2.445860000	0.405837000
H	1.277533000	3.617421000	1.042776000
H	0.450287000	3.481274000	-0.524273000

Sum of electronic and zero-point Energies= -1724.533186
Sum of electronic and thermal Energies= -1724.508677
Sum of electronic and thermal Enthalpies= -1724.507733
Sum of electronic and thermal Free Energies= -1724.589368

$[Ru(salen)(CO)(PF_3)]^0$ T_1

Ru	-0.145809000	-0.041276000	-0.261642000
C	-0.908468000	-0.606804000	-1.930166000
O	-1.343748000	-0.978642000	-2.919402000
P	0.716574000	0.622103000	1.857265000
F	1.782499000	-0.260793000	2.604840000
F	-0.342376000	0.762729000	3.007295000
F	1.414923000	2.028707000	1.981679000
N	-1.334286000	1.590627000	-0.211422000
O	-1.543988000	-1.006549000	0.807164000
C	-3.381997000	0.247816000	-0.193968000
C	-2.828456000	-0.920983000	0.459227000
C	-3.666402000	-1.995231000	0.786887000
C	-5.020966000	-1.989400000	0.475064000
C	-5.571199000	-0.871370000	-0.175957000
C	-4.775994000	0.209254000	-0.493699000
C	-2.651178000	1.417022000	-0.503454000
C	-0.648056000	2.803839000	-0.624145000
N	1.144608000	1.276716000	-1.162312000
O	1.315193000	-1.523970000	-0.444861000
C	3.176768000	0.006755000	-0.770725000
C	2.592715000	-1.264897000	-0.442315000
C	3.482382000	-2.325785000	-0.133147000
C	4.849408000	-2.146001000	-0.117985000
C	5.419602000	-0.900018000	-0.433127000
C	4.585487000	0.144159000	-0.764509000
C	2.428295000	1.157768000	-1.199128000
C	0.441655000	2.457935000	-1.647401000
H	-3.211724000	-2.845774000	1.288003000
H	-5.643375000	-2.841021000	0.732181000
H	-6.628872000	-0.852045000	-0.424365000
H	-5.213133000	1.075720000	-0.985068000
H	-3.186798000	2.259218000	-0.942361000
H	-1.349517000	3.524893000	-1.058302000
H	-0.175640000	3.279062000	0.245787000
H	3.041154000	-3.289371000	0.102940000
H	5.492660000	-2.983934000	0.137535000
H	6.496153000	-0.767297000	-0.426021000

H	5.006229000	1.110990000	-1.030870000
H	3.006307000	1.997714000	-1.594824000
H	1.133192000	3.293089000	-1.805837000
H	-0.028555000	2.205097000	-2.605614000

Sum of electronic and zero-point Energies= -1724.453314
Sum of electronic and thermal Energies= -1724.428452
Sum of electronic and thermal Enthalpies= -1724.427508
Sum of electronic and thermal Free Energies= -1724.509840

[Ru(salen)(PF$_3$)]^0 S$_0$

Ru	-0.029611000	0.076426000	-0.332871000
P	0.123929000	0.104757000	1.741505000
F	1.364782000	-0.582650000	2.439540000
F	-1.005395000	-0.577177000	2.613277000
F	0.184206000	1.479839000	2.526443000
N	-1.370525000	1.583350000	-0.416137000
O	-1.504466000	-1.363679000	-0.446446000
C	-3.375677000	0.209261000	-0.510278000
C	-2.778270000	-1.099940000	-0.469275000
C	-3.669609000	-2.207857000	-0.486450000
C	-5.038139000	-2.052734000	-0.539045000
C	-5.617772000	-0.772606000	-0.579699000
C	-4.785851000	0.324810000	-0.567416000
C	-2.651912000	1.451543000	-0.508953000
C	-0.731777000	2.895457000	-0.317399000
N	1.288272000	1.570096000	-0.681301000
O	1.432792000	-1.361154000	-0.597702000
C	3.302218000	0.209705000	-0.635707000
C	2.706707000	-1.099052000	-0.566448000
C	3.600061000	-2.203034000	-0.496513000
C	4.969110000	-2.043110000	-0.486318000
C	5.546552000	-0.762849000	-0.552711000
C	4.712791000	0.330368000	-0.634217000
C	2.568938000	1.437544000	-0.784182000
C	0.626210000	2.837883000	-1.009659000
H	-3.221470000	-3.196981000	-0.457211000
H	-5.673940000	-2.934575000	-0.548801000
H	-6.695171000	-0.651710000	-0.620389000
H	-5.212764000	1.325041000	-0.600006000
H	-3.253771000	2.361785000	-0.585702000
H	-1.355012000	3.679546000	-0.759651000
H	-0.595127000	3.122216000	0.746191000
H	3.153756000	-3.192311000	-0.448992000
H	5.607521000	-2.921180000	-0.427830000
H	6.624444000	-0.639304000	-0.547700000
H	5.137923000	1.329679000	-0.700956000
H	3.158146000	2.329173000	-1.019014000
H	1.244612000	3.694958000	-0.722458000
H	0.480168000	2.871683000	-2.096193000

Sum of electronic and zero-point Energies= -1611.327203
Sum of electronic and thermal Energies= -1611.306384
Sum of electronic and thermal Enthalpies= -1611.305440
Sum of electronic and thermal Free Energies= -1611.376798

[Ru(salen)(PF$_3$)]^0 T$_1$

Ru	-0.116999000	-0.088004000	-0.325666000
P	0.558238000	0.631998000	1.855960000
F	1.391929000	0.125293000	3.134940000
F	-0.761227000	1.021722000	2.663406000

F	1.191487000	2.097500000	1.812163000
N	-1.425738000	1.403909000	-0.698114000
O	-1.623433000	-1.295146000	0.326484000
C	-3.453893000	0.074762000	-0.500992000
C	-2.882132000	-1.131070000	0.029416000
C	-3.766133000	-2.212324000	0.281126000
C	-5.117297000	-2.123228000	0.021994000
C	-5.676716000	-0.940636000	-0.493851000
C	-4.848186000	0.131771000	-0.738588000
C	-2.711037000	1.269723000	-0.781831000
C	-0.774161000	2.686722000	-0.953582000
N	1.175744000	1.258801000	-1.143776000
O	1.295761000	-1.546863000	-0.559806000
C	3.190464000	-0.051556000	-0.812764000
C	2.580810000	-1.323923000	-0.543421000
C	3.446646000	-2.417137000	-0.286210000
C	4.817722000	-2.267371000	-0.258252000
C	5.412876000	-1.020086000	-0.514996000
C	4.600919000	0.055187000	-0.802985000
C	2.457455000	1.116008000	-1.222984000
C	0.503369000	2.413626000	-1.739699000
H	-3.332548000	-3.121762000	0.686559000
H	-5.756165000	-2.979109000	0.223828000
H	-6.741493000	-0.873035000	-0.690659000
H	-5.262684000	1.058051000	-1.130142000
H	-3.294167000	2.143228000	-1.085710000
H	-1.432325000	3.368947000	-1.501465000
H	-0.524436000	3.142623000	0.011248000
H	2.985184000	-3.381808000	-0.096722000
H	5.443368000	-3.128783000	-0.039290000
H	6.492067000	-0.909492000	-0.499836000
H	5.042754000	1.023085000	-1.029048000
H	3.040536000	1.930132000	-1.662321000
H	1.156511000	3.292500000	-1.750533000
H	0.245174000	2.161268000	-2.775086000

Sum of electronic and zero-point Energies= -1611.289797
Sum of electronic and thermal Energies= -1611.267208
Sum of electronic and thermal Enthalpies= -1611.266264
Sum of electronic and thermal Free Energies= -1611.344282

$[Ru(salen)(H_2O)(PF_3)]^0$ S_0

Ru	-0.032781000	0.071477000	-0.270336000
P	0.137731000	0.179357000	1.838082000
F	1.188051000	-0.737971000	2.589227000
F	-1.113475000	-0.174412000	2.744914000
F	0.518315000	1.544644000	2.554863000
N	-1.367788000	1.581905000	-0.351790000
O	-1.520833000	-1.378412000	-0.180498000
C	-3.374809000	0.206520000	-0.388231000
C	-2.786280000	-1.103070000	-0.255891000
C	-3.691659000	-2.202237000	-0.222944000
C	-5.057115000	-2.041293000	-0.313373000
C	-5.625741000	-0.762067000	-0.443976000
C	-4.782934000	0.326738000	-0.478142000
C	-2.648263000	1.448536000	-0.427603000
C	-0.723317000	2.891687000	-0.282986000
N	1.288306000	1.556420000	-0.650828000
O	1.440425000	-1.388002000	-0.523484000
C	3.301927000	0.198081000	-0.530026000

C	2.714761000	-1.113368000	-0.454680000
C	3.612578000	-2.206534000	-0.329312000
C	4.980595000	-2.036476000	-0.273488000
C	5.550004000	-0.754742000	-0.347115000
C	4.709779000	0.329546000	-0.482302000
C	2.568917000	1.424997000	-0.722113000
C	0.627031000	2.819684000	-0.991622000
H	-3.253547000	-3.191496000	-0.123551000
H	-5.699410000	-2.918190000	-0.283119000
H	-6.700963000	-0.635194000	-0.514250000
H	-5.200784000	1.326770000	-0.576239000
H	-3.250666000	2.357260000	-0.520040000
H	-1.349414000	3.672450000	-0.727584000
H	-0.567685000	3.136509000	0.774478000
H	3.172226000	-3.198239000	-0.275919000
H	5.621952000	-2.908509000	-0.172214000
H	6.625927000	-0.621104000	-0.306008000
H	5.129805000	1.330666000	-0.555049000
H	3.164768000	2.313293000	-0.952999000
H	1.250867000	3.680050000	-0.726817000
H	0.467376000	2.842208000	-2.076992000
O	-0.149223000	-0.307842000	-2.483204000
H	0.265374000	0.383103000	-3.014710000
H	0.505138000	-1.025492000	-2.447820000

Sum of electronic and zero-point Energies= -1687.668657
Sum of electronic and thermal Energies= -1687.644368
Sum of electronic and thermal Enthalpies= -1687.643424
Sum of electronic and thermal Free Energies= -1687.722964

$[Ru(salen)(CO)(PH_3)]^0$ S_0

Ru	0.012116000	0.085852000	0.111182000
C	0.217410000	0.098842000	1.963103000
O	0.348383000	0.074467000	3.110858000
P	-0.282120000	-0.152302000	-2.335390000
H	-0.049895000	-1.454892000	-2.820182000
H	-1.553655000	0.071266000	-2.904320000
H	0.497287000	0.567373000	-3.266901000
N	1.336792000	1.594585000	-0.164243000
O	1.522961000	-1.363415000	-0.086834000
C	3.361983000	0.249576000	-0.071545000
C	2.785572000	-1.071796000	-0.035781000
C	3.703163000	-2.158863000	0.043231000
C	5.068225000	-1.974868000	0.088882000
C	5.624303000	-0.684112000	0.054400000
C	4.769670000	0.393140000	-0.031162000
C	2.619084000	1.477187000	-0.201233000
C	0.668355000	2.867086000	-0.429910000
N	-1.325478000	1.607065000	0.163289000
O	-1.512027000	-1.364071000	0.175082000
C	-3.348408000	0.254739000	0.093051000
C	-2.774899000	-1.067967000	0.131102000
C	-3.694925000	-2.155547000	0.113199000
C	-5.059687000	-1.972341000	0.057304000
C	-5.613203000	-0.680885000	0.016865000
C	-4.755894000	0.397351000	0.037177000
C	-2.607552000	1.490733000	0.131768000
C	-0.663624000	2.900035000	0.315679000
H	3.275193000	-3.157257000	0.067625000
H	5.720150000	-2.842948000	0.151717000

H	6.699120000	-0.539847000	0.088024000
H	5.177696000	1.401325000	-0.070025000
H	3.210631000	2.385343000	-0.353448000
H	1.295625000	3.717769000	-0.142196000
H	0.478769000	2.934038000	-1.509319000
H	-3.268601000	-3.154441000	0.143941000
H	-5.712913000	-2.841601000	0.044871000
H	-6.687620000	-0.536236000	-0.025888000
H	-5.162054000	1.406704000	0.012722000
H	-3.203463000	2.408534000	0.144985000
H	-1.291045000	3.719612000	-0.051048000
H	-0.477706000	3.061056000	1.385338000

Sum of electronic and zero-point Energies= -1426.969403
Sum of electronic and thermal Energies= -1426.946966
Sum of electronic and thermal Enthalpies= -1426.946022
Sum of electronic and thermal Free Energies= -1427.021528

[Ru(salen)(CO)(PH_3)]^0 T_1

Ru	0.015747000	0.036109000	0.133515000
C	0.652302000	0.422732000	2.044198000
O	0.719962000	1.360346000	2.739385000
P	-0.659277000	-0.580490000	-2.402026000
H	-1.012175000	-1.902079000	-2.768123000
H	-1.654133000	0.072365000	-3.169829000
H	0.388896000	-0.432357000	-3.340908000
N	1.318611000	1.494310000	-0.438819000
O	1.492181000	-1.366838000	-0.170221000
C	3.350347000	0.194632000	-0.155262000
C	2.764806000	-1.112729000	-0.052300000
C	3.650041000	-2.204090000	0.143590000
C	5.013445000	-2.025378000	0.255607000
C	5.584093000	-0.744863000	0.156251000
C	4.754317000	0.334136000	-0.059701000
C	2.605446000	1.390531000	-0.445580000
C	0.648394000	2.727510000	-0.840682000
N	-1.276742000	1.596766000	0.066696000
O	-1.499156000	-1.179994000	0.796071000
C	-3.318134000	0.282016000	0.126303000
C	-2.756335000	-0.980878000	0.517792000
C	-3.651354000	-2.074099000	0.653936000
C	-5.001314000	-1.944880000	0.403134000
C	-5.549394000	-0.708093000	0.020232000
C	-4.711405000	0.378894000	-0.099404000
C	-2.564561000	1.501471000	0.019466000
C	-0.598844000	2.887787000	0.023004000
H	3.208708000	-3.194180000	0.211404000
H	5.652252000	-2.889669000	0.418590000
H	6.657545000	-0.609847000	0.237557000
H	5.177382000	1.331492000	-0.158104000
H	3.189248000	2.276553000	-0.710337000
H	1.312484000	3.592476000	-0.742282000
H	0.353839000	2.633660000	-1.893252000
H	-3.227497000	-3.026593000	0.958387000
H	-5.647991000	-2.812238000	0.508130000
H	-6.613204000	-0.609825000	-0.168874000
H	-5.118125000	1.348155000	-0.379323000
H	-3.141396000	2.422408000	-0.102471000
H	-1.253863000	3.671952000	-0.370242000
H	-0.303757000	3.152441000	1.044885000

Sum of electronic and zero-point Energies= -1426.907874
Sum of electronic and thermal Energies= -1426.884056
Sum of electronic and thermal Enthalpies= -1426.883112
Sum of electronic and thermal Free Energies= -1426.963344

[Ru(salen)(PH_3)]^0 S_0

Ru	-0.012090000	0.086213000	-0.097580000
P	0.174042000	0.011899000	2.077308000
N	-1.348884000	1.588627000	-0.069924000
O	-1.512763000	-1.354153000	-0.171065000
C	-3.368710000	0.237660000	-0.193506000
C	-2.781115000	-1.079374000	-0.197828000
C	-3.687727000	-2.176789000	-0.256652000
C	-5.054778000	-2.008590000	-0.307476000
C	-5.621814000	-0.722221000	-0.303476000
C	-4.777204000	0.365451000	-0.248099000
C	-2.634329000	1.475494000	-0.140588000
C	-0.707396000	2.893707000	0.085983000
N	1.306691000	1.584583000	-0.344212000
O	1.477487000	-1.348389000	-0.347313000
C	3.331945000	0.241347000	-0.295315000
C	2.745577000	-1.076279000	-0.287720000
C	3.653486000	-2.172773000	-0.234885000
C	5.020439000	-2.002161000	-0.185550000
C	5.585786000	-0.714973000	-0.192043000
C	4.740378000	0.371864000	-0.253630000
C	2.592370000	1.472747000	-0.406620000
C	0.648842000	2.867310000	-0.612362000
H	-0.940484000	-0.442806000	2.821140000
H	1.174130000	-0.840670000	2.601516000
H	0.466581000	1.191179000	2.802002000
H	-3.249347000	-3.171146000	-0.260819000
H	-5.698579000	-2.883912000	-0.351017000
H	-6.697927000	-0.588283000	-0.342926000
H	-5.195178000	1.370438000	-0.244505000
H	-3.231701000	2.392426000	-0.154254000
H	-1.330791000	3.701067000	-0.314149000
H	-0.563883000	3.075963000	1.158782000
H	3.216819000	-3.167888000	-0.232241000
H	5.665879000	-2.876265000	-0.141943000
H	6.661905000	-0.580008000	-0.155812000
H	5.157214000	1.377170000	-0.272241000
H	3.182793000	2.379885000	-0.570018000
H	1.268584000	3.711242000	-0.289205000
H	0.498359000	2.953098000	-1.695802000

Sum of electronic and zero-point Energies= -1313.747157
Sum of electronic and thermal Energies= -1313.727425
Sum of electronic and thermal Enthalpies= -1313.726481
Sum of electronic and thermal Free Energies= -1313.795775

[Ru(salen)(PH_3)]^0 T_1

Ru	-0.050617000	0.070666000	-0.064232000
N	-1.374766000	1.576800000	-0.113577000
O	-1.550680000	-1.322927000	0.113754000
C	-3.396959000	0.229404000	-0.237235000
C	-2.813212000	-1.077152000	-0.078110000
C	-3.701965000	-2.187355000	-0.117344000
C	-5.059190000	-2.035787000	-0.299012000
C	-5.626967000	-0.757122000	-0.449835000

C	-4.798155000	0.341596000	-0.413609000
C	-2.662943000	1.462056000	-0.213383000
C	-0.738566000	2.890003000	-0.015709000
N	1.258908000	1.563210000	-0.429163000
O	1.408797000	-1.327970000	-0.438744000
C	3.284958000	0.222846000	-0.397927000
C	2.687361000	-1.086084000	-0.401255000
C	3.573081000	-2.197832000	-0.393675000
C	4.942586000	-2.044366000	-0.353403000
C	5.523120000	-0.763520000	-0.342544000
C	4.695529000	0.337314000	-0.378396000
C	2.546186000	1.450487000	-0.512785000
C	0.600150000	2.833678000	-0.743431000
H	-3.261288000	-3.172966000	0.003815000
H	-5.696448000	-2.916260000	-0.323423000
H	-6.696610000	-0.639426000	-0.589446000
H	-5.218795000	1.338895000	-0.524693000
H	-3.254763000	2.379753000	-0.279667000
H	-1.374418000	3.678212000	-0.434356000
H	-0.573040000	3.109918000	1.046000000
H	3.122358000	-3.186227000	-0.407709000
H	5.578331000	-2.926018000	-0.332904000
H	6.601438000	-0.644767000	-0.317024000
H	5.125732000	1.336656000	-0.390833000
H	3.131327000	2.352487000	-0.716177000
H	1.229630000	3.688329000	-0.471354000
H	0.426099000	2.868161000	-1.825822000
P	0.565432000	0.160341000	2.488631000
H	1.085069000	-0.923146000	3.240392000
H	1.547338000	1.106841000	2.867924000
H	-0.410308000	0.553227000	3.437039000

Sum of electronic and zero-point Energies= -1313.721234
Sum of electronic and thermal Energies= -1313.700016
Sum of electronic and thermal Enthalpies= -1313.699072
Sum of electronic and thermal Free Energies= -1313.773863

[Ru(salen)(H_2O)(PH_3)]^0 S_0

Ru	-0.016230000	0.094613000	-0.016440000
P	0.153799000	0.099586000	2.206014000
N	-1.350631000	1.596035000	0.001055000
O	-1.524299000	-1.357628000	0.046346000
C	-3.370585000	0.241090000	-0.087774000
C	-2.786900000	-1.077840000	-0.025137000
C	-3.701281000	-2.172065000	-0.054440000
C	-5.066438000	-2.000596000	-0.136204000
C	-5.627767000	-0.713203000	-0.195453000
C	-4.777063000	0.371466000	-0.169770000
C	-2.634630000	1.480861000	-0.060245000
C	-0.707979000	2.901023000	0.146003000
N	1.306562000	1.587034000	-0.275084000
O	1.483844000	-1.358345000	-0.286781000
C	3.331344000	0.241248000	-0.177032000
C	2.754410000	-1.078347000	-0.185962000
C	3.662997000	-2.168366000	-0.101842000
C	5.028695000	-1.991895000	-0.009752000
C	5.586140000	-0.703441000	-0.001684000
C	4.736002000	0.379233000	-0.092811000
C	2.591317000	1.475104000	-0.311129000
C	0.651065000	2.869167000	-0.548702000

H	-1.024576000	0.047138000	2.989727000
H	0.851096000	-0.979250000	2.799431000
H	0.801299000	1.165709000	2.876639000
H	-3.268773000	-3.168128000	-0.008573000
H	-5.713283000	-2.874772000	-0.154634000
H	-6.702357000	-0.575794000	-0.258445000
H	-5.190122000	1.377808000	-0.212500000
H	-3.232561000	2.397453000	-0.083980000
H	-1.329384000	3.705965000	-0.262521000
H	-0.565487000	3.094039000	1.217203000
H	3.231264000	-3.165586000	-0.111084000
H	5.675972000	-2.863135000	0.056560000
H	6.659737000	-0.561556000	0.067834000
H	5.148280000	1.386527000	-0.100986000
H	3.186660000	2.381139000	-0.462071000
H	1.271194000	3.713905000	-0.228455000
H	0.503164000	2.954072000	-1.632760000
O	-0.040831000	-0.259018000	-2.259574000
H	0.407998000	0.449128000	-2.737487000
H	0.639809000	-0.943598000	-2.130865000

Sum of electronic and zero-point Energies= -1390.087906

Sum of electronic and thermal Energies= -1390.065585

Sum of electronic and thermal Enthalpies= -1390.064640

Sum of electronic and thermal Free Energies= -1390.138898
